# Rhodium(ii)-catalyzed C–H aminations using *N*-mesyloxycarbamates: reaction pathway and by-product formation†
†Electronic supplementary information (ESI) available: DFT-optimized geometries; tables containing Δ*E*, EZPE, imaginary frequencies, Δ*H*, and Δ*G* values for all of the DFT-optimized geometries. See DOI: 10.1039/c8sc03153c


**DOI:** 10.1039/c8sc03153c

**Published:** 2018-10-22

**Authors:** Emna Azek, Maroua Khalifa, Johan Bartholoméüs, Matthias Ernzerhof, Hélène Lebel

**Affiliations:** a Département de Chimie , Université de Montréal , C.P. 6128, Succursale Centre-ville, Montréal , Québec , Canada H3C3J7 . Email: helene.lebel@umontreal.ca

## Abstract

*N*-Mesyloxycarbamates are practical nitrene precursors that undergo C–H amination reactions in the presence of rhodium dimer catalysts. Under these conditions, both oxazolidinones and chiral amines have been prepared in a highly efficient manner. Given the elevated reactivity of the intermediates involved in the catalytic cycle, mechanistic details have remained hypothetical, relying on indirect experiments. Herein a density functional theory (DFT) study is presented to validate the catalytic cycle of the rhodium-catalyzed C–H amination with *N*-mesyloxycarbamates. A concerted pathway involving Rh–nitrene species that undergoes C–H insertion is found to be favored over a stepwise C–N bond formation manifold. Density functional calculations and kinetic studies suggest that the rate-limiting step is the C–H insertion process rather than the formation of Rh–nitrene species. In addition, these studies provide mechanistic details about competitive by-product formation, resulting from an intermolecular reaction between the Rh–nitrene species and the *N*-mesyloxycarbamate anion.

## Abstract 2

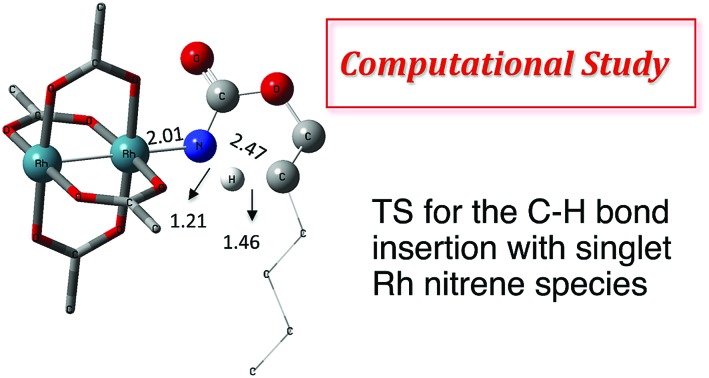
DFT study to elucidate the mechanism of Rh-catalyzed C–H aminations with *N*-mesyloxycarbamates and the pathway by which by-products formed.

## Introduction

**Scheme 1:**
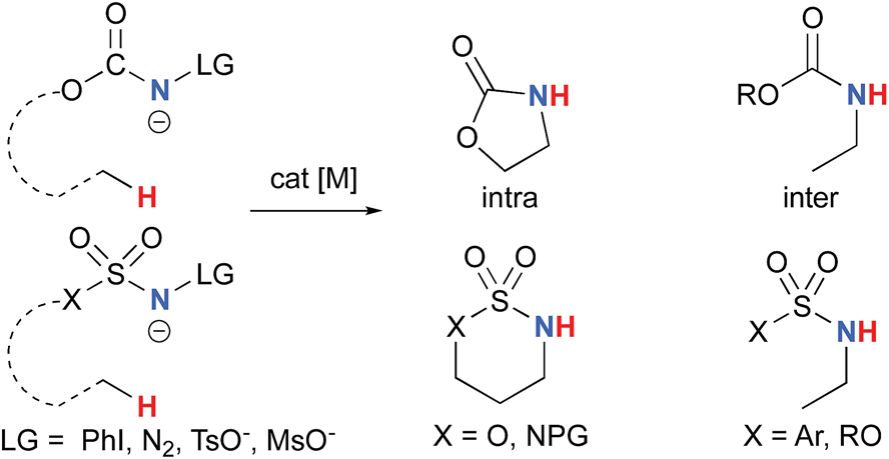
Metal-catalyzed C–H amination reactions.

The synthesis of amines by the functionalization of C–H bonds has emerged as an efficient strategy to prepare molecules of relevance to many chemical areas, including the pharmaceutical industry.^1^–^4^ Significant advantages are associated with metal-catalyzed C–H amination *via* nitrene insertion, including mild reaction conditions as well as high chemo- and stereoselectivities.^5^,^6^ Among these processes, intramolecular metal-catalyzed C–H aminations with carbamate derivatives afford oxazolidinones in good to excellent yields,^7^–^12^ whereas intermolecular reactions produce amines bearing readily removable carbamate protecting groups (Scheme 1).^13^–^16^ Very recently, the synthesis of γ-lactams *via* C–H amidation was reported using tailored iridium catalysts.^17^ Alternatively, intramolecular cyclization with sulfamate derivatives has furnished the corresponding 6-membered oxathiazinane heterocycle intramolecularly.^18^–^31^

**Scheme 2:**
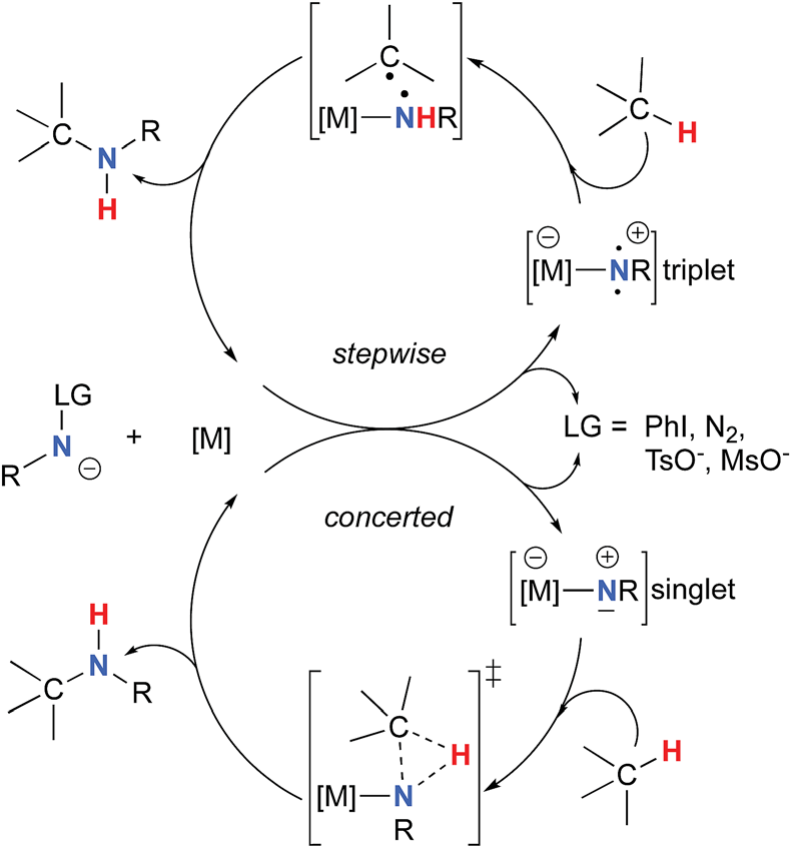
Metal-catalyzed C–H amination reactions.

The synthesis of sulfonyl-protected amines has also been achieved *via* intermolecular C–H amination processes.^32^–^42^ More recently, aryl and alkyl azides have been reported in metal-catalyzed intra- and intermolecular processes to afford various heterocyclic and amine products.^43^–^54^ Rhodium dimer complexes^7^,^10^–^15^,^18^–^20^,^33^–^37^,^43^–^46^ are the most reported catalysts, although Co,^16^,^28^–^30^,^48^–^51^ Cu,^38^–^41^,^49^ Ir,^17^,^31^ Fe,^24^,^25^,^47^,^53^,^54^ Mn,^27^,^32^ Ru^21^–^23^,^32^,^42^ and Ag^8^,^9^,^26^-catalyzed C–H amination processes have also been delineated. Most commonly used nitrene precursors include iminoiodinanes,^7^–^9^,^18^–^27^,^32^–^41^ azides,^16^,^28^–^31^,^42^–^54^ and *N*-sulfonyloxycarbamates.^10^–^15^ The generally accepted mechanism involves the formation of a metal nitrene species, which undergoes C–H insertion to form the observed product (Scheme 2).^1^–^6^ Two different mechanisms have been proposed for the C–H insertion/C–N formation step: a concerted C–H insertion, proceeding *via* a hydride transfer/C–N formation transition state and a stepwise process of hydrogen atom abstraction, followed by radical recombination. Due to their high reactivity, only a limited number of discrete metal–nitrene intermediates have been isolated.^28^,^55^–^58^ Consequently, in addition to various experimental analyses, density functional theory (DFT) studies have been instrumental in establishing the exact mechanism of metal nitrene C–H insertion reactions. With rhodium(ii) dimer complexes, such as Rh_2_(OAc)_4_, using carbamate- and sulfamate-derived iminoiodinane reagents, a concerted asynchronous insertion of a singlet Rh(ii)–nitrene has been found in previous computational studies,^59^,^60^ in agreement with what was observed experimentally.^33^,^58^,^61^ For Rh_2_(esp)_2_, an alternative one-electron mechanism involving Rh(ii)–Rh(iii) intermediates has been proposed with a concerted C–H insertion step.^62^–^64^ Conversely, computational and experimental data have established a stepwise mechanism for the C–H amination catalyzed by a diruthenium complex involving a short-lived diradical species.^23^,^65^

Calculations have suggested a substrate-based mechanism for Ru(ii)–porphyrin^66^–^69^ and –pybox^70^ complexes, as the C–H insertion can also proceed *via* a concerted or a stepwise mechanism. In the case of first row metal complexes reacting with azide reagents, experimental and computational studies imply an open-shell electronic state of the metal nitrene (Co,^49^–^51^,^71^–^73^ Cu,^40^,^74^,^75^ Fe,^25^,^76^,^77^ Ni^57^), which reacts *via* a stepwise sequence.^78^ The first step involves a H-abstraction transition state and affords a radical intermediate that undergoes the C–N bond formation, which in some cases is a barrier-free process.^74^

Most computational studies had focused on the structure and reactivity of the metal nitrene species. As a result, little attention has been devoted to the formation of the metal nitrene species, and the type of nitrene precursors applied in C–H amination reactions. Namely, the rate-determining step is unknown for pre-oxidized metal nitrene precursors, such as *N*-sulfonyloxycarbamates. Furthermore, no studies have been conducted to establish the mechanism by which some particular substrates afforded ketones as by-products. Understanding the pathway producing these products may lead to ways to control the reaction to avoid their formation. Herein, the results of an in-depth mechanistic study with *N*-sulfonyloxycarbamates, known to produce oxazolidinones^10^–^12^ and carbamate-protected amines^13^–^15^ in high yields and selectivities (eqn (1) and(2)), are reported. The nature of the active amination reagent (metal nitrenoid *vs.* metal nitrene), the resting state of the catalyst, the rate-determining step, the mechanism (stepwise or concerted) and the pathway responsible for the formation of the by-products will be addressed in this computational study.

## Results and discussion

### Computational methods

A number of approximations were tested to determine their accuracy in reproducing the Rh_2_(OAc)_4_ crystal structure,^79^,^80^ the catalyst chosen for the DFT study (*vide infra*) (Table 1). The pure Perdew–Burke–Ernzerhof (PBE) functional^81^ was shown to best approximate the known X-ray data.
1

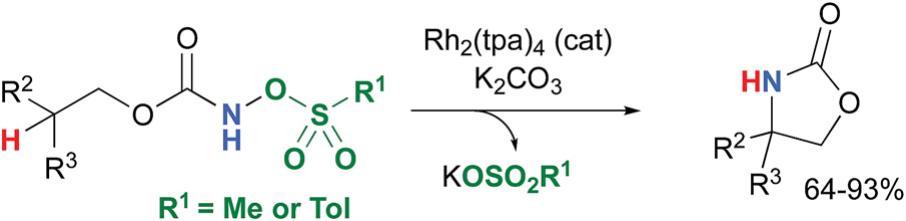



2

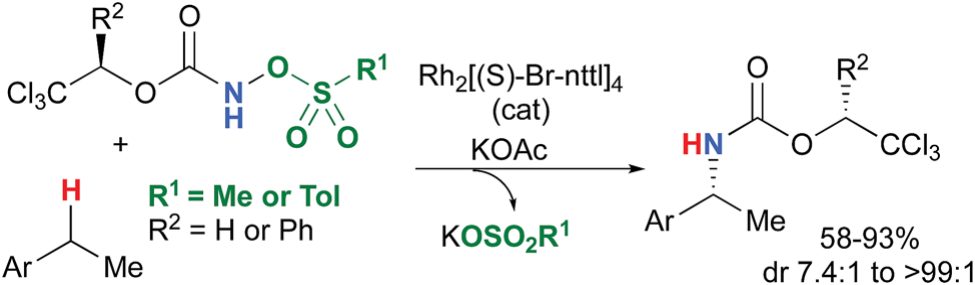




**Table 1:** Geometrical parameters for Rh_2_(OAc)_4_ with different computational methods^*a*^

Parameter °/Å	HF	LDA	BPW91	BLYP	PBE	PBE1	B3LYP	X-Ray
Rh–Rh	2.355	2.349	2.380	2.404	2.380	2.355	2.378	**2.385**
Rh–O	2.054	1.996	2.051	2.051	2.047	2.028	2.050	**2.039**
C–O	1.249	1.276	1.276	1.286	1.286	1.269	1.274	**1.269**
C–C(CH_3_)	1.504	1.489	1.489	1.521	1.511	1.502	1.510	**1.499**
O–Rh–Rh	87.9	88.8	88.7	88.6	88.7	88.6	88.4	**88.1**
O–C–O	124.6	125.6	125.6	125.9	126.0	125.7	125.5	**124.8**

^*a*^See the ESI for the basis sets.

The singlet–triplet energy difference (Δ*E*_st_) of the dirhodium–nitrene species ((HO(O)C)_4_Rh_2_=NH) was calculated to be 1.4 kcal mol^–1^ with the Coupled-cluster (with) Single (and) Double (and Perturbative) Triple (excitations) (CCSD(T)) method,^59^ the most accurate level of computation. In comparison, the pure PBE functional method provided a value of 1.2 kcal mol^–1^, the closest among all other computational methods studied. Conversely, hybrid functional methods afforded singlet–triplet energy difference values superior to 10 kcal mol^–1^.^82^ Explicit relativistic effects treatment was also tested, but provided a less accurate value (0.8 kcal mol^–1^ with PBE-DKH/BS3).^82^ The reaction and activation energies were thus calculated using Kohn–Sham density functional theory (DFT) with the PBE approximation for the exchange–correlation energy. To evaluate the effect of solvent polarity on the energetics of the investigated reactions, single-point energy calculations were performed with the polarizable continuum model (PCM) in EtOAc (*ε* = 5.98) on the gas-phase optimized geometries.

### Chemical models

In the interest of computational tractability, Rh_2_(OAc)_4_ was chosen as the model for dirhodiumtetracarboxylate complexes. Although experimentally it was not the most active catalyst for the C–H amination reaction with *N*-mesyloxycarbamates due to poor solubility, the catalyst provides the desired product with acceptable yields.^10^–^15^ Both intramolecular and intermolecular benzylic and aliphatic C–H insertion reactions were studied by DFT, with 2-phenylethyl *N*-mesyloxycarbamate (**A**) and *n*-hexyl *N*-mesyloxycarbamate (**B**) as the respective substrates (eqn (3) and (4)). The formation of the primary carbamate and the corresponding carbonyl compound was also studied, as these are known by-products identified namely in reactions with secondary *N*-mesyloxycarbamates (*vide infra*). (*R*)-Phenyl 2,2,2-trichloroethyl *N*-mesyloxycarbamate (**C**) is known to undergo C–H amination with 2-phenylethane and was selected to model the intermolecular pathway (eqn (5)).
3

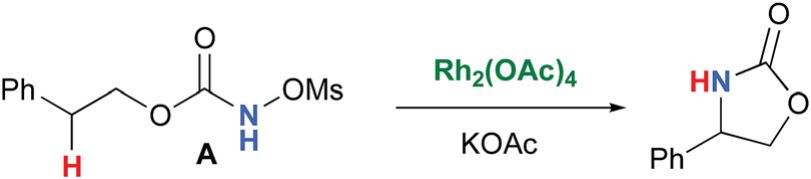



4

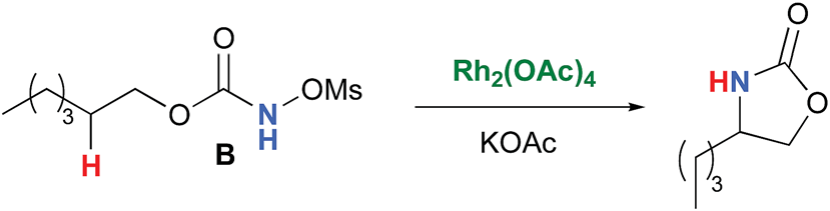



5

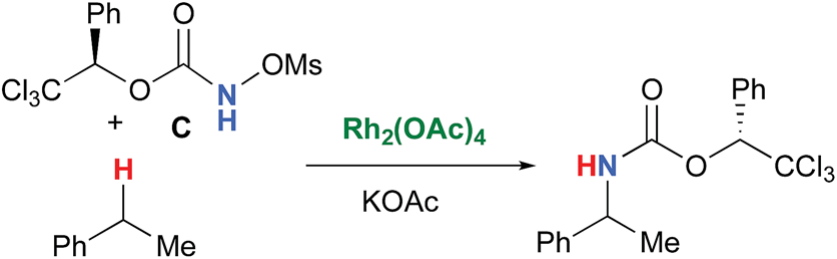




### Formation of the *N*-mesyloxycarbamate anion

**Scheme 3:**
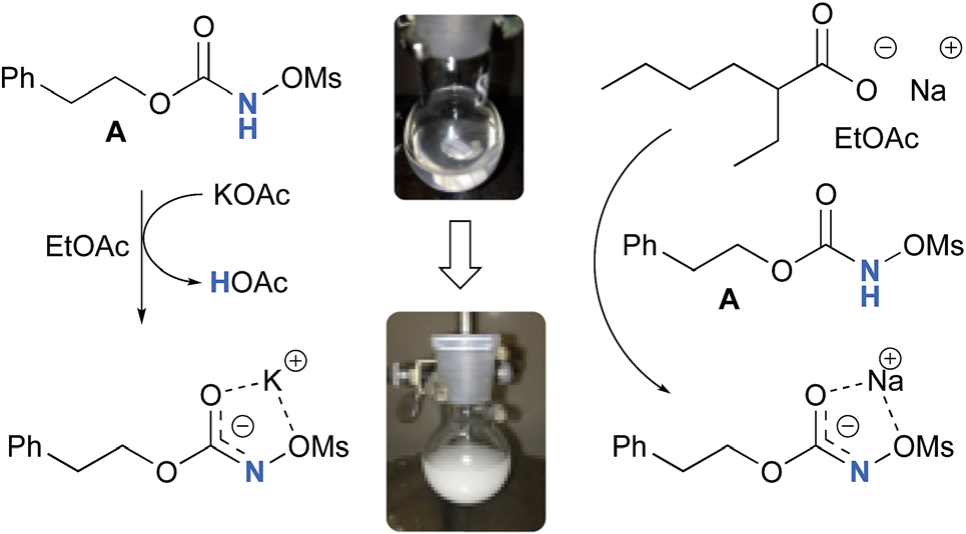
Formation of the potassium and sodium *N*-mesyloxycarbamate salt from **A**.

**Fig. 1:**
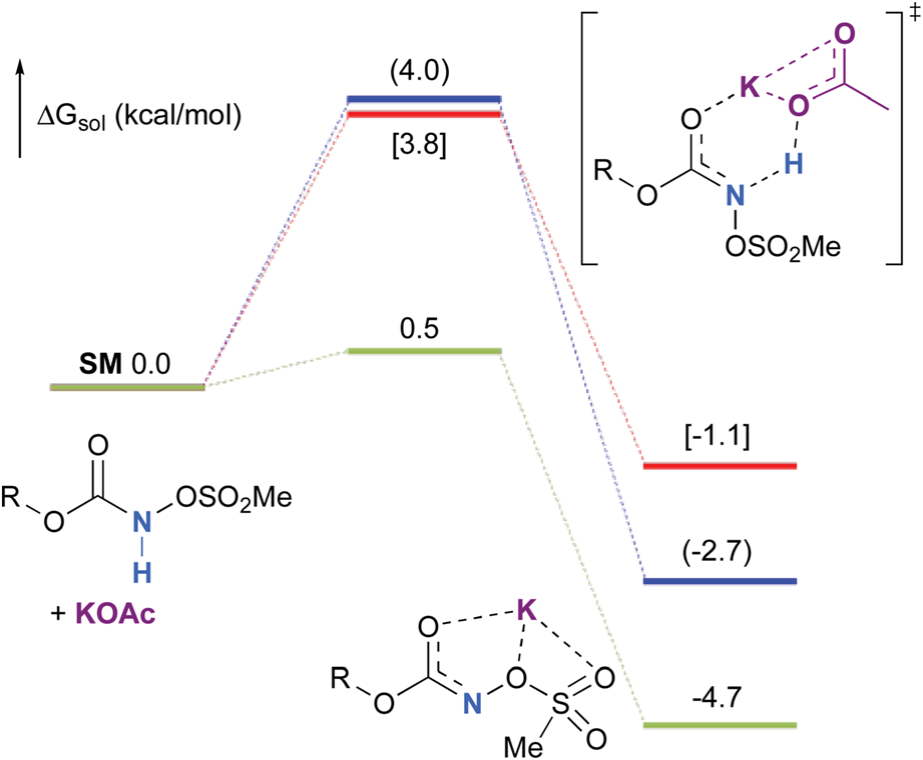
Calculated free energy profile for the formation of the potassium *N*-mesyloxycarbamate salt for **A** (in blue, values in parentheses), **B** (in red, values in brackets) and **C** (in green) at the PBE/BS2 level of theory (the relative free energies (Δ*G*_sol_, kcal mol^–1^) in ethyl acetate are provided).

**Fig. 2:**
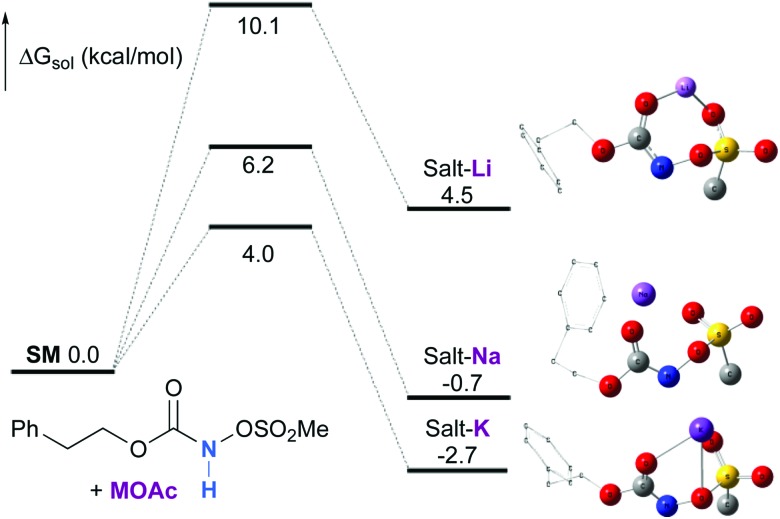
Calculated free energy profile for the formation of the potassium, sodium and lithium *N*-mesyloxycarbamate salts for **A** at the PBE/BS2 level of theory (the relative free energies (Δ*G*_sol_, kcal mol^–1^) in ethyl acetate are provided). (Different atom styles are used for clarity.)

Experimentally, the reaction between *N*-mesyloxycarbamate **A** and potassium acetate or sodium 2-ethylhexanoate (known to be soluble in organic solvents) produced the potassium or sodium *N*-mesyloxycarbamate salt as a white precipitate (Scheme 3).^12^ The rhodium catalyst was not necessary for salt generation to occur. When the deprotonation with potassium acetate was studied *in silico*, all three models afforded potassium *N*-mesyloxycarbamate salts (**Salt-K**) with a small energy barrier of 0.5 to 4.0 kcal mol^–1^ (Fig. 1). All potassium salts were more stable than the corresponding *N*-mesyloxycarbamate starting material. The inner anion is doubly stabilized by the potassium cation, where the negative charge is distributed over the N–C–O moiety. The N–H of *N*-mesyloxycarbamate **C** is the most acidic (due to the electron withdrawing Cl_3_C group), leading to the most favoured TS and stable **Salt-K**. Similar results were obtained with sodium acetate and **A** with slightly higher values for the deprotonation TS (6.2 kcal mol^–1^), affording the sodium salt (–0.7 kcal mol^–1^). Of note, it is known that lithium bases afford only trace quantities of the desired amination product, likely due to the poor solubility of these reagents. In addition, DFT calculations showed that the energy barrier for the deprotonation TS with lithium acetate and **A** was substantially higher than that with sodium acetate (10.1 *vs.* 6.2 kcal mol^–1^), confirming the experimental observations, a 7-membered ring coordinated lithium salt (+4.5 kcal mol^–1^) was observed (Fig. 2).

### Formation of the rhodium nitrene species

**Fig. 3:**
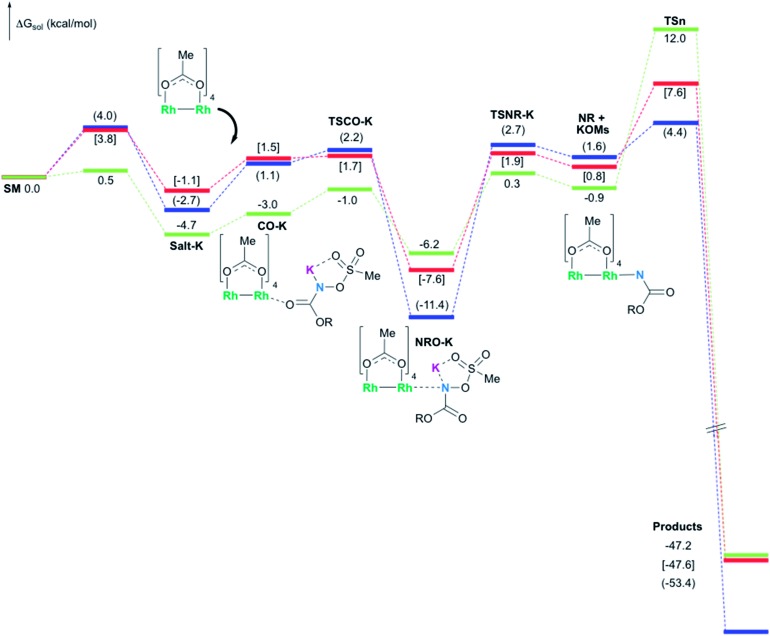
Calculated free energy profile for the formation of products (oxazolidinone for **A** (values in parentheses) and **B** (values in brackets), and aliphatic carbamate for **C**) at the PBE/BS2 level of theory (the relative free energies (Δ*G*_sol_, kcal mol^–1^) in ethyl acetate are provided).

**Fig. 4:**
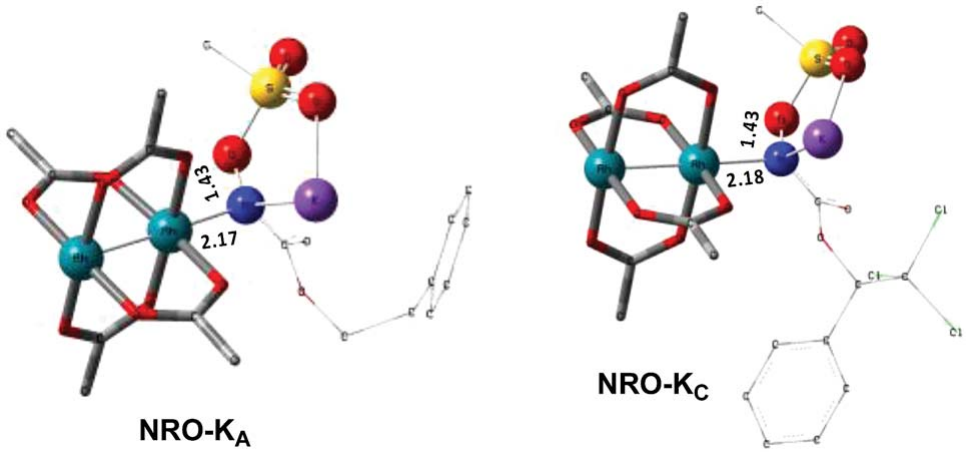
Optimized structures of the **NRO-K** of **A** and **C**. (Sulfur atom: yellow; oxygen atom: red; nitrogen atom: blue; rhodium atom: aqua.) Key bond distances (Å) are indicated. (Different atom styles are used for clarity.)

**Fig. 5:**
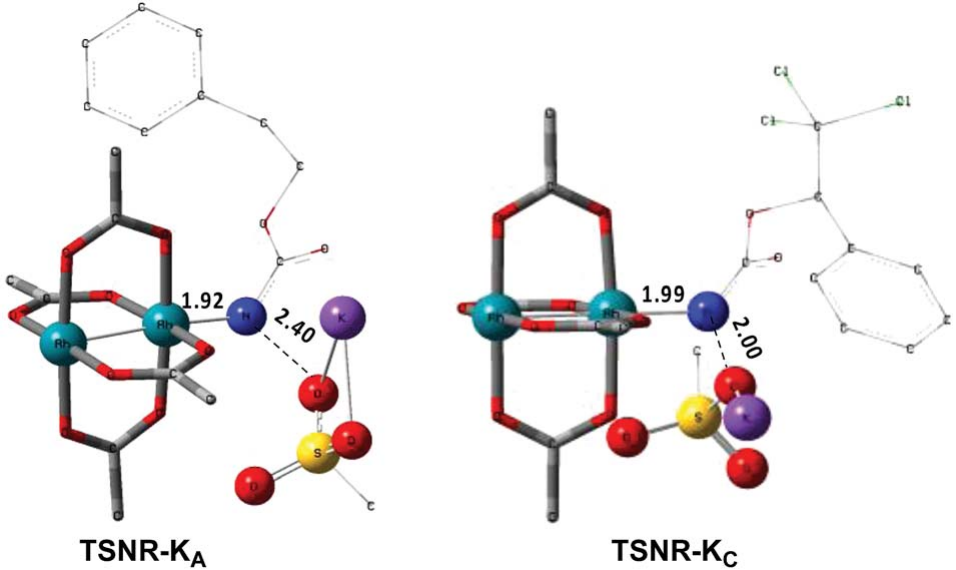
Optimized structures of the **TSNR** of **A** and **C**. (Sulfur atom: yellow; oxygen atom: red; nitrogen atom: blue; rhodium atom: aqua.) Key bond distances (Å) are indicated. (Different atom styles are used for clarity.)

In the presence of Rh_2_(OAc)_4_, the potassium *N*-mesyloxycarbamate salt first forms a coordination complex (**CO-K**), in which the carbonyl of the carbamate is coordinated to the apical position of the rhodium complex (Fig. 3).^83^ The same relative stability order between the three models as previously seen for **Salt-K** is observed, indicating that **CO-K** complexes resemble their corresponding free potassium anions (**Salt-K**). The **CO-K** complex affords the stable nitrenoid species (**NRO-K**) *via* the transition state **TSCO-K**, where both carbamate carbonyl and nitrogen are coordinated to rhodium. The calculated activation barrier is lower than the one for the deprotonation transition state. **NRO-K** is the most stable metal intermediate, displaying a strong interaction between the nitrogen atom and the rhodium center, with the leaving group (MsOK) still bound to nitrogen. The Rh–nitrogen bond length of **NRO-K** is calculated to be 2.17–2.18 Å (Fig. 4). Conformations in which Me(Ms) and unbound O(Ms) are interchanged were also explored, but no further stabilization was observed. Compared to the less basic **Salt-K_C_**, **Salt-K** derived from **A** and **B** displays a stronger coordination with the Lewis acid rhodium, affording more stable **NRO-K**. In addition, a cation π-interaction between the phenyl group and potassium appears to stabilize **NRO-K_A_** (Fig. 4). Such an interaction is not present in model **C**, probably because of the sterically hindered trichloromethyl group. As the most stable rhodium intermediate, rhodium nitrenoid species is likely the resting state of the catalyst in the catalytic cycle. Rhodium nitrenoid species subsequently undergoes an endergonic concerted elimination of the mesylate group affording reactive Rh–nitrene species (**NR**, Fig. 3). The departure of the mesylate ion is assisted by the counter anion (K), resulting in the stretching of the N–OMs bond and shortening of the N–Rh bond in the transition state (Fig. 5). All three rhodium nitrene species were computed to be higher in energy compared to their corresponding rhodium nitrenoid complexes. As a result, rhodium nitrenes are the most reactive intermediates, and may serve as the active species for delivery of the NR group. This is in contrast with previous reports, as the metal nitrene species were either as stable as the nitrenoid intermediate when using iminoiodinanes^59^ or more stable than the nitrenoid complex when using azide reagents.^69^,^76^,^84^ Rhodium nitrenes have three reactive centers localized in the Rh–Rh–N moiety as depicted in the analysis of frontier molecular orbitals of the singlet and triplet nitrene species (*vide infra*, Fig. 7). The endothermicity of the formation of rhodium nitrenes from rhodium nitrenoids (Δ*H*_r_ = +25.7, +22.6, and +21.3 kcal mol^–1^ for **NR_A_**, **NR_B_** and **NR_C_**, respectively) is due to the cleavage of the N–O(Ms) bond to release the mesylate salt. Consequently, the formation of **NR** from **NRO** is entropy driven and the positive Δ*G* value (+13.0, +8.4, and +5.3 kcal mol^–1^ for **NR_A_**, **NR_B_** and **NR_C_**, respectively) is a consequence of an increase of the enthalpy. Experimentally, we observed the precipitation of KOMs, which prevented the reversibility of the conversion of **NRO-K** to **NR**. The rhodium nitrene derived from *N*-mesyloxycarbamate **C** is the most stable, possibly due to a positive electronic effect of the trichloromethyl group.

**Fig. 6:**
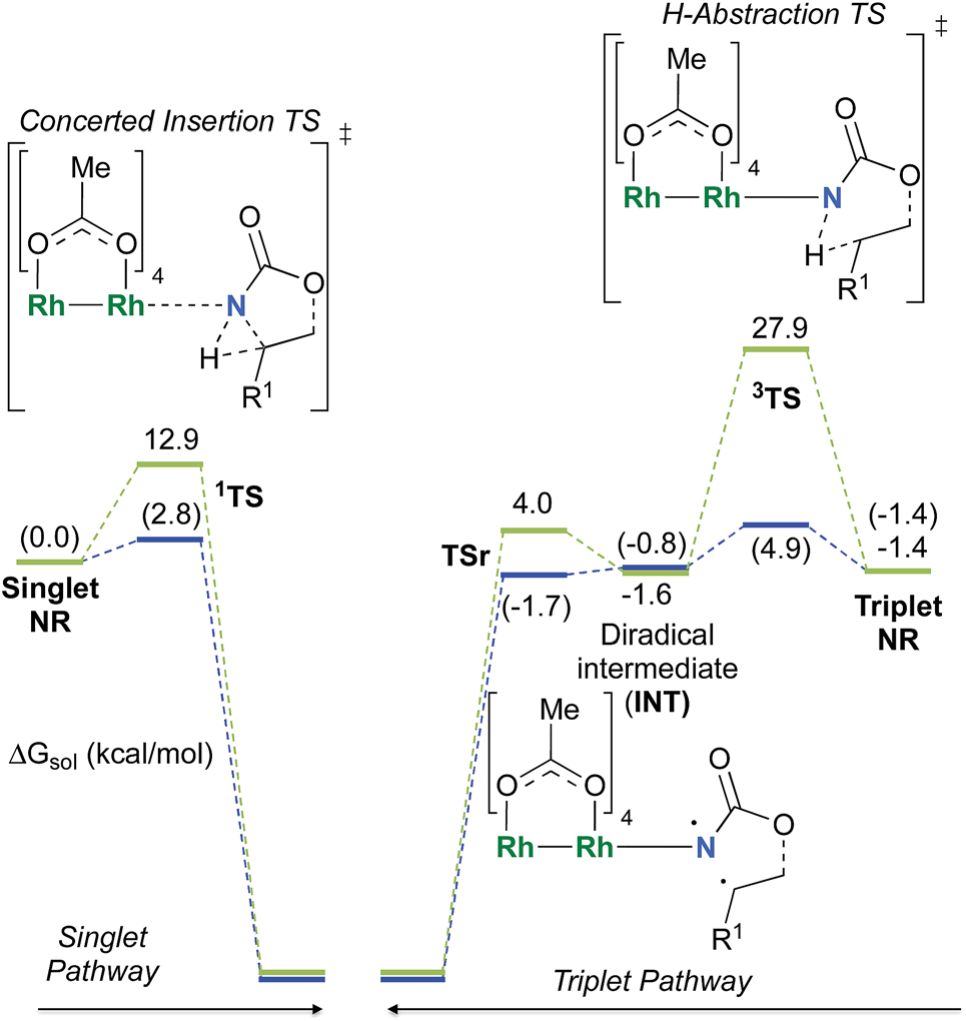
Calculated free energy profile for the singlet and triplet pathways with rhodium nitrene species of **A** (in blue, values in parentheses) and **C** (in green) at the PBE/BS2 level of theory (the relative free energies (Δ*G*_sol_, kcal mol^–1^) of ethyl acetate are provided).

**Fig. 7:**
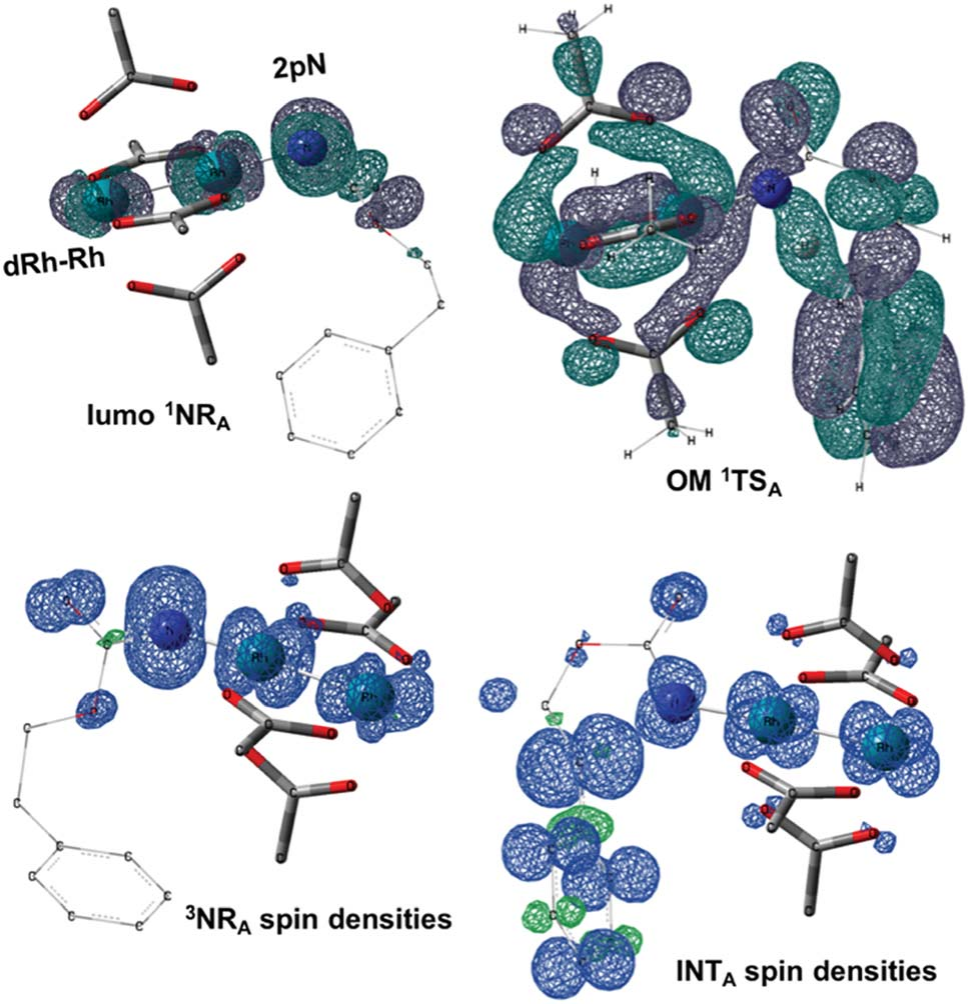
LUMO of the singlet nitrene species (LUMO **^1^NR_A_**), the density of the unpaired electrons of the triplet nitrene species (**^3^NR_A_** spin densities), the frontier orbital of **^1^TS_A_** (OM **^1^TS_A_**) and the spin densities of the diradical intermediate (**INT_A_**). (Different atom styles are used for clarity.)

### C–H insertion with triplet *vs.* singlet rhodium nitrene species

The relative thermodynamic stability of singlet (**^1^NR**) and triplet (**^3^NR**) rhodium nitrenes for all three models (**A**, **B** and **C**) has been calculated (Table 2). All values are within 1.0 kcal mol^–1^, indicating that both **^1^NR** and **^3^NR** could be present at similar concentrations. An additional electronic state that could be present is the open-shell singlet biradical. Despite attempts using the broken-symmetry DFT approach with a correction for triplet spin-contamination,^85^ these species could not be detected, only closed shell singlet states were observed.

**Table 2:** Δ*E*_st_ and Δ*G*_st_ for rhodium nitrenes of Models **A**, **B** and **C**^*a*^

PBE/BS2(PCM)	NR_A_	NR_B_	NR_C_
Δ*E*_st_ (kcal mol^–1^)	–0.8	–0.6	0.9
Δ*G*_st_ (kcal mol^–1^)	1.4	0.6	1.4

^*a*^ *E*_st_= *E*_singlet_ – *E*_triplet_.

As the **^1^NR** and **^3^NR** species are close in energy, it is necessary to explore the insertion of the rhodium nitrene species into the C–H bond through the two known competitive pathways (see Scheme 2). The reaction pathways were examined through the location of the transition states and the product complexes associated with **^1^NR** and **^3^NR** on the potential-energy surfaces for **A** and **C** (Fig. 6). For both intramolecular and intermolecular processes, the singlet pathways were favoured over the triplet pathways.^86^

**Fig. 8:**
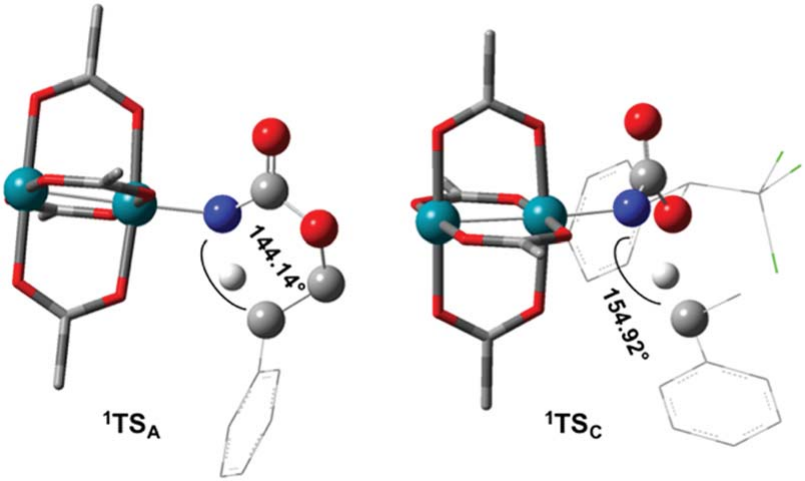
Optimized structures of the singlet C–H insertion TS for **A** and **C**. (Oxygen atom: red; nitrogen atom: blue; rhodium atom: aqua.) (Different atom styles are used for clarity.)

The reaction barrier for **^1^NR** insertion into a benzylic C–H bond is 2.1 kcal mol^–1^ and 15.0 kcal mol^–1^, respectively, lower than the corresponding triplet pathway for the intramolecular and intermolecular reactions. Upon examination of MO coefficients, the LUMOs of **^1^NR** have a large contribution from the nitrogen 2p orbital, making the N atom of **^1^NR** strongly electrophilic (Fig. 7). The LUMO extends mainly over the two rhodium atoms as well as nitrogen. The σ C–H bonding electrons are attracted by the vacant nitrogen 2p orbital; as a result, the total charge of the –CH_2_– moiety increases from 0.01 for **^1^NR_A_** to 0.10 for **^1^TS_A_** and from –0.00 for **^1^NR_C_** to 0.06 for **^1^TS_C_**, while the total charge of the –Rh_2_NCO- moiety decreases from 0.53 for **^1^NR_A_** to 0.43 for **^1^TS_A_** and from 0.55 for **^1^NR_C_** to 0.49 for **^1^TS_C_**, overall indicating a hydride transfer. The hydride transfer is accompanied by simultaneous formation of the C–N bond as revealed by the orbitals of **^1^TS** (Fig. 7). There is a strong orbital interaction between the carbon and nitrogen atoms, with a trigonal transition state structure, featuring a N–H–C angle of 144.14° for **^1^TS_A_** and a N–H–C angle of 154.92° for **^1^TS_C_**; as a result, both hydride transfer and C–N formation take place in a concerted manner (Fig. 8). Triplet nitrene species **A** (**^3^NR_A_**) has two singly occupied MOs and the two unpaired electrons are mainly localized on the Rh–Rh–N moiety (Fig. 7). The formation of the σ-N–H bond would require one electron from the approaching hydrogen atom to form a bond with an electron of a SOMO of the rhodium–nitrene complex, thus leading to a homolytic rather than a heterolytic cleavage of the C–H bond. The homolysis of the C–H bond leads to one unpaired electron residing on the C atom and the other residing in the remaining SOMO. In addition, the spin-density analysis supports these hypotheses.^87^ As the hydrogen atom approaches the nitrogen atom, the σ C–H bond undergoes a homolytic cleavage. As **^3^TS** proceeds to **INT**, the spin density of carbon increased to 0.705 (**A**) and 0.778 (**C**), and the configuration of the carbon atom changed from a pyramidal to a planar structure to produce **INT**. The result of the spin-density analysis shows that **INT** is a diradical (Fig. 7). As **INT** proceeds to the recombination transition state (**TSr**), the spin density of C decreases to 0.671 (**A**) and 0.766 (**C**). The configuration of the C atom returns to pyramidal with a shortening of the C–N interaction to produce **TSr**. The relative reaction rates for the singlet pathways over the triplet may be estimated according to the transition-state theory (eqn (6)).

6 *k*_singlet_/*k*_triplet_ ≈ exp[(Δ*G*_triplet_^‡^ – Δ*G*_singlet_^‡^ + Δ*G*_st_)]/*RT*

The relative reaction rates *k*_singlet_/*k*_triplet_ for the amidation of benzylic intramolecular and intermolecular C–H bonds are 33.7 and 8.4 × 10^10^, respectively. Consequently, it appears that the singlet-concerted pathway is predominant and responsible for the formation of oxazolidinone and carbamate products.

### Nitrogen-atom insertion into C–H bonds with singlet rhodium nitrene species

**Fig. 9:**
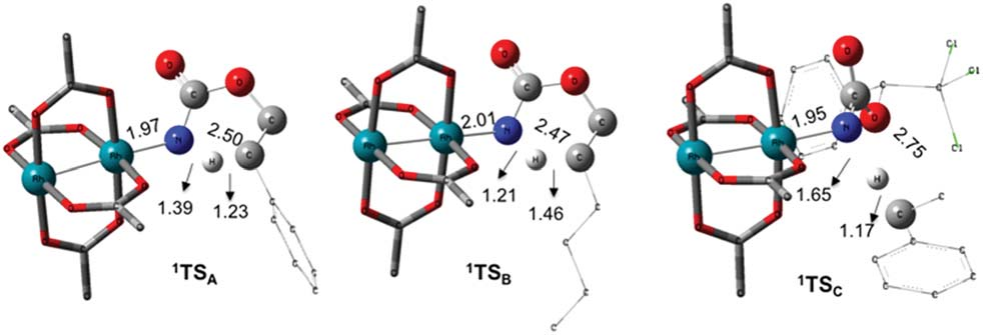
Optimized structures of the singlet C–H insertion TS for **A**, **B** and **C**. (Oxygen atom: red; nitrogen atom: blue; rhodium atom: aqua.) Key bond distances (Å) are indicated. (Different atom styles are used for clarity.)

**Scheme 4:**
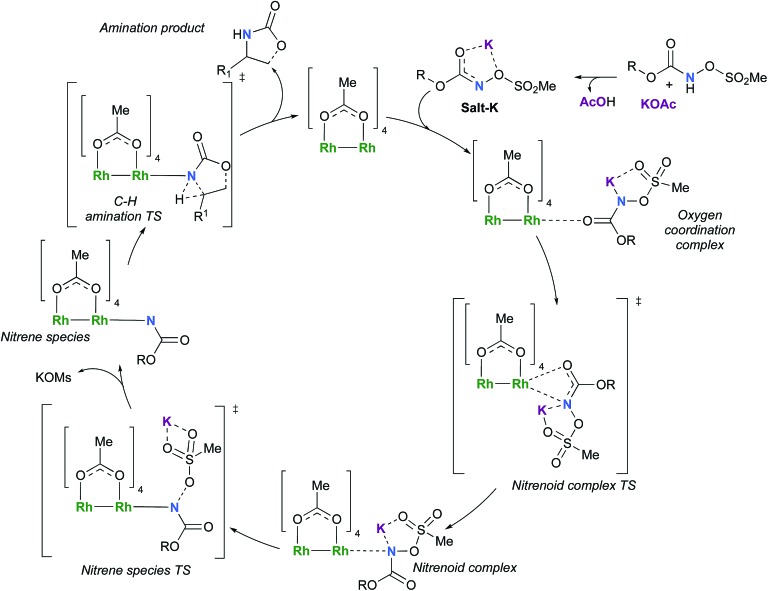
Proposed catalytic cycle for the rhodium-catalyzed C–H amination with *N*-mesyloxycarbamates.

The C–H insertion of the singlet rhodium nitrene was studied with all models (**A**, **B** & **C**) (Fig. 3). The intramolecular C–H bond insertions (**A** & **B**) are more facile than the intermolecular C–H bond insertion (**C**) by 7.6 and 4.4 kcal mol^–1^, respectively, as a result of a favourable entropy. **TS_C_** is however an early, thus accessible transition state (Fig. 9). Among the examined intramolecular C–H bond insertions, the **TSn** for the benzylic C–H bond insertion (**A**) was determined to be 3.2 kcal mol^–1^ lower than the **TSn** for the singlet aliphatic intramolecular C–H insertion (**B**), in agreement with experimental results reported. The structural analysis of the corresponding transition states shows that the Rh–N(R) and C–H bonds are 0.04 Å and 0.23 Å, respectively, longer in **TS_B_** than **TS_A_**, whereas (H)C–N and N–H(C) bonds are 0.03 Å and 0.18 Å shorter, respectively (Fig. 9). **TS_B_** is thus a late transition state with larger geometry differences from the starting material, suggesting a higher activation energy barrier compared to **TS_A_**. As mentioned earlier, for all three models, the rhodium nitrenoid complex (**NRO-K**) is the resting state of the catalyst, *i.e.* the turnover frequency (TOF)-determining intermediate (**TDI**). The energetic span model was used to assess the kinetics of the catalytic cycle.^88^–^90^ In addition to confirming **NRO-K** as the **TDI**, the transition state of the C–H insertion (**TSn**) was established as the TOF-determining transition state (**TDTS**) or the transition state involved in the rate-limiting step. An energy range of 15.8 and 18.2 kcal mol^–1^ (ΔΔ*G* = Δ*G***_TSn_** – Δ*G***_NRO_**), corresponding to the apparent activation energies of the full cycle, was determined for, respectively, the intramolecular amination of benzylic C–H bonds (**A**) and the intermolecular amination of C–H benzylic bonds (**C**). The experimental and calculated kinetic isotopic effect also confirms **TSn** as the rate limiting step (Table 3). The proposed catalytic cycle summarizing all mechanistic information is shown in Scheme 4.

**Table 3:** Calculated^91^ and experimental kinetic isotopic effect for the singlet and triplet pathways

Model	Concerted insertion KIE^*a*^	Radical insertion KIE^*a*^	Experimental KIE
**A**	1.60	10.8	1.53
**B**	—	—	2.31
**C**	3.77	6.31	3.60

^*a*^Quiver program was used to calculate KIE.

### Primary carbamates and carbonyl derivatives as by-products in intramolecular C–H amination reactions

With a few substrates, namely *N*-sulfonyloxycarbamates derived from secondary alcohols, the corresponding primary carbamate and carbonyl derivatives became the major products. For example, the reaction with *trans*-4-phenylcyclohexyl-*N*-tosyloxycarbamate **D** afforded the ketone and the corresponding primary carbamate in respectively 58% and 15% yields (eqn (7)). The formation of ketones has also been observed in rhodium-catalyzed C–H amination reactions using iminoiodinanes as metal nitrene precursors.^92^–^95^ The formation of this by-product was postulated to be the result of an α-C–H insertion reaction producing a strained 4-membered intermediate that decomposes to afford the observed ketone.^92^ No experimental or *in silico* data are available to support this hypothesis. With orthohalogenated *N*-mesyloxycarbamates such as substrate **E**, only the corresponding primary carbamate was isolated (eqn (8)). Primary carbamates have been observed in other catalyzed C–H amination reactions. Initially, it was postulated that they arose from the homolytic cleavage of the [M]–N bond, as they appeared only when the metal–nitrene species are not rapidly intercepted by the substrate.^33^ However, DFT calculations involving porphyrin–cobalt-catalyzed C–H amination reactions using azides as metal nitrene precursors revealed that primary carbamates were formed *via* hydrogen atom abstraction processes from the substrate.^72^
7

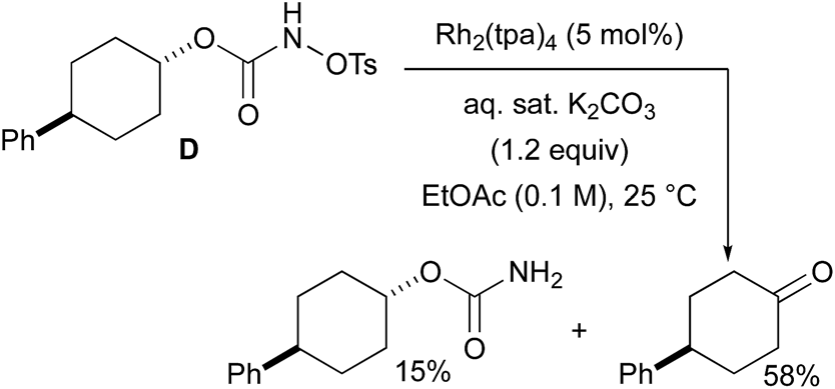



8

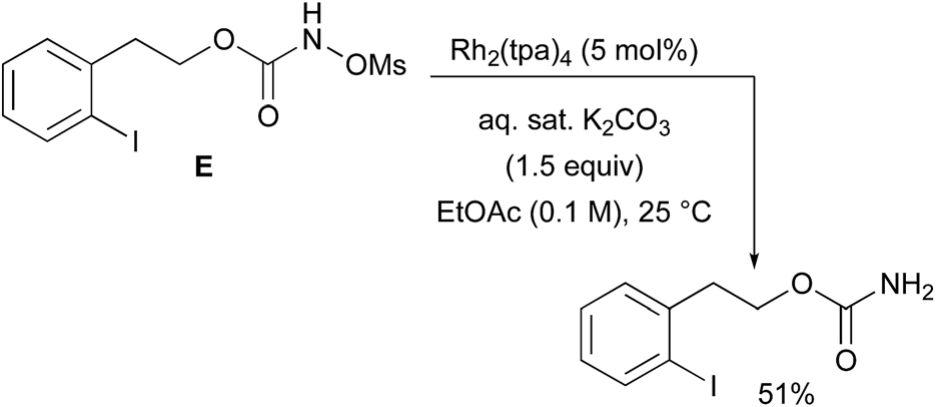




**Scheme 5:**
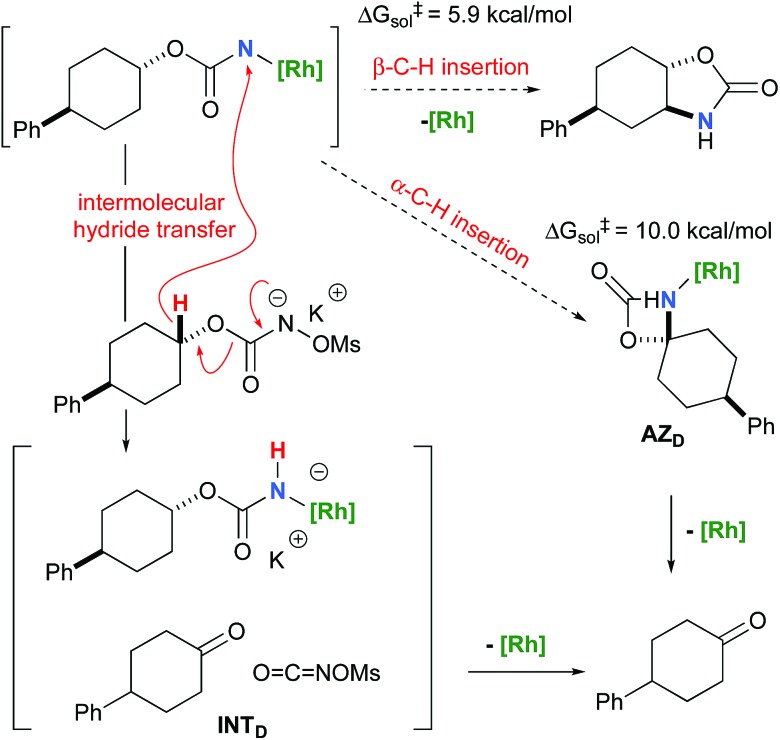
Ketone *vs.*oxazolidinone formation from substrate **D**.

To further investigate the origin of the side reactions in rhodium-catalyzed aminations, we considered the formation of the ketone and primary carbamate as by-products from **A**, but also from substrates **D**&**E**.^96^ We first ruled out a number of hypotheses, including competitive reactivity of the triplet Rh–nitrene species (namely homolytic cleavage of the [M]–N bond) and α-intramolecular C–H amination, as those were found to have inaccessible activation energies at room temperature. For instance, the β-C–H amination **^1^TS_D_** is 4.1 kcal mol^–1^ more favourable than the α-C–H amination **^1^TS_D_**, leading to the formation of the corresponding ketone (Scheme 5). Conversely, we ascertained that a bimolecular α-hydride transfer from **Salt-K** to the electrophilic singlet Rh–nitrene species could compete with an intramolecular β-C–H insertion process.

**Fig. 10:**
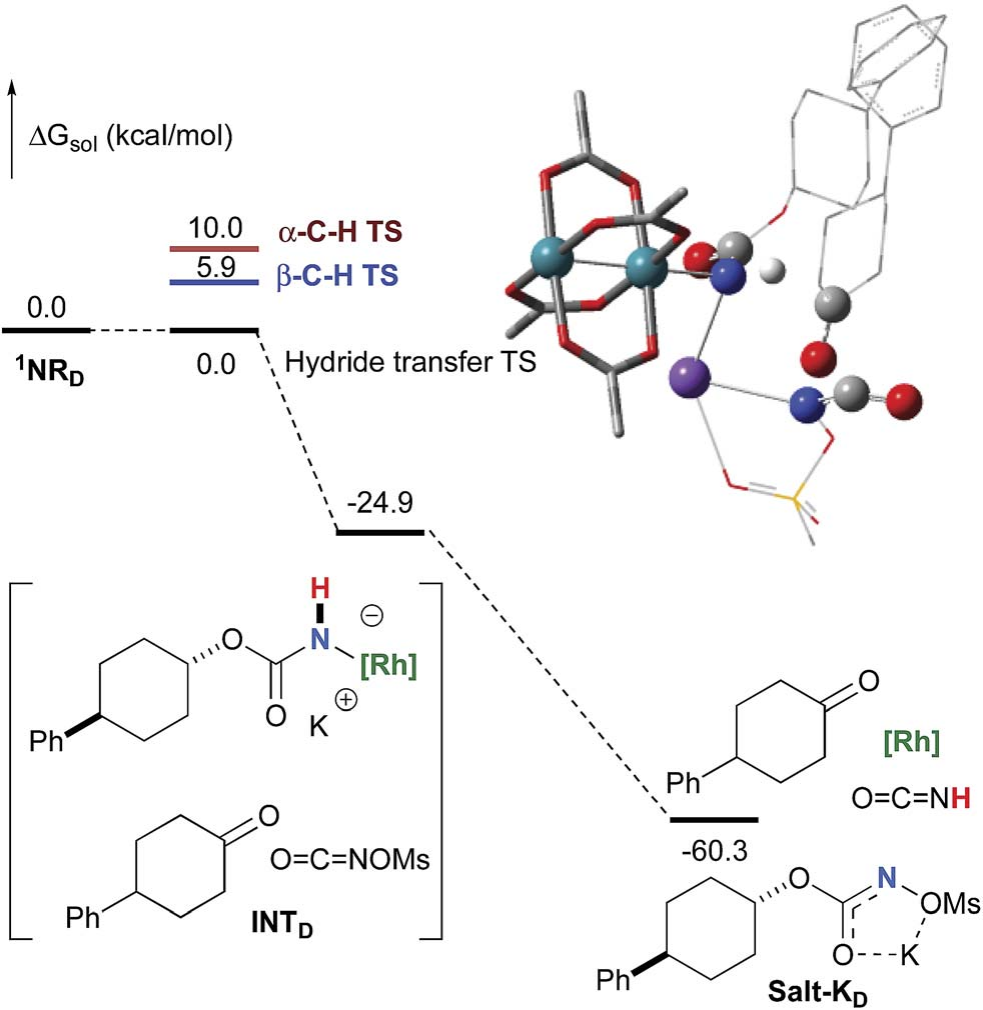
Calculated free energy profile for the formation of the ketone from **D** at the PBE/BS2 level of theory (the relative free energies (Δ*G*_sol_, kcal mol^–1^) in ethyl acetate are provided). (Different atom styles are used for clarity.)

In addition to the ketone, a rhodium imido species is also formed that is either protonated to afford the corresponding primary carbamate or reacted with O=C=NOMs to regenerate **Salt-K_D_**.^97^ For substrate **D**, this pathway displays an overall free activation barrier (Fig. 10). Steric hindrance plays a role in decelerating the intramolecular amination of substrate **D**. The transition state for the hydride bimolecular transfer has an intramolecular character with the potassium cation coordinating the two interacting molecules favouring the formation of the ketone by-product. The side-reaction is also enthalpy-driven, considering the high stability of the produced ketone.

**Fig. 11:**
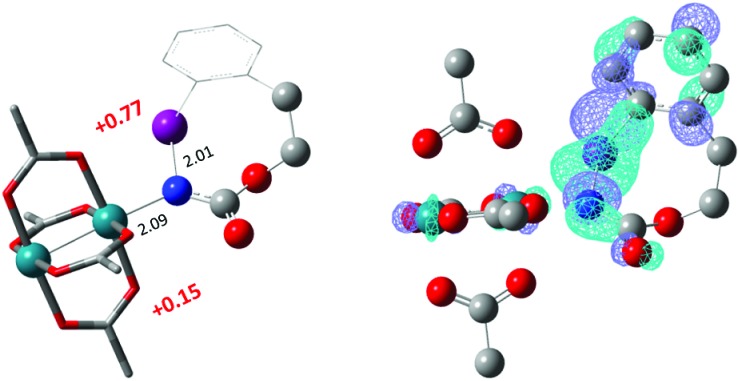
Structure of the singlet rhodium nitrene species derived from *N*-mesyloxycarbamate **E** showing the coordination between the iodine and the nitrogen, the distribution of partial charges by natural bond orbital analysis (in red) and the localized LUMO on the iodine. (Oxygen atom: red; nitrogen atom: blue; rhodium atom: aqua; iodide atom: purple.) Key bond distances (Å) are indicated. (Different atom styles are used for clarity.)

**Fig. 12:**
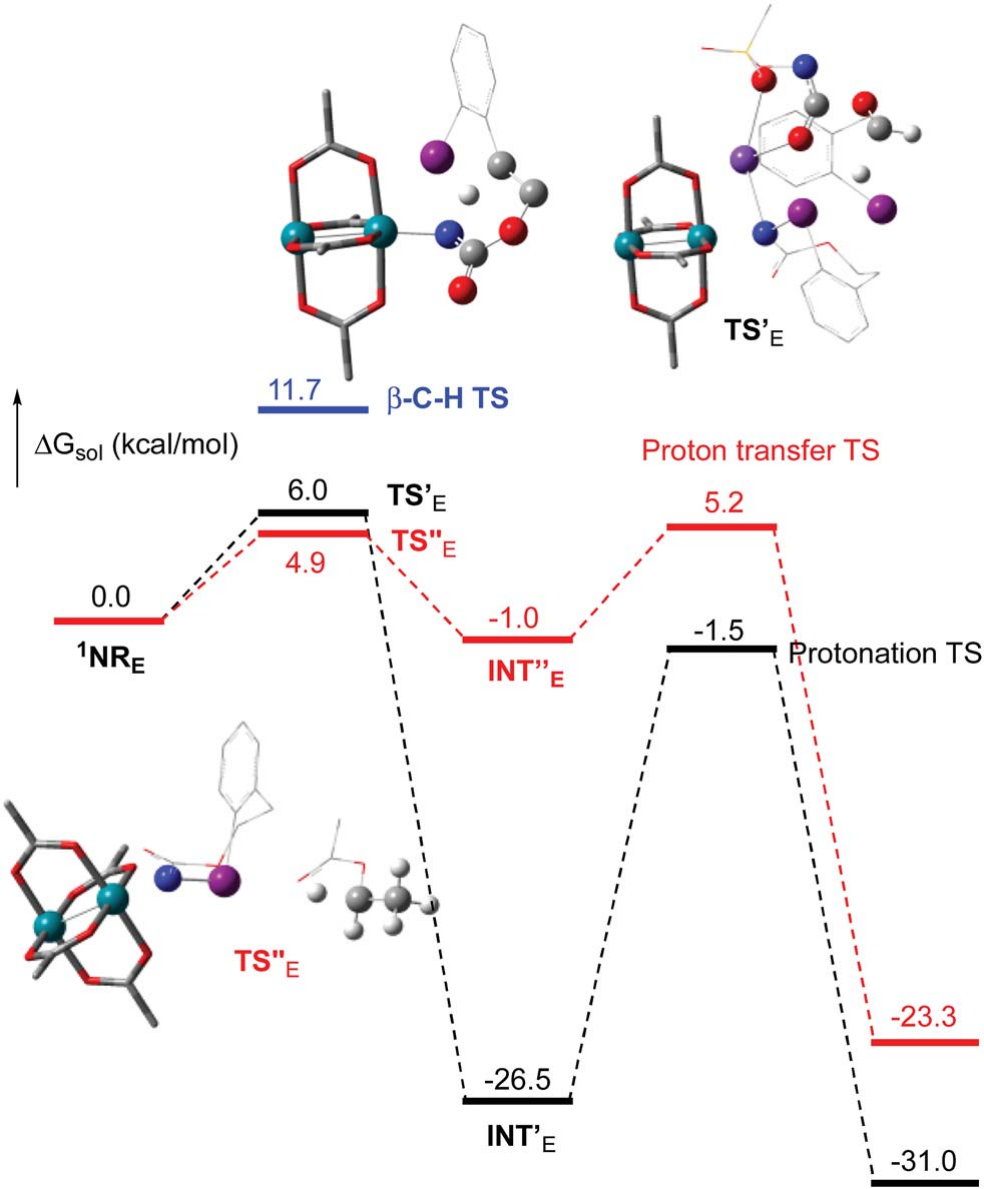
Calculated free energy profile for the reactivity of **NR_E_** at the PBE/BS2 level of theory (the relative free energies (Δ*G*_sol_, kcal mol^–1^) in ethyl acetate are provided). (Different atom styles are used for clarity.)

**Scheme 6:**
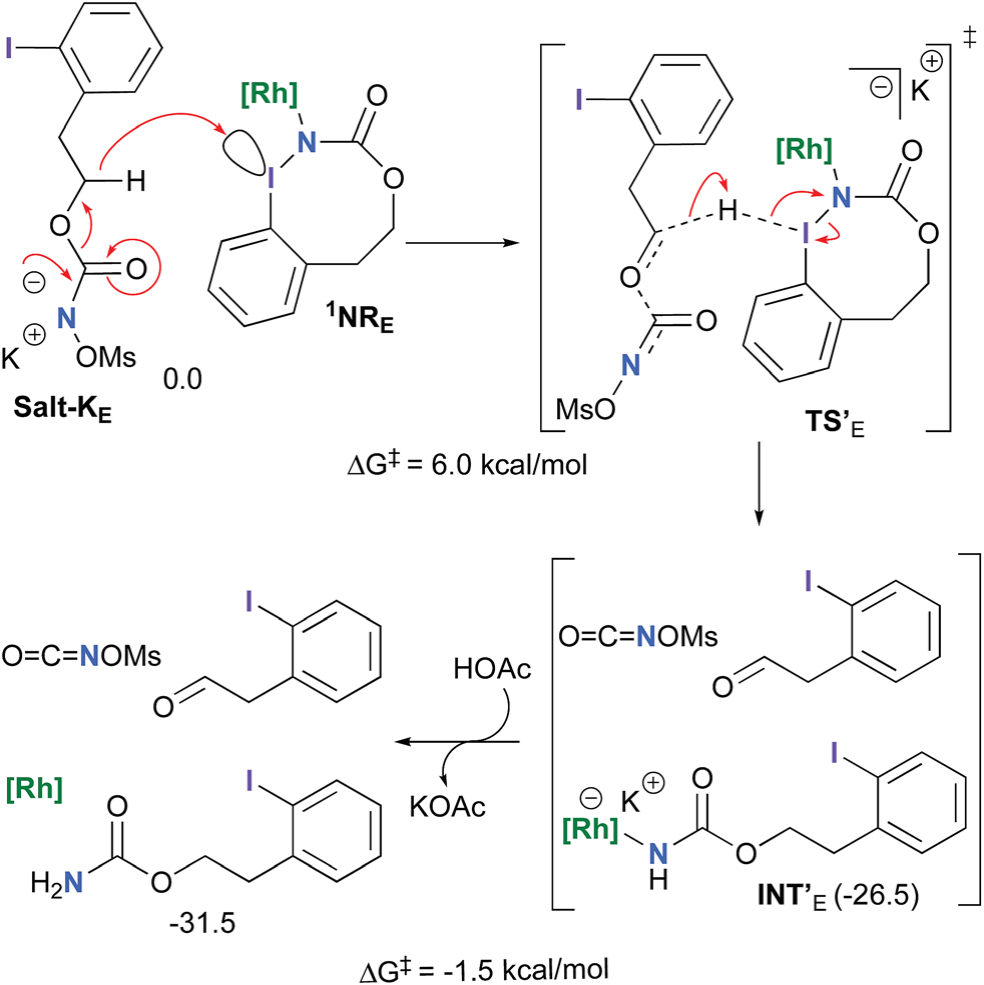
Hydride transfer from **Salt-K_E_** to **^1^NR_E_**.

The hydride transfer hypothesis between **Salt-K** and **^1^NR** cannot account for the reactivity of substrate **E** which formed exclusively the corresponding primary carbamate with no ketone. When we computed the rhodium nitrene derived from substrate **E**, we found a cyclic species, in which the iodine is coordinated to the nitrogen of the rhodium nitrene, with the N–I bond (2.01 Å) shorter than the Rh–N bond (2.09 Å) (Fig. 11). Donation from the p-halogen (5p–I) to the vacant 2p orbital of nitrogen is observed at the LUMO of the Rh–nitrene species **E**. This frontier orbital is thus mostly localized on the iodine resulting in a less electrophilic nitrogen. The charge analysis shows that the Rh_2_NCO moiety has a total charge of +0.15, whereas the iodine atom is highly electrophilic with a partial charge of +0.77. Because of a crowded iodine intra-coordinated **^1^NR_E_** with a less electrophilic nitrogen, the calculated intramolecular β-C–H amination energy barrier is significantly higher (8.9 kcal mol^–1^) than the one calculated for **A** (Fig. 12). It was also discovered that hydride transfer processes proceed *via* the Rh–Rh–N–I π* orbital of iodine instead of the nitrogen. A barrier of 6.0 kcal mol^–1^ (**TS′_E_**) was calculated for a hydride transfer from **Salt-K_E_**, forming **INT′_E_** (Scheme 6).

**Scheme 7:**
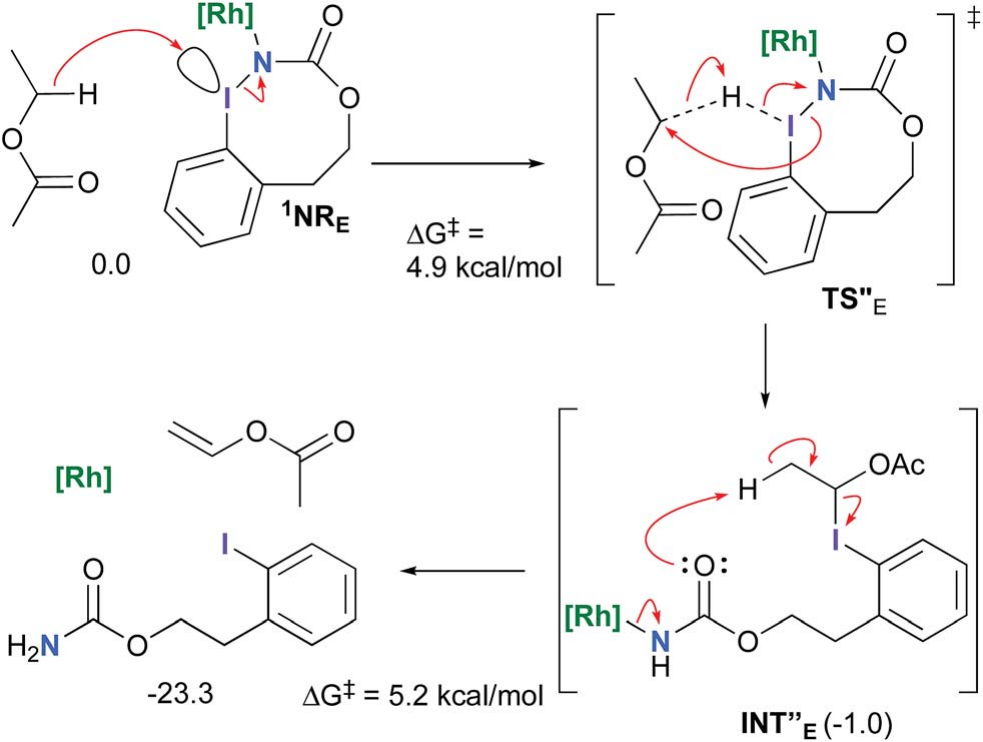
Hydride transfer from ethyl acetate to **^1^NR_E_**.

However, if such a process is operative, the aldehyde would have been isolated experimentally. Ethyl acetate (solvent) was then suspected as the hydride source. The transfer to the **^1^NR_E_** potential energy surface revealed an energy barrier of only +4.9 kcal mol^–1^ leading to a less stable imido intermediate **INT′′_E_** with a new C–I (C···I = 2.22 Å) bond formed (Scheme 7). The proton transfer is performed *via* the oxygen of the carbamate moiety simultaneously with the C–I cleavage (C···I = 2.67 Å) and the C=C formation of the alkene.

Proton tautomerization affords the primary carbamate. The overall Δ*G*_sol_ for the formation of the primary carbamate from **^1^NR_E_** and EtOAc is –23.3 kcal mol^–1^. When compared to the hydride transfer pathway with **Salt-K_E_**, the kinetic process is 3.8× faster with EtOAc. Given that EtOAc is also the solvent of the reaction, the pathway shown in Scheme 7 appears as the most plausible explanation to account for the exclusive isolation of primary carbamate **4** (with no aldehyde) experimentally.

## Conclusions

A computational study of the rhodium-catalyzed C–H amination with *N*-mesyloxycarbamates was presented, where we consider the concerted C–H amination from a closed shell singlet Rh–nitrene species and a stepwise radical mechanism starting from an open-shell triplet Rh–nitrene species. The overall reaction is favoured by ≈–50.0 kcal mol^–1^. The coordination of the anion of the *N*-mesyloxycarbamate with the catalyst takes place in an associative way, chelating the carbonyl function of the substrate to the catalyst. Upon coordination, the O–Rh bond is substituted by the N–Rh bond through a low activation barrier. With the inner nitrenoid complex identified as the TOF-determining intermediate, potassium plays a relevant role as the coordinative cation holding together the leaving group and the nitrene moiety giving rise to a stable nitrenoid complex. The potassium mesylate leaves the imido-complex through a late transition state where the barrier is reachable at room temperature. After the loss of the salt, the two spin states of nitrene species (singlet and triplet) are both capable of performing the C–H insertion through two competitive paths. A concerted insertion through a singlet rhodium–nitrene species is computed to have a smaller activation barrier, rather than a H-abstraction from the triplet nitrene species, affording the amine product and releasing the catalyst. Along with the kinetic isotopic effect, this computational study has clearly identified the C–H insertion transition state as the one involved in the rate-limiting step. The rhodium catalyst is qualified as a more efficient catalyst for the C–H intramolecular amination reaction under these conditions with a calculated TOF value of 2.3 × 10^2^ h^–1^ compared to a calculated TOF value of 21 h^–1^ for the analogous intermolecular C–H amination.

Furthermore, the DFT study has ruled out homolytic cleavage of the [M]–N bond and α-intramolecular C–H amination as pathways responsible for the formation of ketones and primary carbamates as by-products. There is instead a competitive intermolecular reaction of the singlet rhodium nitrene species with a hydride source (either the *N*-sulfonyloxycarbamate anion or ethyl acetate) affording the primary carbamate with or without the corresponding carbonyl by-product. Now that the pathway leading to their formation has been established, one can envision that the control of the reaction conditions (more diluted reaction conditions, solvent change) may in some cases minimize the formation of these undesired products. Given that ketones had also been identified as by-products in other rhodium-catalyzed nitrene C–H insertions,^92^–^95^ namely with iminoiodinanes, the identified pathway may also be operational in these systems (with the iminoiodinane as the hydride source). Finally, we also established that iodine, through intramolecular coordination of the rhodium nitrene, may in some substrates strongly impede the electrophilic nature of the nitrogen.

## Conflicts of interest

There are no conflicts to declare.

## Supplementary Material

Supplementary information
