# Supplementary material for: Rhodium(ii)-catalyzed C–H aminations using N-mesyloxycarbamates: reaction pathway and by-product formation
Source: Chem Sci. 2018 Oct 22;10(3):718–29. doi: 10.1039/c8sc03153c (PMC6340404; doi:10.1039/c8sc03153c)
Supplement: Supplementary file 1 [file SC-010-C8SC03153C-s001.pdf]

Electronic Supplementary Material (ESI) for Chemical Science.
This journal is © The Royal Society of Chemistry 2018

Supporting information

Rhodium(II)-Catalyzed C–H Aminations using *N*-Mesyloxycarbamates: Reaction Pathway and By-Product Formation

Emna Azek, Maroua Khalifa, Johan Bartholoméüs, Matthias Ernzerhof and Hélène Lebel

Département de Chimie, Université de Montréal, C.P. 6128,

Succursale Centre-ville, Montréal, Québec, Canada H3C 3J7.

helene.lebel@umontreal.ca

Table of Content

S1. Computational Methods.. S4

S2. Tables, Schemes and Figures...S5

Table S2-1. The Singlet and Triplet Energy Split (E_{st}=$E_{singlet}$-$E_{triplet}$) in kcal/mol of the $((OAc)_4Rh_2$=NH Complex with Different Computational Methods and Basis-Set Levels.... S5

Table S2-2. Calculated Free Energy Profile for the Singlet and Triplet Pathways with Rhodium Nitrene Species of A at the B3LYP/BS2 Level of theory (the relative free energies (ΔG_{sol}, kcal/mol in ethyl acetate are provided)... S5

Table S2-3. Spin Densities for Selected Atoms in **^{3}NR**, **^{3}TS**, **^{3}INT** and **TSr** for **A** and **C** at the PBE/BS1 Level of Theory.. S5

Table S2-4. Calculated Activation Barrier for the Reaction of **^3NR_A** with Various Hydrogen Sources at the PBE/BS2 Level of Theory (ΔG_{sol}, kcal/mol in ethyl acetate)...................... S6

Table S2-5. Calculated Activation Barrier for the Reaction of **^3NR_E** with Various Hydrogen Sources at the PBE/BS2 Level of Theory (ΔG_{sol}, kcal/mol in ethyl acetate)........................ S6

Scheme S2-1. Calculated Activation Barrier for the α-C-H insertion for **^1NR_A** at the PBE/BS2 Level of Theory (ΔG_{sol}, kcal/mol in ethyl acetate).. S6

Scheme S2-2. Intermolecular Hydride transfer from **Salt-K_A** to **^1NR_A** affording 2-phenyl-acetaldehyde... S7

Figure S2-1. Calculated Free Energy Profile of the Formation of 2-Phenylacetaldehyde from **^1NR_A** at the PBE/BS2 Level of theory.. S7

Figure S2-2. Rate Comparison for the C-H vs C-D Amination with *N*-Mesyloxycarbamate **A**.. S8

Figure S2-3. Rate Comparison for the C-H vs C-D Amination with *N*-Mesyloxycarbamate **B**... S8

Figure S2-4. Rate Comparison for the Amination of Ethyl Benzene and Deuterated Ethyl Benzene with *N*-Mesyloxycarbamate **C** S9

S3. Procedures for the kinetic isotope effect study.. S9

S4. Synthesis of the primary carbamate from 2-iodophenetyl-*N*-mesyloxycarbamate........... S11

S5. Cartesian coordinates, total energies (a.u), vibrational zero-point energies (a.u) free energies and solvation free enthalpies (a.u, at 298.15 K, and 1 atm) for the stationary structures.. S12

S5-1. Deprotonation step of **A**, **B** & **C** with KOAc (Figure 1)... S12

S5-2. Deprotonation step of **A** with NaOAc (Figure 2)...S18

S5-3. Deprotonation step of **A** with LiOAc (Figure 2)...S19

S5-4. Rhodium-Nitrene species formation of **A**, **B** & **C** with KOAc (Figure 3)………………… S21

S5-5. Triplet Rh-Nitrenes species of **A**, **B** & **C** (Table 2)…………………………………………………. S35

S5-6. Rh-Nitrenes C-H insertion mechanisms for **A** & **C** (Figure 6)……………………………… S37

S5-7. Rh-Nitrenes C-H insertion mechanisms for **B** (Figure 3)……………………………………..S46

S5-8. Ketone Formation from **D** (Figure 10)…………………………………………………………………..S48

S5-9. By-products formation for Substrate **E** (Figure 12)……………………………………………… S56

S5-10. Hydrogen transfer to ^{3}**NR**$_{A}$ (Table S2-3)……………………………………………………………. S68

S5-11. Hydrogen transfer to ^{3}**NR**$_{E}$ (Table S2-4)……………………………………………………………… S73

S5-12. By-products formation for Substrate **A** (Scheme S2-1, S2-2 & Figure S2-1)…………S78

S1. Computational Methods

The Gaussian '09 software package[1] was used for all calculations reported in this paper. Reaction and activation energies were calculated using Kohn-Sham density functional theory (DFT) with the PBE approximation for the exchange-correlation energy. Geometry optimization, harmonic vibrational frequency calculations, intrinsic reaction coordinate (IRC) calculations, Kohn-Sham orbital analysis, and Mulliken spin-density analysis were carried out in the gas phase with the 6-31G(d) basis set for H, C, N, O, S, Cl, K, Na and Li atoms, LANL2DZ augmented with p and d functions for I and the 1997 Stuttgart relativistic small-core potential (Stuttgart RSC 1997 ECP)[2] for Rh, augmented with a 4f function (**ζ**f(Rh)=1.350).[3] This composite basis set (denoted as BS1) was found to be effective for the assessment of activation free energies of Rh-centered complexes. Heavy-atom basis set definitions and corresponding pseudopotential parameters were obtained from the EMSL basis set exchange library. Energetics of the reported structures (PBE/BS1 optimized geometries) were improved by performing single-point energy calculations at the PBE level of theory in conjunction with the 6- 311++G(d,p) set for C, H, N, O, Cl, K, Li, Na and S and the same basis set as in BS1 for the I and Rh atoms (denoted as BS2). Free energies are reported in kcal/mol and were calculated at 1 atm and 298.15 K. Solvent effects in ethyl acetate were included by means of the PCM method.[4] In these calculations, the free energy of solvation was computed as $G_{solv} = E_{solv} + \Delta G_{corr\_gas}$. E_{solv} refers to the solvation single point energy and ΔG_{corr_gas} refers to the thermal correction to the free energy of the solute in the gas phase. The charge analysis has been performed by the natural bond orbital method[5] at PBE/BS2 level of theory using natural bond orbital (NBO) program under Gaussian 09 program package. According to the calculated energy Hessians, the stationary points, minima or transition states, are defined by having 0 and 1 imaginary frequency, respectively. Graphical analysis of the imaginary vibrational normal modes as well as the performed IRC calculations confirmed the nature of the located transition states. Explicit relativistic effects treatments were performed using LANL2DZ basis set augmented with p and f functions for Rh denoted as BS3[6]

Cartesian coordinates, total energies (a.u), vibrational zero-point energies (a.u) and free energies (a.u, at 298.15 K, and 1 atm) for the stationary structures Rh-nitrene species, amination TS for substrate **A**, **B** and **C** as well as by-products TS formation for substrates **A**, **D** and **E** are provided. The order of the stationary point is based on the number of imaginary frequencies.

---

[1] M. J. Frisch, G. W. Trucks, H. B. Schlegel, G. E. Scuseria, M. A. Robb, J. R. Cheeseman, G. Scalmani, V. Barone, B. Mennucci, G. A. Petersson, H. Nakatsuji, M. Caricato, X. Li, H. P. Hratchian, A. F. Izmaylov, J. Bloino, G. Zheng, J. L. Sonnenberg, M. Hada, M. Ehara, K. Toyota, R. Fukuda, J. Hasegawa, M. Ishida, T. Nakajima, Y. Honda, O. Kitao, H. Nakai, T. Vreven, J. A. Montgomery Jr., J. E. Peralta, F. Ogliaro, M. Bearpark, J. J. Heyd, E. Brothers, K. N. Kudin, V. N. Staroverov, R. Kobayashi, J. Normand, K. Raghavachari, A. Rendell, J. C. Burant, S. S. Iyengar, J. Tomasi, M. Cossi, N. Rega, J. M. Millam, M. Klene, J. E. Knox, J. B. Cross, V. Bakken, C. Adamo, J. Jaramillo, R. Gomperts, R. E. Stratmann, O. Yazyev, A. J. Austin, R. Cammi, C. Pomelli, J. W. Ochterski, R. L. Martin, K. Morokuma, V. G. Zakrzewski, G. A. Voth, P. Salvador, J. J. Dannenberg, S. Dapprich, A. D. Daniels, Ö. Farkas, J. B. Foresman, J. V. Ortiz, J. Cioslowski, D. J. Fox, Gaussian 09, revision A.01; Gaussian, Inc.: Wallingford, CT, 2009.

[2] (a) U. Steinbrenner, A. Bergner, M. Dolg and H. Stoll, *Mol. Phys.*, 1994, **82**, 3. (b) A. Henglein, *J. Phys. Chem.*, 1993, **97**, 5457. (c) M. Kaupp, P. v. R. Schleyer, H. Stoll and H. Preuss, *J. Chem. Phys.*, 1991, **94**, 1360.

[3] W. H. Lam, K. C. Lam, Z. Lin, S. Shimada, R. N. Perutz and T. B. Marder, *Dalton Trans.*, 2004, 1556.

[4] J. Tomasi, B. Mennucci and R. Cammi, *Chem. Rev.*, 2005, **105**, 2999.

[5] A. E. Reed, L. A. Curtiss and F. Weinhold, *Chem. Rev.*, 1988, **88**, 899.

[6] O. M. Roscioni, E. P. Lee, J. M. Dyke, *J. Comput. Chem.*, 2012, **33**, 2049.

S2. Tables, Schemes & Figures

Table S2-1. The Singlet and Triplet Energy Split ($E_{st}=E_{singlet}-E_{triplet}$) in kcal/mol of the $((OAc)_4Rh_2{=}NH$ Complex with Different Computational Methods and Basis-Set Levels.

Model	LDA/ BS1	PBE /BS1	PBE-DKH[7]/BS3	BPW91/ BS[8]	PBEh/ BS1	PBEh-DKH[7]/BS3	B3PW91 /BS[8]	B3PW91-DKH[7]/BS3	B3LYP /BS1	B3LYP-DKH[7]/BS	CCSD(T)// BPW91[8]
ΔE_{st}	-1.7	1.2	0.8	2.9	13.2	12.1	13.2	10.9	10.8	10.4	1.4

Table S2-2. Calculated Free Energy Profile for the Singlet and Triplet Pathways with Rhodium Nitrene Species of A at the B3LYP/BS2 Level of theory (the relative free energies (ΔG_{sol}, kcal/mol in ethyl acetate are provided).

B3LYP/BS2	Singlet pathway	Triplet pathway
NR_A	0.0	-6.3
TS_A	5.5	6.2
$^3INT_A$	---	-13.1
$\Delta\Delta G^{‡}$	0.7	
$k_{singlet}/k_{triplet}$	3.2	

Table S2-3. Spin Densities for Selected Atoms in **^{3}NR**, **^{3}TS**, **^{3}INT** and **TSr** for **A** and **C** at the PBE/BS1 Level of Theory.

	Rh_2	N	C	Total
^3NR_A	0.840	1.104	0.008	1.952
^3TS_A	0.816	0.827	0.418	2.061
$^3INT_A$	0.852	0.280	0.705	1.837
TSr_A	0.923	0.254	0.671	1,857
^3NR_C	0.933	1.013	0.000	1.946
^3TS_C	0.708	0.535	0.624	1.867
INT_C	0.715	0.367	0.778	1.860
TSr_C	0.718	0.299	0.766	1.783

[7] G. Jansen and B. A. Hess, *Phys. Rev. A*, 1989, **39**, 6016.
[8] Values from : X. F. Lin, C. Y. Zhao, C. M. Che, Z. F. Ke and D. L. Phillips, *Chem. Asian J.*, 2007, **2**, 1101.

Table S2-4. Calculated Activation Barrier for the Reaction of **^{3}NR$_A$** with Various Hydrogen Sources at the PBE/BS2 Level of Theory (ΔG_{sol}, kcal/mol in ethyl acetate).

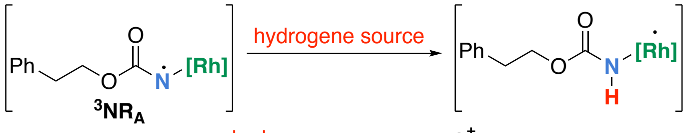

hydrogen source	$\Delta G^\ddagger$ kcal/mol
H_2O	43.6
CH_2Cl_2	38.4
HOAc	41.6[a]<br>35.4[b]
Salt-K_A	32.4

[a]Acetic acid coordinated to the catalyst.
[b]Acetic acid not coordinated to the catalyst.

Table S2-5. Calculated Activation Barrier for the Reaction of **^{3}NR$_E$** with Various Hydrogen Sources at the PBE/BS2 Level of Theory (ΔG_{sol}, kcal/mol in ethyl acetate).

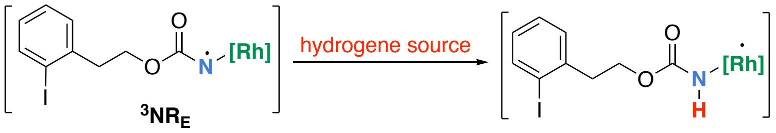

hydrogen source	$\Delta G^\ddagger$ kcal/mol
H_2O	45.9
CH_2Cl_2	40.7
HOAc	39.8[a]<br>37.2[b]

[a]Acetic acid coordinated to the catalyst.
[b]Acetic acid not coordinated to the catalyst.

Scheme S2-1. Calculated Activation Barrier for the α-C-H insertion for **^{1}NR$_A$** at the PBE/BS2 Level of Theory (ΔG_{sol}, kcal/mol in ethyl acetate).

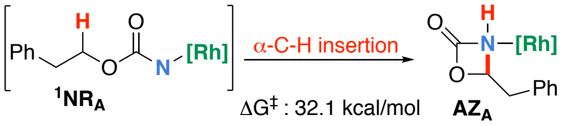

Scheme S2-2. Intermolecular Hydride transfer from **Salt-K$_A$** to **^{1}NR$_A$** affording 2-phenyl-acetaldehyde.

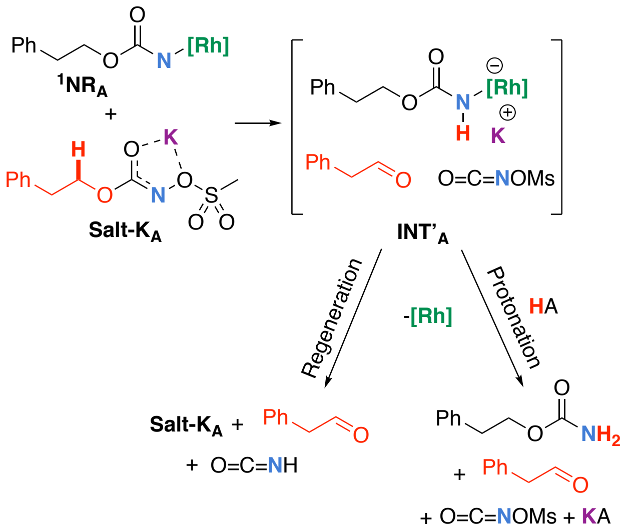

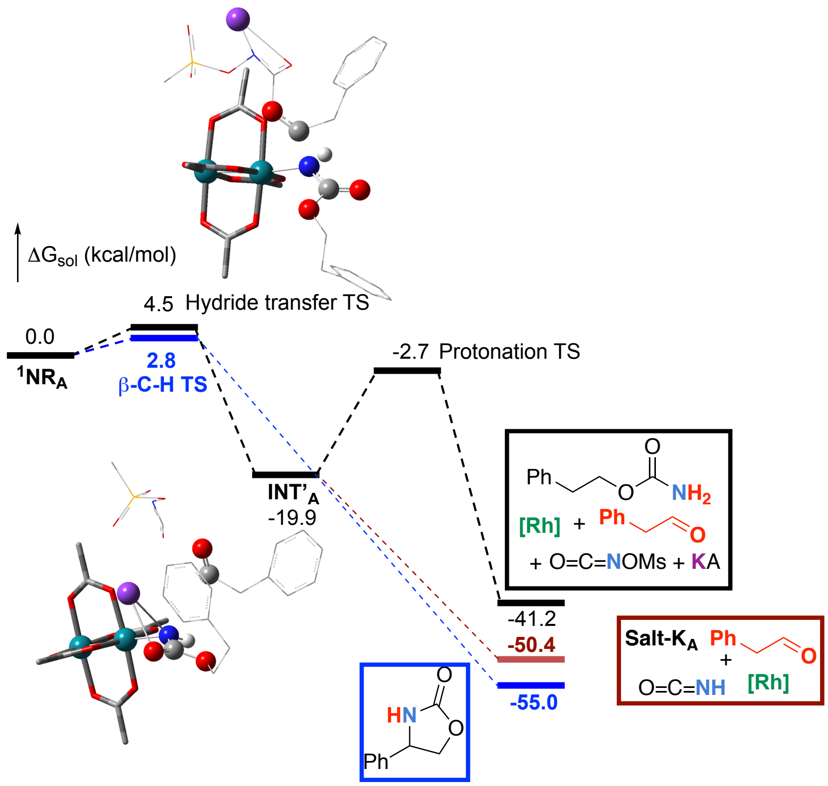

Figure S2-1. Calculated Free Energy Profile of the Formation of 2-Phenylacetaldehyde from **^{1}NR$_A$** at the PBE/BS2 Level of theory (the relative free energies (ΔG$_{sol}$, kcal/mol in ethyl acetate are provided). (Different atom styles are used for clarity purposes).

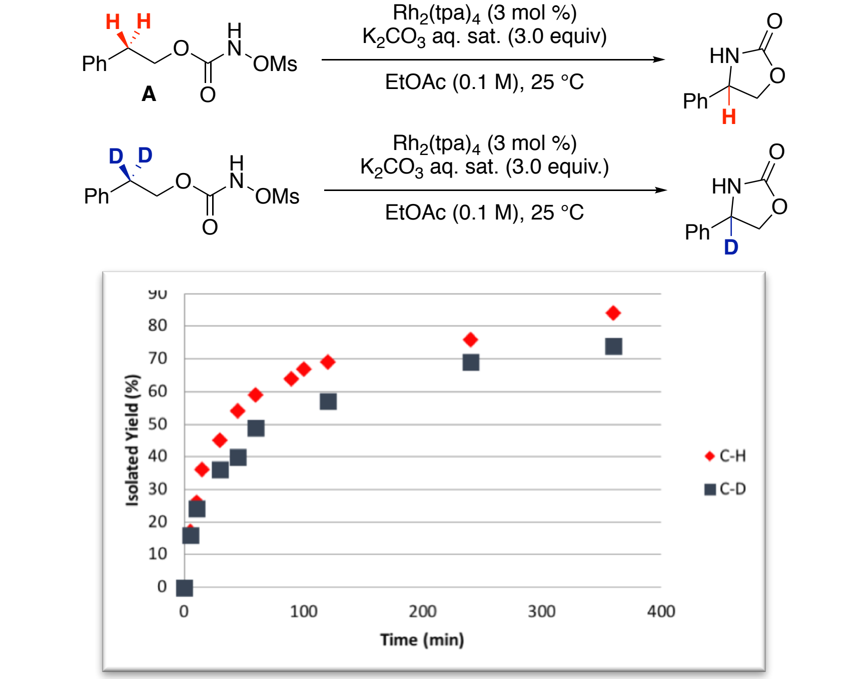

Figure S2-2. Rate Comparison for the C-H vs C-D Amination with *N*-Mesyloxycarbamate **A**.

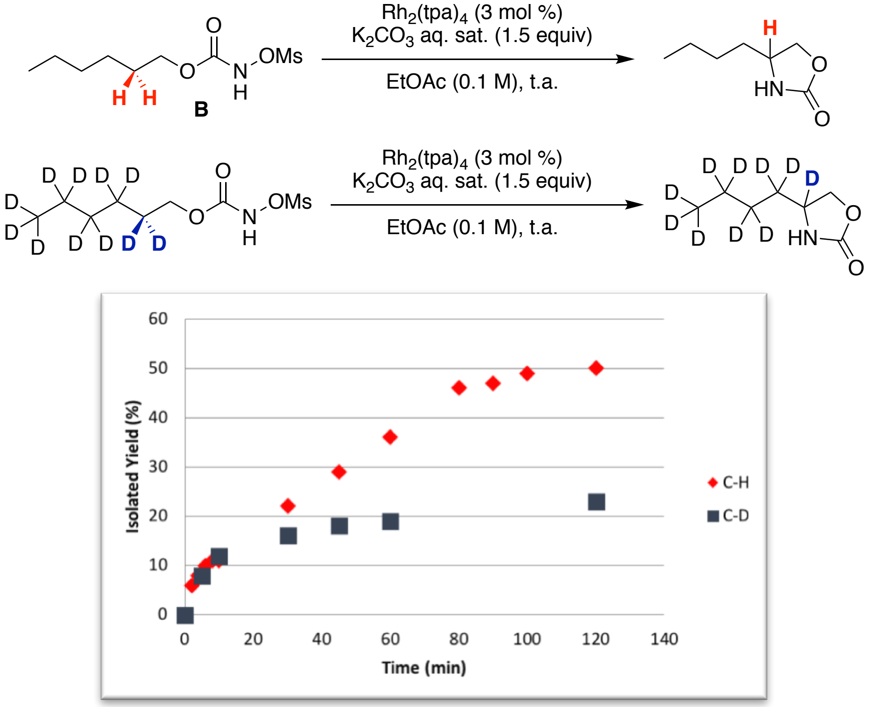

Figure S2-3. Rate Comparison for the C-H vs C-D Amination with *N*-Mesyloxycarbamate **B**.

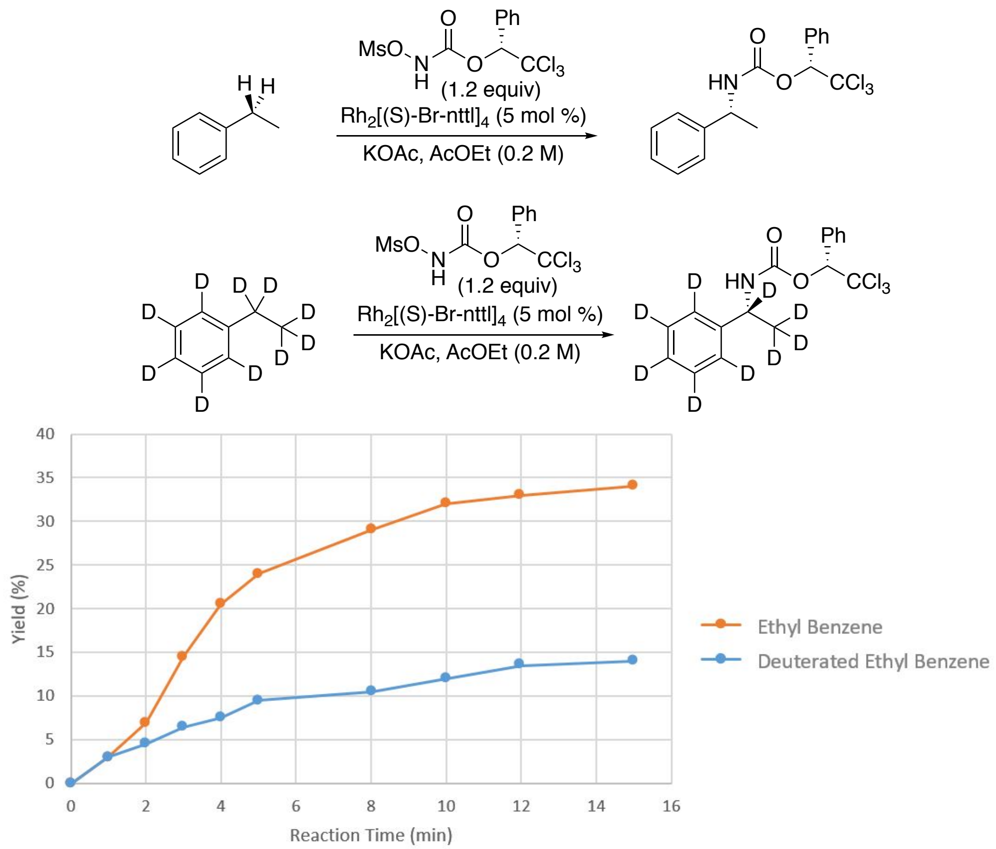

Figure S2-4. Rate Comparison for the Amination of Ethyl Benzene and Deuterated Ethyl Benzene with *N*-Mesyloxycarbamate **C**.

S3. Procedures for the kinetic isotope effect study

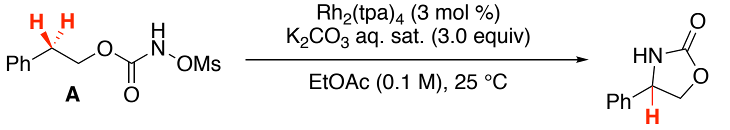

(±)-4-Phenyloxazolidin-2-one. *N*-mesyloxycarbamate **A** (0.130 g, 0.500 mmol) was dissolved in non-anhydrous EtOAc (5.00 mL). Green $Rh_2(tpa)_4$ (20.3 mg, 0.015 mmol, 3.00 mol %) was added and the resulting mixture was stirred. After complete dissolution of the rhodium dimer, a saturated aqueous K_2CO_3 solution (0.187 mL, 3.00 equiv) was added. The resulting turquoise heterogeneous mixture was stirred at room temperature. The reaction was quenched by adding a drop of pyridine to the reaction mixture at given times. The crude mixture was filtered through Celite, and the later was thoroughly washed with EtOAc. The solvent was evaporated under reduced pressure. The residue was chromatographed on silica gel eluting with 20% then 40% EtOAc/hexanes to afford the desired oxazolidinone as a white solid. R_f 0.17 (40% EtOAc/Hexanes); mp 135-136 °C (lit. 135-136 °C);[9] ^{1}H NMR (500 MHz, $CDCl_3$) δ 7.43-7.33 (m, 5H), 5.73 (s (br), 1H), 4.96 (dd, J = 8.0, 1H), 4.74 (t, J = 8.7 Hz, 1H,), 4.19 (dd, J = 8.6, 7.0 Hz, 1H); ^{13}C NMR (125 MHz, $CDCl_3$) δ 159.5, 139.4, 129.2, 128.9, 126.0, 72.5, 56.4.

[9] H. Lebel, K. Huard and S. Lectard, *J. Am. Chem. Soc.*, 2005, **127**, 14198.

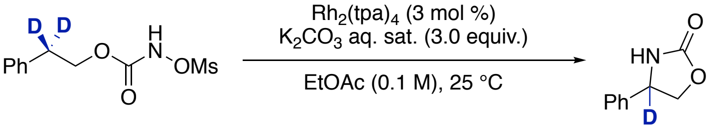

(±)-4-Phenyloxazolidin-2-one-4-*d*. The title compound was prepared according to the procedure using *N*-mesyloxycarbamate **A**. R_f 0.17 (40% EtOAc/Hexanes); mp 121.4-123.2 °C; ^{1}H NMR (500 MHz, $CDCl_3$) δ 7.41-7.33 (m, 5 H), 5.62 (s (br), 1H), 4.73 (d, *J* = 8.6 Hz, 1H), 4.19 (d, 8.6 Hz, 1H); **^{13}C NMR** (125 MHz, $CDCl_3$) δ 159.6, 139.5, 129.4, 129.0, 126.2, 72.6, 56.2 (t, *J* = 21.9 Hz).

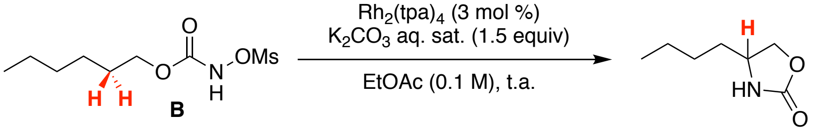

(±)-4-Butyloxazolidin-2-one. The title compound was prepared according to the procedure using *N*-mesyloxycarbamate **A**. ^{1}H NMR (500 MHz, $CDCl_3$) δ 6.35 (s (br), 1H), 4.47 (t, *J* = 8.5 Hz, 1H), 4.00 (dd, *J* = 8.5, 6.0 Hz, 1H), 3.89-3.82 (m, 1H), 1.63-1.50 (m, 2H), 1.36-1.23 (m, 4H), 0.90 (t, *J* = 7.0 Hz, 3H); ^{13}C NMR (125 MHz, $CDCl_3$) δ 160.1, 70.3, 52.6, 35.0, 27.3, 22.4, 13.8.

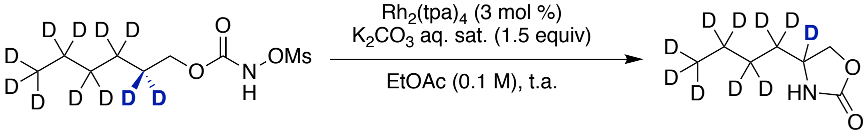

4-(Butyl-*d*$_9$)oxazolidin-2-one-4-*d*. The title compound was prepared according to the procedure using *N*-mesyloxycarbamate **A**. R_f 0.16 (40% EtOAc/Hexanes); ^{1}H NMR (500 MHz, $CDCl_3$) δ 5.62 (br s, 1H), 4.73 (d, *J* = 8.6 Hz, 1H), 4.19 (d, *J* = 8.6 Hz, 1H); ^{13}C NMR (125 MHz, $CDCl_3$) δ 159.7, 70.3, 53.5, 52.4, 33.9, 29.8, 21.3; **IR** (neat) 3284, 2921, 2873, 2212, 1749, 1447, 1287, 1178, 1037, 700 cm^{-1}; **HRMS** (ESI-TOF) m/z: $[M+H]^+$ calcd for $C_7H_4D_{10}NO_2$ 154.16467; found 154.16407.

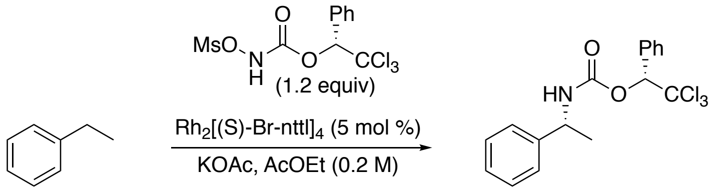

(*R*)-2,2,2-trichloro-1-phenylethyl ((*R*)-1-phenylethyl)carbamate. In a 4-mL vial equipped with a magnetic stirrer, ethylbenzene (16 mg, 0.15 mmol, 1.00 equiv) was dissolved in EtOAc (0.5 mL). $Rh_2[(S)\text{-Br-nttl}]_4$ (13 mg, 0.0075 mmol, 5 mol %), potassium acetate (44 mg, 0.45 mmol, 3.00 equiv) and *N*-mesyloxycarbamate (47 mg, 0.18 mmol, 1.2 equiv) were successively added to the solution. The resulting green solution was stirred at room temperature. The reaction was quenched by adding a drop of pyridine to the reaction mixture at given times. The solution was then diluted with EtOAc (2 mL). Celite was added to the solution and the resulting heterogeneous mixture was filtered over a short pad of celite, and the residue was washed with EtOAc (2 x 2 mL). A short amount of silica was then added to the solution and the resulting mixture was evaporated to dryness. The crude adsorbed on silica was then purified by flash chromatography (10% Et_2O/Hexanes) to afford the desired product. R_f 0.22 (20% Et_2O/Hexanes); ^{1}H NMR (500 MHz, DMSO-*d6*, 100 °C) δ 7.88 (br, 1H), 7.64-7.62 (m, 2H), 7.42-7.39 (m, 3H), 7.30-7.24 (m, 4H),

7.19-7.17 (m, 1H), 6.23 (s, 1H), 4.79-4.67 (m, 1H), 1.43 (d, J = 7 Hz, 3H); ^{13}C NMR (75 MHz, DMSO-*d6*, 25 °C) δ 153.2, 144.5, 133.6, 129.5, 129.4, 128.2, 127.9, 126.7, 125.7, 99.9, 81.9, 50.5, 22.8.

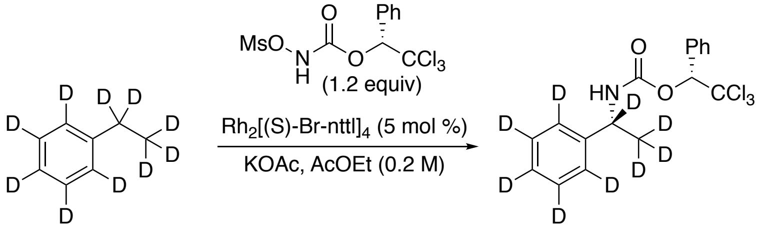

(*R*)-2,2,2-trichloro-1-phenylethyl ((*R*)-1-(phenyl-d5)ethyl-1,2,2,2-d4)carbamate. The title compound was prepared according to the procedure starting from ethyl benzene. R_f 0.22 (20% Et_2O/Hexanes); ^{1}H NMR (500 MHz, DMSO-*d6*, 100 °C) δ 7.87 (br, 1H), 7.63-7.60 (m, 2H), 7.41-7.39 (m, 3H), 6.23 (s, 1H); ^{13}C NMR (75 MHz, DMSO-*d6*, 25 °C) δ 153.2, 144.2, 133.6, 129.5, 129.4, 127.9, 127.7 (t, J = 24 Hz), 126.2 (t, J = 25 Hz), 125.3 (t, J = 24 Hz), 99.9, 81.9, 49.9 (t, J = 20 Hz), 21.8 (m); IR (neat) 3271, 3036, 2943, 1723, 1702, 1535, 1250, 1203, 1112, 1065, 1025, 825, 786, 695, 631, 613, 552, 488, 464 cm-1; $[\alpha]_D^{25}$ + 37.0 (c 1.00, $CHCl_3$); HRMS (ESI+) calc. for $C_{17}H_8D_9Cl_3NO_2$ $[M+H]^+$: 381.08843 ; found: 381.08989.

S4. Synthesis of the carbamate from 2-iodophenetyl-*N*-mesyloxycarbamate

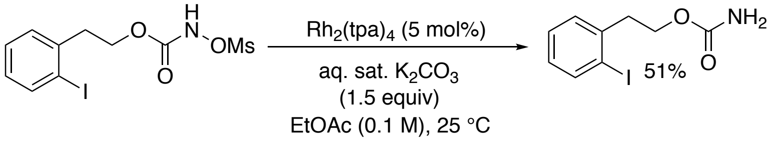

In a 15 mL round-bottom flask, equipped with a magnetic stir bar, the 2-iodophenetyl-*N*-mesyloxycarbamate (193 mg, 0.500 mmol) is dissolved in EtOAc (5.0 mL). $Rh_2(tpa)_4$ (20.4 mg, 3.00 mol %) is added. After complete dissolution of the rhodium dimer, an aqueous saturated solution of K2CO3 is added (93.4 µL, 0.750 mmol, 1.50 equiv). The green homogenous solution becomes heterogeneous and the mixture is stirred at room temperature for 16 hours. The crude mixture is filtered through Celite and the latter is thoroughly washed with EtOAc. The solvent is evaporated under reduced pressure. The residue is chromatographed on silica gel eluting with EtOAc/Hexanes 2 : 8 then 4 : 6 to afford the primary carbamate (74 mg, 0.26 mmol, 51% yield). R_f 0.46 (EtOAc /Hexanes 4 : 6); mp 132.2-133.6 °C ; ^{1}H NMR (300 MHz, $CDCl_3$) δ 7.83 (dd, J = 8.0 Hz, 1.1 Hz, 1H), 7.27-7.25 (m, 2H), 6.92 (td, J = 7.8 Hz, 2.1 Hz, 1H), 4.56 (s (br), 2H), 4.28 (t, J = 7.0 Hz, 2H), 3.08 (t, J = 6.8 Hz, 2); ^{13}C NMR (75 MHz, CDCl3) δ 156.7, 140.6, 100.8, 139.7, 130.2, 128.5, 64.2, 56.4; IR (neat) 3395, 3255, 1680, 1416, 1347, 1076,752 cm^{-1}; HRMS (ESI-TOF) m/z: $[M+H]^+$ calcd for $C_9H_{11}INO_2$ 291.9826; found 291.9819.

S5. Cartesian coordinates, total energies (a.u), vibrational zero-point energies (a.u) free energies and solvation free enthalpies (a.u, at 298.15 K, and 1 atm) for the stationary structures

S5-1. Deprotonation step of **A**, **B** & **C** with KOAc (Figure 1).

Base KOAc

C 0.965003 0.001556 -0.016115
O 0.379010 1.138771 -0.007424
O 0.380493 -1.137010 -0.007488
C 2.501456 -0.000164 0.005234
H 2.846490 -0.047399 1.054174
H 2.898115 -0.885423 -0.516129
H 2.902372 0.923025 -0.441258
K -1.869566 -0.000665 0.004621

Egas = -827.956705300
No imaginary frequency
Zero-point correction= 0.048976
Sum of electronic and thermal Enthalpies= -827.900495
Sum of electronic and thermal Free Energies= -827.939682
Gsolv= -828.089963

***N*-Mesyloxycarbamate A**

C -0.829338 -0.346740 -0.006992
O -0.630533 0.845662 -0.119593
N -2.087750 -0.986796 -0.107097
H -2.034267 -1.951406 -0.450358
O -3.001259 -0.279622 -0.922621
S -4.231516 0.462594 0.044944
C -5.150815 -1.005334 0.562548
H -4.464786 -1.658329 1.121134
H -5.967501 -0.664165 1.215663
H -5.550112 -1.504710 -0.331020
O -3.631598 1.082156 1.232292
O -4.990953 1.209677 -0.964202
O 0.077944 -1.313292 0.314183
C 1.414664 -0.813557 0.596867
H 1.330914 0.136842 1.148225
H 1.864712 -1.579242 1.247658
C 2.228997 -0.626021 -0.690025
H 2.254934 -1.583765 -1.239785
H 1.708000 0.113157 -1.322513
C 3.632882 -0.155170 -0.370626
C 4.688846 -1.073601 -0.220397
C 3.896252 1.213515 -0.167451
C 5.977314 -0.637545 0.118345
H 4.498946 -2.141902 -0.379644
C 5.182759 1.652862 0.172022
H 3.082352 1.938822 -0.283749
C 6.227363 0.727902 0.316232
H 6.788286 -1.365926 0.223927
H 5.370848 2.721469 0.319818
H 7.233837 1.070705 0.577588

Egas = -1216.73582533
No imaginary frequency
Zero-point correction= 0.225597
Sum of electronic and thermal Enthalpies= -1216.491944
Sum of electronic and thermal Free Energies= -1216.559042
Gsolv= -1216.834057

TS deprotonation of A with KOAc

C 0.233480 -1.093808 -1.055374
O 0.026030 -2.298100 -1.347310
N -0.483726 -0.149169 -1.714050
H -2.321955 0.560474 1.479580
O -0.210894 1.225502 -1.039129
S -1.515303 1.807773 -0.297220
C -1.057919 3.543790 -0.315461
H -0.995682 3.860694 -1.365732
H -1.842583 4.093239 0.223763
H -0.086807 3.651880 0.187775
O -2.775745 1.534807 -1.010345
O -1.569874 1.483154 1.223084
O 1.147186 -0.705258 -0.112322
C 1.856771 -1.790064 0.525399
H 2.367350 -2.399322 -0.240213
C 2.853240 -1.174015 1.512269
O -2.939442 -0.634052 1.319504
C -4.250450 -0.605228 1.147403
O -4.831587 -1.442764 0.432736
C -5.015863 0.507102 1.853574
H -4.732283 0.560871 2.917910
H -4.762702 1.478145 1.391137
H -6.098528 0.343430 1.761652
K -2.659676 -1.840707 -1.163942
H 1.135444 -2.440012 1.053431
H 2.293187 -0.493856 2.181234
H 3.237338 -1.994859 2.145133
C 4.016784 -0.446603 0.866551
C 5.313346 -0.992221 0.912265
C 3.830562 0.781157 0.198895
C 6.397025 -0.336170 0.311012
H 5.475754 -1.945220 1.430244
C 4.910986 1.437882 -0.404497
H 2.825103 1.209138 0.140553
C 6.198582 0.883480 -0.349924
H 7.397527 -0.779042 0.361488
H 4.746814 2.389608 -0.921613
H 7.042149 1.399952 -0.819963

Egas = -2044.70055578
One imaginary frequency of -870.7318
Zero-point correction= 0.268912
Sum of electronic and thermal Enthalpies= -2044.405914
Sum of electronic and thermal Free Energies= -2044.491214
Gsolv= -2044.917579

Salt-K_A

C 0.477146 -0.976410 -0.800772
O 0.873479 -1.915128 -0.056020
N 1.125852 0.065440 -1.363385
O 2.570280 -0.088156 -1.004823
S 3.051876 1.109977 0.026143
C 2.575629 2.605961 -0.843464
H 1.542056 2.432576 -1.182217
H 2.648499 3.446060 -0.138743
H 3.255318 2.733205 -1.697476
O 2.284883 1.026489 1.302255
O 4.523212 0.941690 0.098045
O -0.845204 -0.913844 -1.207861
C -1.698006 -1.935765 -0.675590
H -1.575802 -2.006509 0.420333
C -3.141173 -1.575563 -1.049081
K 2.877284 -1.702692 1.391930
C -3.693911 -0.365849 -0.321931
C -3.241127 0.934844 -0.624149
C -4.666239 -0.517827 0.684500
C -3.748035 2.045549 0.063168
H -2.476091 1.066445 -1.394950
C -5.176592 0.592247 1.372946
H -5.032566 -1.522720 0.928097
C -4.718183 1.879938 1.063247
H -3.384136 3.048378 -0.185967
H -5.936226 0.449740 2.149336
H -5.116170 2.750269 1.595876
H -3.180341 -1.419962 -2.143285
H -3.770430 -2.457968 -0.830201
H -1.423750 -2.917229 -1.108085

Egas = -1815.87517908
No imaginary frequency
Zero-point correction= 0.213024
Sum of electronic and thermal Enthalpies= -1815.642121
Sum of electronic and thermal Free Energies= -1815.714508
Gsolv= -1816.051351

_N_-Mesyloxycarbamate B

C -0.660251 -0.401925 0.038740
O -0.463815 0.557329 0.756137
N -1.876286 -0.700595 -0.624573
H -1.748694 -1.212140 -1.503999
O -2.648343 0.452833 -0.898081
S -4.036869 0.512317 0.134329
C -5.004731 -0.845035 -0.564898
H -4.408868 -1.765586 -0.483511
H -5.923294 -0.932244 0.033897
H -5.242930 -0.606334 -1.610643
O -3.659270 0.158551 1.507653
O -4.627874 1.803191 -0.237189
O 0.192019 -1.431767 -0.226213
C 1.464258 -1.362809 0.480265
H 1.284786 -0.948491 1.486170
H 1.782522 -2.414417 0.567329
C 2.485570 -0.527055 -0.284880
H 2.583102 -0.926793 -1.311951
H 2.101697 0.505654 -0.370155
C 3.856153 -0.519409 0.408969
H 4.224389 -1.559464 0.517487
H 3.745030 -0.124237 1.438069
C 4.905571 0.312860 -0.341955
H 4.535551 1.351310 -0.454332
H 5.019618 -0.082900 -1.371133
C 6.276645 0.334041 0.348454
H 6.642499 -0.704992 0.464428
H 6.160352 0.731940 1.375353
C 7.319416 1.164124 -0.408933
H 6.993748 2.214493 -0.513351
H 8.292064 1.165479 0.112171
H 7.484828 0.765972 -1.426105

Egas = -1143.00697264
No imaginary frequency
Zero-point correction= 0.257764
Sum of electronic and thermal Enthalpies= -1142.729933
Sum of electronic and thermal Free Energies= -1142.798795
Gsolv= -1143.060444

TS deprotonation of B with KOAc

C 0.116589 -1.380679 -1.096078
O -0.220011 -2.591346 -1.133908
N -0.603189 -0.503880 -1.841859
H -1.893689 0.893429 1.392965
O -0.150573 0.937637 -1.468595
S -1.290623 1.744985 -0.672785
C -0.743176 3.408534 -1.067313
H -0.829552 3.537493 -2.155103
H -1.399904 4.105009 -0.527031
H 0.300083 3.517182 -0.739069
O -2.658771 1.461845 -1.143214
O -1.136693 1.691445 0.874546
O 1.166126 -0.923144 -0.347576
C 1.836812 -1.938568 0.436859
H 2.170121 -2.753349 -0.230971
C 3.012946 -1.271470 1.139648
O -2.604549 -0.250277 1.535875
C -3.921069 -0.131746 1.568534
O -4.666165 -1.013758 1.102961
C -4.484572 1.139096 2.192852
H -4.031379 1.320488 3.181758
H -4.240477 2.004671 1.551008
H -5.576756 1.065969 2.288706
K -2.807894 -1.843091 -0.718304

H 1.123293 -2.371073 1.161770
H 3.461460 -2.017236 1.824616
H 2.630575 -0.450772 1.776268
C 4.083812 -0.733728 0.179656
H 4.463053 -1.567834 -0.444470
H 3.611767 -0.017692 -0.518396
C 5.260056 -0.058452 0.897634
H 5.725742 -0.776893 1.602487
H 4.879408 0.774587 1.522807
C 6.334094 0.478533 -0.059459
H 6.716320 -0.354657 -0.680806
H 5.866656 1.192804 -0.765144
C 7.502688 1.158233 0.663450
H 8.009561 0.456107 1.349489
H 8.257833 1.534554 -0.048029
H 7.154172 2.015620 1.267093

(UPBE-PBE) = -1970.97252467
One imaginary frequency of -859.0250
Zero-point correction= 0.300991
Sum of electronic and thermal Enthalpies= -1970.644807
Sum of electronic and thermal Free Energies= -1970.732046
Gsolv= -1971.144331

Salt-K_B

C 0.356807 -0.596804 -0.652490
O 0.879509 -1.718243 -0.399260
N 0.901284 0.595975 -0.979916
O 2.380049 0.379164 -1.067238
S 3.161498 1.154373 0.164216
C 2.581690 2.845556 0.006429
H 1.488943 2.770680 -0.110621
H 2.867962 3.389897 0.917143
H 3.046204 3.281205 -0.889051
O 2.718187 0.609217 1.479207
O 4.595905 1.012477 -0.186607
O -1.017243 -0.445165 -0.651023
C -1.761077 -1.629969 -0.324914
H -1.426064 -2.029074 0.652121
C -3.237450 -1.246969 -0.284183
K 3.206685 -1.954330 0.421683
H -1.571414 -2.413869 -1.082423
H -3.523512 -0.849498 -1.275130
H -3.823412 -2.175501 -0.133281
C -3.575358 -0.233849 0.820072
H -3.220981 -0.639553 1.788563
H -2.994337 0.690074 0.643767
C -5.070680 0.106219 0.941244
H -5.647800 -0.824320 1.120284
H -5.220766 0.732300 1.842979
C -5.665602 0.843102 -0.268813
H -5.574978 0.214814 -1.174855
H -5.065642 1.752550 -0.468464
C -7.135534 1.229823 -0.067174
H -7.540492 1.762108 -0.945194
H -7.763986 0.336694 0.101771
H -7.257727 1.889721 0.810722

Egas = -1742.14541138
No imaginary frequency
Zero-point correction= 0.245384
Sum of electronic and thermal Enthalpies= -1741.879127
Sum of electronic and thermal Free Energies= -1741.952313
Gsolv= -1742.275175

***N*-Mesyloxycarbamate C**

C 1.041290 0.493016 -0.266717
O 1.136591 0.625612 -1.466428
N 2.086117 0.563435 0.669390
H 1.801126 0.863221 1.606920
O 3.171506 1.355081 0.243600
S 4.526410 0.355461 -0.194273

C 5.033378 -0.217819 1.443078
H 4.194812 -0.773511 1.887077
H 5.896751 -0.884418 1.300214
H 5.311777 0.654187 2.050847
O 4.060640 -0.796429 -0.974383
O 5.473510 1.354189 -0.701332
O -0.096688 0.179549 0.448879
C -1.263852 -0.092902 -0.350593
H -0.955138 -0.137161 -1.409927
C -1.724515 -1.539905 0.020843
Cl -2.153095 -1.678489 1.765018
Cl -3.155140 -1.971722 -0.982561
Cl -0.383471 -2.689931 -0.344702
C -2.298823 0.996926 -0.155241
C -3.062606 1.425434 -1.255792
C -2.486870 1.612445 1.096187
C -4.017408 2.438885 -1.104454
H -2.905327 0.964242 -2.236414
C -3.438616 2.629018 1.244283
H -1.878451 1.301191 1.949410
C -4.209169 3.040768 0.147061
H -4.604517 2.764554 -1.968895
H -3.577011 3.102978 2.221375
H -4.951711 3.836496 0.265580

Egas = -2594.91917833
No imaginary frequency
Zero-point correction= 0.196353
Sum of electronic and thermal Enthalpies= -2594.700996
Sum of electronic and thermal Free Energies= -2594.776596
Gsolv= -2595.137309

TS deprotonation of C with KOAc

C	0.180616	-0.491426	1.726499
O	0.416912	0.460107	2.508513
N	-0.983906	-1.157488	1.831960
H	-2.671637	-0.249016	-1.394214
O	-1.022702	-2.217819	0.703185
S	-2.379757	-2.195043	-0.159522
C	-2.299579	-3.888160	-0.748405
H	-2.388841	-4.547979	0.125765
H	-3.138868	-4.028308	-1.444406
H	-1.336509	-4.034485	-1.257288
O	-3.591093	-1.897263	0.617383
O	-2.249227	-1.376734	-1.478601
O	1.082697	-0.859328	0.728177
C	2.161406	0.069614	0.584440
O	-2.820033	1.014514	-0.930234
C	-4.026847	1.410819	-0.558257
O	-4.177007	2.272083	0.328328
C	-5.217823	0.756052	-1.243980
H	-5.074391	0.723073	-2.336828
H	-5.314594	-0.284049	-0.884369
H	-6.141434	1.300229	-1.002363
K	-1.779066	1.732274	1.508660
H	2.614398	0.271831	1.571388
C	1.688341	1.384434	-0.022709
C	0.631519	1.407362	-0.952209
C	2.249711	2.600362	0.408207
C	0.143122	2.629628	-1.439628
H	0.170286	0.468654	-1.271911
C	1.770195	3.820057	-0.086808
H	3.056588	2.589924	1.148222
C	0.712762	3.838524	-1.010401
H	-0.698921	2.628699	-2.138297
H	2.215648	4.758624	0.258962
H	0.330536	4.791720	-1.390484
C	3.249488	-0.689908	-0.231213
Cl	2.658414	-1.167931	-1.863272
Cl	4.693393	0.378721	-0.415439
Cl	3.740523	-2.171240	0.673575

Egas = -3422.89084585
One imaginary frequency of -803.4315
Zero-point correction= 0.240021
Sum of electronic and thermal Enthalpies= -3422.621669

Sum of electronic and thermal Free Energies= -3422.713488
Gsolv= -3423.226381

Salt-K_C

C 0.807091 -0.506531 -0.266281
O 0.889707 0.042925 -1.390560
N 1.644325 -0.568756 0.786744
O 2.882397 0.146084 0.384029
S 4.140011 -0.866452 -0.100446
C 4.411687 -1.768454 1.437788
H 3.428536 -2.152680 1.749496
O 5.113991 -2.587990 1.228665
H 4.819528 -1.074925 2.185903
O 3.698716 -1.823131 -1.127446
O 5.231943 0.097114 -0.352544
O -0.416812 -1.099437 0.133249
C -1.518425 -0.411658 -0.446569
H -1.470419 -0.471721 -1.549741
C -2.791969 -1.206274 -0.042567
Cl -3.036693 -1.246456 1.739086
Cl -4.226746 -0.414416 -0.816738
Cl -2.656086 -2.889552 -0.658137
C -1.512402 1.061671 -0.037721
C -1.834149 2.064186 -0.972754
C -1.055272 1.439933 1.241784
C -1.710100 3.421694 -0.639382
H -2.171209 1.776504 -1.974071
C -0.933343 2.799042 1.577637
H -0.764423 0.664991 1.955259
C -1.257261 3.794789 0.639500
H -1.973324 4.188842 -1.375444
H -0.590734 3.079040 2.579828
H -1.173263 4.853755 0.906899
K 1.670528 2.309848 -0.537940

Egas = -3194.05968008
No imaginary frequency
Zero-point correction= 0.184194
Sum of electronic and thermal Enthalpies= -3193.852102
Sum of electronic and thermal Free Energies= -3193.930283
Gsolv= -3194.357755

HOAc

C 0.092557 0.126362 -0.000015
O 0.660242 1.206607 0.000002
O 0.770391 -1.063028 -0.000002
C -1.401460 -0.099973 -0.000008
H -1.697385 -0.684363 -0.887098
H -1.697321 -0.683927 0.887418
H -1.918886 0.868080 -0.000202
H 1.721942 -0.806761 0.000026

Egas = -228.822629146
No imaginary frequency
Zero-point correction= 0.060276
Sum of electronic and thermal Enthalpies=-228.756740
Sum of electronic and thermal Free Energies= -228.789935
Gsolv= -228.877029

S5-2. Deprotonation step of **A** with NaOAc (Figure 2).

Base NaOAc

C 0.520848 0.000959 -0.016227
O -0.076297 1.136052 -0.008414
O -0.074894 -1.134900 -0.008489
C 2.050507 0.000355 0.005292
H 2.394377 -0.045661 1.054026
H 2.446297 -0.884956 -0.515428
H 2.448541 0.923390 -0.442321
Na -1.955255 -0.000898 0.009505

Egas = -390.453848690
No imaginary frequency
Zero-point correction= 0.049486
Sum of electronic and thermal Enthalpies= -390.397377
Sum of electronic and thermal Free Energies=-390.435268
Gsolv= -390.552601

TS deprotonation of A with NaOAc

C 0.121768 -1.054063 -1.037625
O -0.106057 -2.247259 -1.376025
N -0.631533 -0.083928 -1.617195
H -2.695446 0.352844 1.486262
O -0.429836 1.221664 -0.800177
S -1.789766 1.762075 -0.110795
C -1.288572 3.476955 0.063114
H -1.134930 3.881069 -0.947012
H -2.101392 4.000460 0.586106
H -0.358799 3.511092 0.648089
O -2.988029 1.581572 -0.945276
O -1.985988 1.327945 1.376999
O 1.089871 -0.710384 -0.142234
C 1.819922 -1.823450 0.426775
H 2.316089 -2.387613 -0.381234
C 2.834566 -1.249367 1.419591
O -3.105273 -0.877760 1.097080
C -4.337630 -1.026913 0.646651
O -4.583117 -1.801552 -0.302142
C -5.432310 -0.213123 1.314457
H -5.363232 -0.292531 2.411768
H -5.305583 0.851252 1.046871
H -6.421583 -0.547213 0.972850
H 1.111496 -2.502089 0.934832
H 2.285453 -0.614244 2.139666
H 3.244078 -2.099289 1.995564
C 3.971248 -0.473418 0.782720
C 5.268459 -1.017236 0.736116
C 3.757199 0.797571 0.211722
C 6.325841 -0.317270 0.137674
H 5.452750 -2.003729 1.178514
C 4.811257 1.498458 -0.388572
H 2.750622 1.226880 0.226352
C 6.099823 0.945059 -0.427318
H 7.327571 -0.759538 0.115340
H 4.625461 2.483746 -0.829818
H 6.922860 1.495642 -0.894882
Na -2.403675 -1.765043 -1.106711

Egas = -1607.19690393
One imaginary frequency of -847.3709
Zero-point correction= 0.269586
Sum of electronic and thermal Enthalpies= -1606.902012
Sum of electronic and thermal Free Energies= -1606.984622
Gsolv= -1607.376696

Salt-Na$_A$

C 0.138475 1.665313 -0.394890
O -0.242320 1.125158 -1.473789
N 1.319800 1.641053 0.245731
O 2.280724 0.961062 -0.674600
S 2.853922 -0.428514 -0.013491
C 3.458365 0.105617 1.595582
H 2.665688 0.726397 2.038863
H 3.667272 -0.793065 2.192886
H 4.370466 0.695564 1.429109
O 1.723465 -1.385279 0.237786
O 3.961871 -0.863789 -0.879323
O -0.741153 2.443924 0.363395
C -2.089573 2.485563 -0.105890
H -2.116273 2.246400 -1.182183
C -3.008589 1.532783 0.690147
C -2.720077 0.062115 0.482404
C -1.943549 -0.664229 1.409013
C -3.202445 -0.618314 -0.657014
C -1.663976 -2.028230 1.213327
H -1.550612 -0.151070 2.292906
C -2.925235 -1.978839 -0.862974
H -3.804555 -0.071950 -1.391661
C -2.154255 -2.693481 0.075214
H -1.058974 -2.569430 1.947628
H -3.324980 -2.488521 -1.746321
H -1.953126 -3.759973 -0.071121
H -2.922399 1.781649 1.762523
H -4.050312 1.752003 0.388028
H -2.433523 3.522651 0.049676
Na -0.144144 -0.998200 -0.951988

Egas = -1378.38426668
No imaginary frequency
Zero-point correction= 0.214229
Sum of electronic and thermal Enthalpies= -1378.150793
Sum of electronic and thermal Free Energies=-1378.217805
Gsolv= -1378.510724

S5-3. Deprotonation step of **A** with LiOAc (Figure 2)

Base LiOAc

C 0.045820 0.000760 -0.013522
O -0.582194 1.124425 -0.003489
O -0.579141 -1.124684 -0.003550
C 1.565385 0.001400 -0.000065
H 1.913389 -0.043127 1.047255
H 1.956597 -0.885028 -0.521567
H 1.957760 0.925033 -0.450535
Li -2.068098 -0.002590 0.020894

Egas = -235.797251103
No imaginary frequency
Zero-point correction= 0.050703
Sum of electronic and thermal Enthalpies= -235.740054
Sum of electronic and thermal Free Energies= -235.775850
Gsolv= -235.883136

TS deprotonation of A with LiOAc

C -0.073938 -1.060251 -1.151854
O -0.352105 -2.245080 -1.504022
N -0.865238 -0.081912 -1.664358
H -2.752513 0.217706 1.565250

O -0.673391 1.175161 -0.779438
S -2.023817 1.697464 -0.038729
C -1.519547 3.411312 0.133233
H -1.406433 3.824387 -0.878570
H -2.312525 3.927285 0.692788
H -0.567704 3.442252 0.681655
O -3.240355 1.514156 -0.843072
O -2.178836 1.274240 1.461148
O 0.946223 -0.751822 -0.317793
C 1.680224 -1.886182 0.211947
H 2.210582 -2.389946 -0.614137
C 2.654251 -1.352818 1.265281
O -2.935794 -1.032736 1.078823
C -4.057061 -1.337051 0.468406
O -4.043005 -2.024233 -0.583254
C -5.358355 -0.819692 1.047844
H -5.365392 -0.911978 2.145660
H -5.454531 0.251891 0.796080
H -6.213351 -1.356271 0.613870
H 0.967230 -2.602850 0.654829
H 2.070748 -0.780405 2.010255
H 3.070106 -2.229156 1.795270
C 3.786772 -0.510086 0.710777
C 5.095756 -1.023718 0.653445
C 3.557234 0.794326 0.228524
C 6.149895 -0.261467 0.130139
H 5.291796 -2.035777 1.027659
C 4.608026 1.557517 -0.296917
H 2.541998 1.202355 0.253745
C 5.908510 1.033628 -0.347429
H 7.161080 -0.680920 0.097994
H 4.410517 2.568468 -0.669598
H 6.729003 1.632810 -0.755864
Li -2.238457 -1.738829 -1.188740

Egas = -1452.53659381
One imaginary frequency of -859.2017
Zero-point correction= 0.270966
Sum of electronic and thermal Enthalpies= -1452.240927
Sum of electronic and thermal Free Energies= -1452.321834
Gsolv= -1452.701112

Salt-Li$_A$
C 0.704021 -1.405752 -0.256263
O 1.023000 -2.030977 0.810087
N 1.418999 -0.587671 -1.052124
O 2.847440 -0.677958 -0.627132
S 3.371588 0.673481 0.169305
C 2.656517 2.044808 -0.746455
H 1.615931 1.766894 -0.972234
H 2.725622 2.935982 -0.106968
H 3.241275 2.162868 -1.669328
O 2.731653 0.669277 1.541630
O 4.839454 0.671113 0.097799
O -0.560186 -1.515590 -0.768011
C -1.526309 -2.196480 0.055763
H -1.398863 -1.904054 1.112236
C -2.918962 -1.818663 -0.460775
C -3.290542 -0.370898 -0.211127
C -2.767378 0.661270 -1.015858
C -4.146988 -0.024138 0.850774
C -3.093569 2.000314 -0.761995
H -2.092611 0.406510 -1.838951
C -4.475970 1.314687 1.106480
H -4.566487 -0.816707 1.482384
C -3.949229 2.332323 0.299157
H -2.681056 2.789084 -1.400517
H -5.149193 1.561631 1.934420
H -4.207728 3.378767 0.492637
H -2.961176 -2.051042 -1.540575
H -3.646446 -2.484685 0.038494
H -1.363887 -3.287647 -0.019177
Li 2.135260 -1.090451 1.850129

Egas = -1223.71497014
No imaginary frequency
Zero-point correction=0.214883

Sum of electronic and thermal Enthalpies= -1223.480989
Sum of electronic and thermal Free Energies= -1223.550390
Gsolv= -1223.832855

S5-4. Rhodium-Nitrene species formation of **A**, **B** & **C** with KOAc (Figure 3)

$Rh_2(O_2CCH_3)_4$

Rh 0.004526 -0.000009 0.038020
Rh 0.004447 0.000007 2.418407
O 2.050636 0.008873 0.081603
O 2.050576 0.008639 2.374886
O -2.042565 -0.008723 0.081564
O -2.042624 -0.008431 2.374796
O -0.004492 2.046593 0.081500
O -0.004309 2.046641 2.374773
O 0.014903 -2.046659 0.081764
O 0.014466 -2.046562 2.375027
C 2.633691 0.019700 1.228259
C -0.015837 2.629698 1.228127
C -2.625701 -0.019284 1.228165
C 0.012542 -2.629799 1.228420
C -0.028574 -4.140610 1.228088
H -1.081174 -4.473987 1.209764
H 0.470714 -4.536907 0.331598
H 0.441144 -4.537084 2.140177
C 4.143664 0.082279 1.228328
H 4.461590 1.139787 1.229464
H 4.546887 -0.397491 2.132281
H 4.546929 -0.395663 0.323434
C -0.079318 4.139690 1.228099
H -1.137037 4.456922 1.228324
H 0.399277 4.543405 2.132471
H 0.398897 4.543364 0.323509
C -4.135855 -0.081168 1.228104
H -4.454600 -1.138442 1.226217
H -4.538987 0.396201 2.133371
H -4.538981 0.399233 0.324420

E(RPBE-PBE) = -1134.27886099
No imaginary frequency
Zero-point correction= 0.205489
Sum of electronic and thermal Enthalpies= -1134.050375
Sum of electronic and thermal Free Energies= -1134.130265
Gsolv= -1134.415849

CO-K_A

C 1.960581 -1.065808 0.039885
O 1.052113 -0.577134 -0.744295
N 2.271056 -2.359102 -0.103854
O 3.312170 -2.753454 0.834584
S 4.850118 -2.675355 0.158301
C 4.813956 -4.135860 -0.910263
H 3.964368 -4.024641 -1.599739
H 5.761570 -4.170697 -1.467837
H 4.698483 -5.028663 -0.279736
O 4.991253 -1.490507 -0.709741
O 5.753262 -2.929623 1.292220
O 2.586998 -0.315202 0.969825
C 2.467855 1.123875 0.839289
H 1.427232 1.402002 0.616460
Rh -1.051831 -0.197053 -0.094603
Rh -3.352330 0.283035 0.450945
O -1.417590 -2.185295 0.426706
C -2.599404 -2.489290 0.858399
O -3.590921 -1.692421 0.964323
C -2.915570 -0.504573 -2.318920
O -1.669403 -0.700029 -2.029825
O -3.824333 -0.110597 -1.513526
O -0.839527 1.773401 -0.608861
C -1.847538 2.561719 -0.467881
O -3.015119 2.235049 -0.043959
O -2.776255 0.669563 2.371675
C -1.525195 0.591456 2.651179
O -0.582113 0.260831 1.840869

C 3.429281 1.658280 -0.234607
H 4.452928 1.321747 0.002931
H 3.151676 1.201610 -1.200916
H 2.738656 1.507039 1.835836
C 3.353974 3.166839 -0.327312
C 4.269250 3.986551 0.360144
C 2.334929 3.786241 -1.079964
C 4.175581 5.384715 0.296135
H 5.071025 3.519930 0.944634
C 2.239633 5.183512 -1.148190
H 1.612737 3.158502 -1.615151
C 3.160078 5.988529 -0.459223
H 4.902471 6.003656 0.833118
H 1.449643 5.646920 -1.750215
H 3.089297 7.079898 -0.515788
C -1.637894 4.005238 -0.867687
H -1.700284 4.090893 -1.967034
H -2.415542 4.641709 -0.421647
H -0.636249 4.344542 -0.560427
C -3.323191 -0.729809 -3.760322
H -3.094314 0.178972 -4.344485
H -2.755223 -1.561469 -4.206666
H -4.403026 -0.924494 -3.829784
C -2.827941 -3.935943 1.246338
H -3.136445 -4.515895 0.357467
H -1.903289 -4.378612 1.647131
H -3.636063 -4.005490 1.988916
C -1.108126 0.948542 4.059824
H -0.224320 0.364364 4.356651
H -0.837877 2.018693 4.095375
H -1.939297 0.778902 4.759799
K 0.141263 -2.832691 -1.714794

Egas = -2950.17486162
No imaginary frequency
Zero-point correction= 0.420685
Sum of electronic and thermal Enthalpies= -2949.710804
Sum of electronic and thermal Free Energies= -2949.836033
Gsolv= -2950.461065

TSCO-K$_A$

C	-1.805235	1.282087	-1.199597
O	-1.349502	0.667182	-2.188713
N	-0.913451	1.813766	-0.325812
O	-1.537205	2.560691	0.747054
S	-0.716027	4.017444	0.933222
C	0.571068	3.528335	2.095844
H	1.162231	2.720582	1.638560
H	1.190224	4.415437	2.299574
H	0.078448	3.177353	3.013660
O	-0.058975	4.405660	-0.343853
O	-1.665779	4.922016	1.598074
O	-3.131367	1.476646	-0.943473
C	-4.051110	0.910984	-1.901619
H	-3.570013	0.060936	-2.411842
C	-5.322950	0.492247	-1.154080
Rh	0.804982	-0.100359	-0.089234
Rh	2.417807	-1.840706	0.348631
C	1.104773	-0.799173	2.698590
O	0.439424	-0.017431	1.922848
O	2.012915	-1.633944	2.343200
O	-0.601519	-1.566224	-0.261113
C	-0.255817	-2.791897	-0.096050
O	0.925633	-3.219866	0.188602
O	2.340330	1.304084	0.081416
C	3.527377	0.855058	0.337416
O	3.853806	-0.369078	0.496928
O	2.764472	-2.019992	-1.659162
C	2.192340	-1.189139	-2.446968
O	1.381108	-0.248604	-2.104719
C	2.476655	-1.331538	-3.928087
H	3.247942	-2.094352	-4.103396
H	1.548435	-1.617624	-4.451333
H	2.807681	-0.367218	-4.349728
C	4.641933	1.877568	0.433497
H	4.238113	2.889714	0.583803
H	5.322187	1.614678	1.258205
H	5.235258	1.864243	-0.497937
C	-1.339368	-3.827574	-0.285841
H	-2.282667	-3.479203	0.163507
H	-1.517604	-3.965781	-1.366772

H	-1.031446	-4.789628	0.148710
C	0.761430	-0.750628	4.171437
H	0.304866	0.214947	4.433832
H	0.033731	-1.550107	4.396461
H	1.660681	-0.932322	4.779255
K	0.825462	2.445484	-2.216894
H	-4.289592	1.686726	-2.654156
H	-6.092989	0.268140	-1.915438
H	-5.687296	1.363869	-0.579479
C	-5.140898	-0.704996	-0.243051
C	-5.618487	-1.973129	-0.625273
C	-4.479465	-0.582126	0.996004
C	-5.448188	-3.091144	0.203956
H	-6.139300	-2.083423	-1.584244
C	-4.304133	-1.698296	1.826025
H	-4.093917	0.394766	1.304031
C	-4.790067	-2.956555	1.435750
H	-5.836674	-4.066228	-0.109523
H	-3.789466	-1.581785	2.785976
H	-4.663409	-3.825088	2.091082

Egas = -2950.17641400
One imaginary frequency of -41.0327
Zero-point correction= 0.420289
Sum of electronic and thermal Enthalpies= -2949.713507
Sum of electronic and thermal Free Energies= -2949.835621
Gsolv= -2950.459346

NRO-K_A

Rh 0.920402 0.047871 0.225258
Rh 2.985754 -1.007489 -0.523490
O 1.301954 -0.716030 2.084268
O 3.274787 -1.667427 1.387942
O 0.634009 0.724959 -1.738641
O 2.587798 -0.277910 -2.410735
O -0.048682 -1.715676 -0.384337
O 1.937343 -2.717857 -0.939308
O 2.046694 1.698633 0.697264
O 3.978645 0.717849 -0.062362
C 2.389217 -1.377164 2.271543
C 0.670182 -2.710046 -0.785127
C 1.537701 0.415334 -2.615156
C 3.309855 1.682855 0.453486
N -0.865426 1.050464 0.947639
C -1.757654 0.589708 1.894986
O -2.348856 1.189077 2.787827
O -1.986337 -0.763536 1.607753
C -2.930032 -1.409144 2.476761
C -4.390215 -1.068303 2.121131
H -2.732304 -1.122876 3.523136
H -2.725276 -2.485059 2.347750
H -5.044641 -1.737736 2.713258
H -4.589092 -0.036476 2.452225
C -4.706692 -1.198759 0.644205
C -4.517590 -2.415153 -0.048339
C -5.171287 -0.085624 -0.087010
C -4.773475 -2.513217 -1.424914
H -4.163037 -3.297509 0.496856
C -5.438524 -0.179058 -1.464323
H -5.313029 0.870435 0.428995
C -5.234915 -1.393307 -2.141092
H -4.627174 -3.469872 -1.938502
H -5.806376 0.699369 -2.005319
H -5.450884 -1.473509 -3.211842
O -0.698924 2.457221 1.154484
S -1.516980 3.394043 -0.005823
O -2.060969 4.566534 0.696209
O -2.403632 2.523608 -0.826046
C -0.128024 3.914087 -1.031287
H 0.592122 4.429974 -0.380433
H 0.320328 3.012197 -1.473287
H -0.519932 4.596604 -1.800992
C 4.072649 2.949590 0.781624
H 4.089702 3.607590 -0.105490
H 3.582874 3.490628 1.604715
H 5.113463 2.708575 1.044238
C 2.659178 -1.831886 3.689723
H 3.137398 -1.007651 4.247581
H 1.715622 -2.079059 4.199241
H 3.339849 -2.695651 3.692751
C -0.065025 -4.006029 -1.065197

H -0.238587 -4.533687 -0.110714
H -1.049580 -3.809682 -1.519655
H 0.533935 -4.655514 -1.719574
C 1.340358 0.966863 -4.013922
H 0.270451 1.075177 -4.251207
H 1.802593 1.968265 -4.077339
H 1.833472 0.318958 -4.753251
K -2.127587 -0.157298 -1.205268

Egas = -2950.19839531
No imaginary frequency
Zero-point correction= 0.420581
Sum of electronic and thermal Enthalpies= -2949.734609
Sum of electronic and thermal Free Energies= -2949.857155
Gsolv= -2950.481073

TSNR-K_A

Rh 0.243646 -0.301659 -0.269599
Rh 0.459161 -2.565519 0.551409
O -1.207794 0.049964 1.160149
O -1.030378 -2.115715 1.905270
O 1.654118 -0.771331 -1.679345
O 1.941648 -2.878885 -0.817286
O -1.183203 -1.063507 -1.555448
O -0.938500 -3.198314 -0.775360
O 1.618542 0.196970 1.192858
O 1.910148 -1.967729 1.838209
C -1.541639 -0.940732 1.917295
C -1.448527 -2.322045 -1.558753
C 2.236228 -1.914045 -1.617929
C 2.213470 -0.727536 1.873169
N 0.178477 1.453342 -1.052767
C -0.679529 2.402631 -0.561744
O -0.397820 3.248166 0.312938
O -1.871934 2.388625 -1.219803
C -2.814476 3.417731 -0.829865
C -4.187992 3.032132 -1.387302
H -2.475764 4.381133 -1.252638
H -2.829753 3.507865 0.269273
H -4.833032 3.926356 -1.304981
H -4.073825 2.821260 -2.466621
C -4.848278 1.864821 -0.680431
C -4.403992 0.542552 -0.885272
C -5.917147 2.082895 0.209955
C -5.019785 -0.527432 -0.219838
H -3.560950 0.356379 -1.558044
C -6.533838 1.014774 0.877742
H -6.277061 3.105554 0.376066
C -6.087871 -0.297283 0.662556
H -4.667023 -1.550465 -0.394133
H -7.368729 1.207883 1.559772
H -6.573528 -1.136197 1.172262
O 2.387137 2.370821 -0.782627
S 3.832843 1.968809 -0.479708
O 4.171493 0.527423 -0.656814
O 4.245951 2.528178 0.875134
C 4.813876 2.873087 -1.711272
H 4.513581 2.520571 -2.708846
H 4.616259 3.950244 -1.611483
H 5.876999 2.656608 -1.529481
C -2.661216 -0.675288 2.898243
H -2.462084 0.252660 3.458444
H -3.601243 -0.525973 2.338734
H -2.772612 -1.520701 3.591612
C -2.435411 -2.796498 -2.599725
H -3.287597 -2.102249 -2.668896
H -1.938870 -2.810404 -3.585492
H -2.783940 -3.811086 -2.359943
C 3.332106 -0.282265 2.780767
H 3.985039 0.419400 2.233245
H 2.916056 0.235992 3.664508
H 3.903089 -1.154810 3.128412
C 3.391401 -2.111736 -2.565789
H 3.090198 -1.837810 -3.589690
H 4.189745 -1.416729 -2.253563
H 3.748574 -3.150662 -2.535523
K 1.769776 2.983787 1.705074

Egas = -2950.17617019
One imaginary frequency of -23.9086
Zero-point correction= 0.419352
Sum of electronic and thermal Enthalpies= -2949.714396
Sum of electronic and thermal Free Energies= -2949.834119
Gsolv= -2950.458449

[1]NR$_A$

Rh 0.838018 0.685920 -0.351916
Rh 1.936001 -1.256323 0.584341
O -0.624043 0.471778 1.077498
O 0.413043 -1.377231 1.955978
O 2.369969 0.804112 -1.734793
O 3.404026 -1.037308 -0.830006
O -0.148992 -0.664953 -1.577531
O 0.915517 -2.476544 -0.676285
O 1.940701 1.792825 0.973365
O 2.968539 -0.048134 1.847966
C -0.530277 -0.509894 1.908911
C 0.107622 -1.922628 -1.503235
C 3.316809 -0.063778 -1.663333
C 2.747632 1.213198 1.790978
N -0.030602 2.161441 -1.195179
C -1.217107 2.622325 -0.695157
O -1.209870 3.625081 0.035421
O -2.323478 1.971212 -1.124039
C -3.584338 2.505847 -0.641747
C -4.699933 1.577709 -1.130687
H -3.715038 3.526727 -1.042343
H -3.555948 2.564835 0.459588
H -5.657077 2.104453 -0.961863
C -4.742434 0.221297 -0.452615
C -3.758356 -0.755713 -0.710153
C -5.769571 -0.087877 0.459360
C -3.807934 -2.004174 -0.072955
H -2.942262 -0.525292 -1.401755
C -5.821879 -1.335987 1.097671
H -6.544683 0.659197 0.668115
C -4.840084 -2.301030 0.832185
H -3.038185 -2.754681 -0.286656
H -6.634532 -1.555824 1.798103
H -4.880667 -3.280145 1.320969
C -1.661772 -0.650009 2.898974
H -1.924650 0.335185 3.314763
H -2.551290 -1.039431 2.372747
H -1.381148 -1.344834 3.703190
C -0.595911 -2.810196 -2.503151
H -1.663596 -2.547040 -2.566580
H -0.155910 -2.646592 -3.502096
H -0.476420 -3.866999 -2.225613
C 3.463877 2.104874 2.774959
H 3.739251 3.057171 2.297402
H 2.783549 2.333327 3.613825
H 4.354526 1.595495 3.169913
C 4.452629 0.108719 -2.646799
H 4.075991 0.512968 -3.598091
H 5.176437 0.833143 -2.233913
H 4.969621 -0.848362 -2.807165

E(RPBE-PBE) = -1687.15011792
No imaginary frequency
Zero-point correction= 0.368451
Sum of electronic and thermal Enthalpies= -1686.747182
Sum of electronic and thermal Free Energies= -1686.849384
Gsolv= -1687.277389

KOMs

S -0.780733 0.148141 -0.000034
O 0.041842 -0.172767 -1.244102
O -1.402821 1.498006 -0.001512

O 0.041668 -0.170055 1.244788
K 2.266357 -0.074565 -0.000004
C -2.135040 -1.056169 0.000756
H -1.718477 -2.073832 0.002136
H -2.743364 -0.891038 0.901972
H -2.742485 -0.893101 -0.901408

Egas = -1263.00949508
No imaginary frequency
Zero-point correction= 0.050437
Sum of electronic and thermal Enthalpies= -1262.950984
Sum of electronic and thermal Free Energies= -1262.991557
Gsolv= -1263.182840

CO-K$_B$

C -1.820030 1.078337 -1.011993
O -0.992343 0.207571 -1.473640
N -1.579384 2.360823 -1.284023
O -2.578273 3.264486 -0.691340
S -1.822838 4.242398 0.422050
C -1.699381 3.150517 1.854720
H -1.082716 2.282732 1.578179
H -1.226484 3.723368 2.666899
H -2.716687 2.842002 2.132400
O -0.436246 4.562035 -0.021313
O -2.777668 5.325596 0.694513
O -2.923560 0.732014 -0.296631
C -3.246169 -0.683676 -0.241370
H -2.319379 -1.273856 -0.174248
Rh 0.816371 -0.407786 -0.317104
Rh 2.765797 -1.251197 0.838832
O 1.139230 1.415308 0.635844
C 2.129675 1.508928 1.465919
O 2.970748 0.588631 1.740281
C 3.348527 -0.042327 -1.735008
O 2.091269 0.243529 -1.839977
O 3.909296 -0.641271 -0.756491
O 0.630402 -2.245358 -1.199888
C 1.480299 -3.161103 -0.896363
O 2.475045 -3.045460 -0.090069
O 1.546172 -1.851043 2.364884
C 0.284868 -1.647795 2.249755
O -0.308338 -1.063970 1.267605
C -4.083989 -1.096980 -1.450135
H -4.985556 -0.459259 -1.501034
H -3.493127 -0.890537 -2.360922
H -3.808456 -0.788007 0.700858
C -4.468128 -2.589105 -1.435029
H -5.005846 -2.818180 -2.375193
H -3.540321 -3.193530 -1.459344
C -5.331988 -3.052022 -0.245287
H -5.492593 -4.144435 -0.335578
H -4.778307 -2.912270 0.704663
C -6.699656 -2.360414 -0.135699
H -6.560626 -1.271293 0.002925
H -7.242829 -2.478107 -1.094081
C -7.555217 -2.910420 1.011975
H -7.048137 -2.780147 1.985214
H -8.531013 -2.398389 1.072838
H -7.750144 -3.990448 0.883185
C 1.267792 -4.517741 -1.531566
H 0.601319 -5.118591 -0.887896
H 0.785976 -4.408800 -2.514504
H 2.225408 -5.050684 -1.625963
C -0.599548 -2.170745 3.360400
H -1.515315 -1.566526 3.441848
H -0.893151 -3.209883 3.128771
H -0.052505 -2.175694 4.314750
C 2.291915 2.833395 2.183646
H 3.348658 3.144975 2.168524
H 1.653140 3.609169 1.734685
H 2.007702 2.706901 3.242990
C 4.248731 0.401726 -2.871342
H 3.665933 0.579859 -3.787287
H 4.774784 1.331783 -2.589882

H	5.018322	-0.362572	-3.058835
K	0.933339	2.786344	-1.754858

Egas = -2876.45444443
No imaginary frequency
Zero-point correction= 0.453446
Sum of electronic and thermal Enthalpies= -2875.957076
Sum of electronic and thermal Free Energies= -2876.081781
Gsolv= -2876.686835

TSCO-K$_B$

C	2.014306	0.279697	0.251528
O	1.723420	-0.608769	1.084976
N	1.058407	1.194548	-0.048474
O	1.509405	2.218048	-0.964323
S	1.185611	3.729914	-0.297062
C	-0.498484	3.998056	-0.881584
H	-1.131491	3.181266	-0.503029
H	-0.832544	4.975109	-0.500102
H	-0.474970	3.996006	-1.980571
O	1.146614	3.639501	1.188538
O	2.096805	4.668296	-0.968963
O	3.232414	0.405731	-0.344989
C	4.216961	-0.559068	0.092499
H	3.866782	-1.577366	-0.155802
C	5.530023	-0.228519	-0.605203
Rh	-1.132592	-0.173797	-0.052007
Rh	-3.255306	-1.311813	-0.209912
C	-2.570768	0.303795	-2.508436
O	-1.470344	0.603577	-1.912562
O	-3.512514	-0.434920	-2.043942
O	-0.285153	-1.817529	-0.925571
C	-1.037249	-2.798668	-1.270199
O	-2.310102	-2.883837	-1.101008
O	-2.111129	1.433475	0.848046
C	-3.391501	1.333150	1.008827
O	-4.116348	0.331569	0.690755
O	-2.936829	-2.181782	1.611317
C	-1.895306	-1.831177	2.268354
O	-1.002637	-0.985027	1.886929
C	-1.675262	-2.490235	3.614293
H	-2.552247	-3.086707	3.901968
H	-0.788709	-3.144666	3.556630
H	-1.472678	-1.730103	4.387440
C	-4.103814	2.532967	1.599101
H	-3.405031	3.176973	2.153911
H	-4.546733	3.127464	0.780532
H	-4.925962	2.204623	2.252917
C	-0.357367	-3.958154	-1.964192
H	-0.184412	-3.697343	-3.022940
H	0.623128	-4.151419	-1.502657
H	-0.988859	-4.857121	-1.920317
C	-2.758467	0.855434	-3.904812
H	-2.125182	1.740889	-4.060586
H	-2.463734	0.085119	-4.638927
H	-3.817357	1.099348	-4.079737
K	0.344644	1.310641	2.526411
H	4.320512	-0.505974	1.192495
H	5.381317	-0.281327	-1.700026
H	5.804261	0.818394	-0.375355
C	6.663323	-1.173594	-0.178982
H	6.786798	-1.118638	0.920229
H	6.377633	-2.221267	-0.403607
C	7.998607	-0.857418	-0.870201
H	8.296945	0.182229	-0.626903
H	7.844934	-0.881399	-1.966639
C	9.148933	-1.813368	-0.506366
H	10.016988	-1.583059	-1.152632
H	8.851237	-2.851110	-0.755113
C	9.588972	-1.745731	0.962374
H	9.892582	-0.719444	1.238428
H	8.780214	-2.049253	1.649423
H	10.448913	-2.411080	1.152224

Egas = -2876.44708932
One imaginary frequency of -43.3432
Zero-point correction= 0.452323

Sum of electronic and thermal Enthalpies= -2875.950923
Sum of electronic and thermal Free Energies= -2876.076501
Gsolv= -2876.686515

NRO-K$_B$

Rh 0.763278 0.028752 0.215970
Rh 2.668133 -1.383800 -0.346266
O -0.451149 -1.573795 -0.403092
O 1.372100 -2.908080 -0.796167
O 2.116101 1.495202 0.705361
O 3.903827 0.164640 0.147412
O 0.723511 0.652633 -1.789494
O 2.538477 -0.665817 -2.273940
O 0.892183 -0.698110 2.122300
O 2.704685 -2.009649 1.599020
C 0.116933 -2.687130 -0.730796
C 1.622427 0.162458 -2.585585
C 3.373138 1.248632 0.582308
C 1.829383 -1.531590 2.407838
N -0.897447 1.331640 0.752777
C -1.850598 1.053059 1.721244
O -2.297693 1.766901 2.610980
O -2.323503 -0.237840 1.464018
C -3.200672 -0.760806 2.489578
C -4.684799 -0.501096 2.204276
H -2.920674 -0.298020 3.448903
H -2.983607 -1.843463 2.526437
H -5.229893 -0.636972 3.158024
H -4.800577 0.566332 1.939259
O -0.499525 2.701250 0.930915
S -1.057913 3.724997 -0.298721
O -1.478423 4.982741 0.336173
O -1.990828 2.985710 -1.195169
C 0.483742 4.003816 -1.191542
H 1.205813 4.432703 -0.482230
H 0.838615 3.033402 -1.567646
H 0.273203 4.709817 -2.009460
C -5.339180 -1.405894 1.145193
H -6.420361 -1.171401 1.097987
H -5.270155 -2.461619 1.476957
C -4.747410 -1.294842 -0.267868
H -3.685268 -1.606993 -0.231033
H -4.770255 -0.225759 -0.573289
C -5.481226 -2.130851 -1.325859
H -5.489134 -3.191974 -1.010805
H -6.540904 -1.815774 -1.374093
C -4.850202 -2.019299 -2.719996
H -3.799454 -2.370013 -2.712509
H -5.388218 -2.627203 -3.467200
H -4.862968 -0.973215 -3.083782
C 1.555270 0.596558 -4.037272
H 1.007537 -0.163240 -4.623473
H 1.034350 1.560556 -4.139320
H 2.570155 0.666477 -4.456902
C -0.807921 -3.845832 -1.048859
H -1.347488 -4.145308 -0.133930
H -1.565724 -3.545411 -1.792753
H -0.235321 -4.702636 -1.430519
C 1.919099 -1.965149 3.855156
H 2.491291 -1.210620 4.423091
H 0.914161 -2.027520 4.299359
H 2.439460 -2.930601 3.935137
C 4.317487 2.344800 1.028146
H 3.874599 3.335614 0.845987
H 4.491407 2.249537 2.114406
H 5.284560 2.251637 0.512704
K -2.080571 0.334215 -1.480959

Egas = -2876.46058167
No imaginary frequency
Zero-point correction= 0.452446
Sum of electronic and thermal Enthalpies= -2875.963916
Sum of electronic and thermal Free Energies= -2876.089119
Gsolv= -2876.701325

TSNR-K_B

Rh -0.356661 -0.335516 -0.159087
Rh -0.772219 -2.703336 0.165714
O 1.217813 -0.856499 -1.381310
O 0.842864 -3.093548 -1.030472
O -1.925540 0.020513 1.135804
O -2.418891 -2.207629 1.293795
O 0.850499 -0.424494 1.568095
O 0.455863 -2.667376 1.808346
O -1.576737 -0.461345 -1.780641
O -1.966740 -2.697761 -1.480357
C 1.464062 -2.108062 -1.564359
C 1.001647 -1.568213 2.154084
C -2.658168 -0.972298 1.523727
C -2.122053 -1.581373 -2.095223
N -0.071533 1.587832 -0.297286
C 0.659560 2.074305 -1.377703
O 0.317035 2.255287 -2.538522
O 1.915965 2.375502 -0.880018
C 2.919639 2.702836 -1.873262
C 4.287368 2.441572 -1.251264
H 2.795716 3.765124 -2.152957
H 2.746672 2.094508 -2.778581
H 5.056534 2.874934 -1.919953
H 4.349158 3.001204 -0.299293
O -2.055956 2.376959 -0.865163
S -2.928022 3.135985 0.150471
O -2.147703 3.624779 1.349110
O -4.194184 2.418676 0.482969
C -3.400542 4.634935 -0.755199
H -2.485905 5.181121 -1.027031
H -3.949108 4.334833 -1.659541
H -4.040142 5.246208 -0.101259
C 4.577606 0.949194 -1.023888
H 3.731413 0.498247 -0.471764
H 4.601411 0.448888 -2.012230
C 5.892838 0.659020 -0.279814
H 6.116239 -0.423360 -0.357466
H 6.731039 1.177781 -0.788004
C 5.885507 1.046300 1.208047
H 5.693777 2.130892 1.316002
H 5.036132 0.535282 1.705808
C 7.192858 0.690256 1.925924
H 8.052761 1.211768 1.468716
H 7.161124 0.971250 2.992957
H 7.396596 -0.394245 1.870029
C 1.905583 -1.585761 3.369156
H 2.851265 -1.065622 3.145390
H 1.421080 -1.054307 4.207556
H 2.107416 -2.620879 3.678123
C -3.046682 -1.551354 -3.287999
H -3.990517 -1.059610 -2.995854
H -2.598671 -0.952608 -4.095923
H -3.260701 -2.572995 -3.632616
C -3.888731 -0.604258 2.317552
H -3.611781 -0.414974 3.370662
H -4.319503 0.321882 1.901664
H -4.614805 -1.429759 2.296151
C 2.588182 -2.430702 -2.522721
H 2.234893 -2.274072 -3.556767
H 3.438008 -1.751002 -2.355008
H 2.901691 -3.477840 -2.406961
K -0.393128 2.056432 2.304104

Egas = -2876.43926827
One imaginary frequency of -45.5637
Zero-point correction= 0.450848
Sum of electronic and thermal Enthalpies= -2875.944473
Sum of electronic and thermal Free Energies= -2876.070500
Gsolv= -2876.686082

^1NR_B

Rh 0.742951 0.499120 -0.393336
Rh 2.234803 -1.106189 0.634933
O -0.701515 -0.070716 0.959321
O 0.717995 -1.590487 1.933903
O 2.270216 0.973757 -1.700945
O 3.676171 -0.545775 -0.706435
O 0.174578 -1.042052 -1.645494
O 1.600706 -2.534070 -0.663704

O 1.454394 1.838909 0.988362
O 2.872827 0.318715 1.934762
C -0.399783 -0.971582 1.830711
C 0.706780 -2.206024 -1.522227
C 3.387689 0.347237 -1.582941
C 2.354341 1.487498 1.836937
N -0.362227 1.783835 -1.265889
C -1.470172 2.323218 -0.681163
O -1.335490 3.352217 0.003346
O -2.647502 1.744312 -1.001798
C -3.828006 2.477946 -0.564262
C -5.051856 1.674009 -0.978921
H -3.813663 3.477959 -1.032580
H -3.779619 2.613570 0.530829
H -5.943103 2.293309 -0.758124
H -5.032695 1.530849 -2.075981
C -5.173558 0.316350 -0.271766
H -4.272302 -0.287054 -0.491269
H -5.177748 0.485168 0.822905
C -6.429866 -0.461568 -0.690037
H -6.427966 -0.574264 -1.791904
H -7.330443 0.138025 -0.445886
C -6.560823 -1.854078 -0.047708
H -5.669402 -2.458010 -0.308582
H -7.424901 -2.373791 -0.502981
C -6.736782 -1.833334 1.476712
H -5.858879 -1.399643 1.987072
H -7.618821 -1.233835 1.767106
H -6.879977 -2.852229 1.875961
C 2.850255 2.563846 2.771332
H 3.620766 3.161542 2.253738
H 2.026717 3.240391 3.044327
H 3.298439 2.111759 3.667823
C 4.453460 0.681868 -2.602009
H 4.281945 0.080253 -3.511760
H 4.394671 1.743921 -2.882579
H 5.449652 0.438285 -2.205228
C 0.213442 -3.272866 -2.470513
H -0.839225 -3.509103 -2.239919
H 0.248597 -2.900267 -3.506589
H 0.822912 -4.181959 -2.371073
C -1.476772 -1.301358 2.839857
H -1.483638 -0.525301 3.625065
H -2.466020 -1.297810 2.356697
H -1.277797 -2.275995 3.307741

E(RPBE-PBE) = -1613.42094555
No imaginary frequency
Zero-point correction= 0.400408
Sum of electronic and thermal Enthalpies= -1612.984787
Sum of electronic and thermal Free Energies= -1613.092048
Gsolv= -1613.505005

CO-K$_C$

C 1.482056 1.574409 -0.171915
O 0.668774 1.016375 0.647536
N 1.638937 2.867138 -0.456440
O 0.566251 3.567646 0.275659
S -0.457065 4.437118 -0.714261
C 0.618507 5.803326 -1.183963
H 1.519329 5.348124 -1.623574
H 0.086366 6.421410 -1.921649
H 0.868469 6.381406 -0.283484
O -0.846355 3.704963 -1.927484
O -1.475942 4.879449 0.276531
O 2.435605 0.838095 -0.847088
C 2.823387 -0.382507 -0.201285
H 1.948865 -1.044034 -0.076125
C 3.738208 -1.107701 -1.235785
Cl 5.216857 -0.162903 -1.630060
Cl 4.231024 -2.707724 -0.546197
Cl 2.819099 -1.405808 -2.758244
C 3.471283 -0.135247 1.152751
C 3.210179 -1.018243 2.216889
C 4.285286 0.991213 1.374940
C 3.778265 -0.797123 3.478118
H 2.544920 -1.872002 2.052246
C 4.849195 1.212814 2.639334
H 4.457385 1.700818 0.561410
C 4.603701 0.317532 3.691394

H 3.570270 -1.493155 4.297829
H 5.484317 2.090053 2.801870
H 5.049079 0.491587 4.676789
Rh -1.033893 -0.376335 0.180208
Rh -2.932973 -1.796690 -0.251030
O -2.280318 1.255037 -0.083169
C -3.506645 1.041340 -0.433777
O -4.064136 -0.103700 -0.545333
C -2.590163 -1.085454 2.549952
O -1.589144 -0.343226 2.200613
O -3.323245 -1.780699 1.769556
O 0.082936 -2.084376 0.449461
C -0.488277 -3.228913 0.294126
O -1.722773 -3.425417 0.007515
O -2.451475 -1.759498 -2.235395
C -1.414537 -1.087784 -2.590959
O -0.624298 -0.437630 -1.812283
C -2.896783 -1.165137 4.031350
H -2.681461 -0.208657 4.533429
H -3.945730 -1.452866 4.192182
H -2.250910 -1.934084 4.490896
C 0.379265 -4.450766 0.499979
H 0.490448 -4.642363 1.581964
H -0.084932 -5.331851 0.034352
H 1.383906 -4.279289 0.082690
C -1.100641 -1.024076 -4.067745
H -1.576970 -1.860341 -4.599383
H -1.487712 -0.074622 -4.477451
H -0.011071 -1.033374 -4.222666
C -4.338767 2.259546 -0.766593
H -5.355453 2.154402 -0.356791
H -3.857375 3.179233 -0.402709
H -4.427305 2.333476 -1.864616
K -1.070622 2.401487 2.060512

Egas = -4328.35886658
No imaginary frequency
Zero-point correction= 0.390988
Sum of electronic and thermal Enthalpies= -4327.920683
Sum of electronic and thermal Free Energies= -4328.054430
Gsolv= -4328.770941

TSCO-K$_C$

C	1.535987	-0.253213	-0.602659
O	1.171278	-1.272411	0.021972
N	0.653707	0.728959	-0.836929
O	1.209142	1.853307	-1.546377
S	1.243071	3.236895	-0.580763
C	-0.424503	3.870058	-0.828867
H	-1.136549	3.096573	-0.506478
H	-0.525648	4.786417	-0.227416
H	-0.540941	4.090414	-1.899461
O	1.386119	2.857223	0.852007
O	2.218179	4.136967	-1.214296
O	2.857700	-0.049757	-1.005166
C	3.759146	-0.934411	-0.334204
H	3.357578	-1.962669	-0.363349
C	5.059820	-0.965508	-1.184403
Cl	5.825822	0.651290	-1.334693
Cl	6.222610	-2.102714	-0.387881
Cl	4.670897	-1.588164	-2.827495
C	3.928983	-0.514086	1.123112
C	3.893218	-1.487140	2.140596
C	3.998838	0.847667	1.478825
C	3.929864	-1.110956	3.491361
H	3.819691	-2.545419	1.869555
C	4.037430	1.224912	2.831189
H	3.993546	1.614521	0.700900
C	4.000361	0.248787	3.841365
H	3.902573	-1.879673	4.270834
H	4.094402	2.287307	3.090711
H	4.034649	0.545010	4.895514
Rh	-1.704766	-0.129828	-0.226233
Rh	-4.018065	-0.703680	0.144359
C	-3.579513	1.155083	-2.018309
O	-2.320137	1.069609	-1.770143
O	-4.522490	0.568256	-1.374610
O	-1.610815	-1.704540	-1.523744
C	-2.669754	-2.394895	-1.745635
O	-3.824011	-2.221511	-1.201427
O	-1.924874	1.425296	1.145757

C	-3.085072	1.574273	1.698796
O	-4.121658	0.860725	1.481679
O	-3.468657	-1.987912	1.636728
C	-2.233726	-2.021314	1.973171
O	-1.282974	-1.329420	1.447959
C	-1.844469	-2.983069	3.076405
H	-2.738111	-3.395083	3.565646
H	-1.251314	-3.807233	2.644270
H	-1.208398	-2.478366	3.822739
C	-3.225666	2.690888	2.713320
H	-2.359446	3.367838	2.679533
H	-4.152051	3.254889	2.520803
H	-3.312487	2.260730	3.726459
C	-2.550582	-3.504280	-2.766151
H	-2.672288	-3.080549	-3.778447
H	-1.551574	-3.962447	-2.711158
H	-3.333545	-4.259814	-2.606672
C	-3.981447	2.005136	-3.203042
H	-3.256246	2.817332	-3.360441
H	-3.988564	1.375649	-4.110265
H	-4.993760	2.410564	-3.058689
K	0.696017	0.495662	2.068931

Egas = -4328.35470162
One imaginary frequency of -55.3317
Zero-point correction= 0.390686
Sum of electronic and thermal Enthalpies= -4327.917444
Sum of electronic and thermal Free Energies= -4328.049775
Gsolv= -4328.767775

NRO-K_C

C 1.403085 1.282080 -1.311424
O 2.022396 1.802642 -2.228527
N 0.100524 1.419026 -0.904194
O -0.521158 2.368422 -1.777945
S -0.734799 3.896234 -1.054420
C -2.513768 3.858583 -0.765816
H -2.725151 3.038770 -0.064585
H -2.803029 4.837384 -0.353377
H -3.005116 3.680733 -1.732822
O -0.045950 3.928320 0.264603
O -0.435646 4.902925 -2.082625
O 2.033786 0.428159 -0.354414
C 3.409979 0.176320 -0.605043
H 3.723335 0.787266 -1.474446
C 4.204076 0.754377 0.615086
Cl 3.699919 -0.042229 2.174835
Cl 5.966355 0.537328 0.399109
Cl 3.857728 2.533572 0.765654
C 3.656200 -1.298761 -0.869774
C 4.703337 -1.696583 -1.720826
C 2.818163 -2.276524 -0.302271
C 4.930638 -3.055217 -1.978023
H 5.339714 -0.939051 -2.189787
C 3.047201 -3.635089 -0.561571
H 1.970921 -1.958140 0.310298
C 4.107064 -4.028259 -1.393478
H 5.747174 -3.351849 -2.644054
H 2.391655 -4.390443 -0.114328
H 4.283313 -5.089919 -1.595284
Rh -1.285349 -0.100307 -0.161138
Rh -2.898639 -1.775820 0.545640
O -0.038242 -0.819874 1.373184
C -0.442058 -1.832358 2.071004
O -1.556516 -2.440512 1.944362
O -2.672306 0.504847 -1.551407
C -3.798404 -0.113875 -1.622801
O -4.183214 -1.089421 -0.884538
O -2.085807 1.188272 1.285099
C -3.088875 0.753440 1.983143
O -3.637426 -0.392660 1.885700
O -0.570795 -1.475999 -1.493436
C -1.115503 -2.640434 -1.542793
O -2.093214 -3.059074 -0.821830
C -3.645000 1.691671 3.036328
H -3.243401 1.412133 4.026755
H -3.368890 2.735606 2.825043
H -4.739930 1.591396 3.087937
C -4.775992 0.383900 -2.666353
H -5.480241 1.095722 -2.200360
H -4.243142 0.897481 -3.479870

H -5.363745 -0.457352 -3.063624
C -0.532015 -3.600755 -2.555229
H -0.642337 -3.179834 -3.568621
H 0.548201 -3.722387 -2.368098
H -1.038260 -4.574699 -2.500312
C 0.503722 -2.333450 3.144356
H 1.510657 -2.489896 2.723677
H 0.595813 -1.581082 3.947892
H 0.126450 -3.269909 3.578516
K 0.678009 1.815300 1.77793

Egas = -4328.37205917
No imaginary frequency
Zero-point correction= 0.391580
Sum of electronic and thermal Enthalpies= -4327.933806
Sum of electronic and thermal Free Energies= -4328.063823
Gsolv= -4328.776075

TSNR-K$_C$

C	-1.389556	1.027654	-1.233723
O	-1.764164	1.821755	-2.085584
N	-0.363548	1.042612	-0.341662
O	0.200100	2.960871	-0.449934
S	1.372231	3.654863	-1.268319
C	0.951657	3.323943	-2.990621
H	-0.093411	3.615898	-3.157360
H	1.641829	3.909618	-3.616456
H	1.078796	2.246765	-3.168590
O	1.194517	5.109098	-0.995077
O	2.715200	3.072069	-1.015449
O	-2.167206	-0.131285	-0.893701
C	-3.535998	0.209041	-0.684420
H	-3.852963	0.933265	-1.458849
C	-4.364048	-1.079172	-0.970514
Cl	-3.925143	-2.433687	0.135516
Cl	-6.117135	-0.693171	-0.757306
Cl	-4.082959	-1.599421	-2.668024
C	-3.732840	0.820356	0.698967
C	-4.561794	1.944574	0.862227
C	-3.019989	0.325035	1.809960
C	-4.694062	2.560309	2.115857
H	-5.102901	2.345323	-0.001323
C	-3.161425	0.935146	3.066820
H	-2.338465	-0.519566	1.679081
C	-3.997782	2.055036	3.224435
H	-5.342327	3.435456	2.225651
H	-2.631020	0.519905	3.933059
H	-4.112370	2.524685	4.207403
Rh	1.114054	-0.253128	-0.009678
Rh	2.894115	-1.817653	0.464898
O	2.076236	0.170034	-1.760697
C	3.181978	-0.428822	-2.033952
O	3.752600	-1.327526	-1.318092
C	0.712535	-2.948597	-1.021889
O	0.186257	-1.774652	-1.030745
O	1.807907	-3.288323	-0.445239
O	0.254441	-0.796927	1.822009
C	0.867693	-1.685361	2.534369
O	1.973955	-2.253318	2.247793
O	3.958367	-0.281459	1.308528
C	3.391371	0.855610	1.448390
O	2.167742	1.126396	1.134399
C	4.203501	2.001925	1.999001
H	4.227157	2.796863	1.233816
H	3.721138	2.420344	2.899336
H	5.221889	1.673463	2.248448
C	0.183261	-2.116422	3.814629
H	-0.545270	-2.912594	3.580375
H	0.919238	-2.519560	4.525119
H	-0.369798	-1.279479	4.269110
C	3.865211	-0.002961	-3.311155
H	3.159579	-0.062619	-4.155695
H	4.175905	1.050919	-3.209295
H	4.743607	-0.633479	-3.507189
C	-0.037594	-4.015306	-1.786129
H	0.179724	-3.907583	-2.863161
H	0.282330	-5.015414	-1.460117
H	-1.122402	-3.886849	-1.649348
K	-0.184739	2.267087	2.009132

Egas = -4328.33736557
One imaginary frequency of -87.5260
Zero-point correction= 0.389344
Sum of electronic and thermal Enthalpies= -4327.901439
Sum of electronic and thermal Free Energies= -4328.032429
Gsolv= -4328.765621

[1]NR$_C$

Rh 1.363530 0.507169 0.123796
Rh 3.517607 -0.560622 -0.124130
O 2.237038 1.799341 1.481899
O 4.272366 0.764561 1.240273
O 0.608035 -0.836976 -1.233148
O 2.658172 -1.835186 -1.486841
O 1.017140 -0.857081 1.626082
O 3.054673 -1.853778 1.368272
O 1.956462 1.729139 -1.421719
O 3.995401 0.721625 -1.620731
C 3.486899 1.646295 1.744720
C 1.905747 -1.737192 1.925915
C 1.410636 -1.702261 -1.750092
C 3.117732 1.595134 -1.956373
C 3.460784 2.560406 -3.064030
H 3.527236 3.581752 -2.652862
H 4.419387 2.285220 -3.525574
C 4.072578 2.595655 2.765419
H 3.693743 2.330713 3.767405
H 5.169683 2.531277 2.764673
H 3.746758 3.625445 2.550872
C 1.542890 -2.709482 3.020941
H 0.713063 -3.351488 2.680232
H 2.409994 -3.333121 3.279497
H 1.189963 -2.159227 3.907806
C 0.815201 -2.623807 -2.789002
H 0.837927 -2.118913 -3.770643
H 1.403376 -3.550205 -2.858408
H -0.235421 -2.844295 -2.548025
N -0.304815 1.406606 0.331057
C -1.401490 1.051400 -0.393817
O -1.592922 1.510777 -1.526977
O -2.286312 0.254516 0.289310
C -3.533521 0.019845 -0.394131
H -3.350415 -0.007797 -1.482718
C -3.957860 -1.430395 -0.000748
Cl -5.485586 -1.841823 -0.865928
Cl -4.210277 -1.605149 1.768477
Cl -2.674465 -2.590494 -0.524826
C -4.547643 1.102231 -0.073778
C -5.436218 1.538470 -1.073113
C -4.587595 1.703751 1.197847
C -6.370852 2.544907 -0.799855
H -5.390793 1.090402 -2.071299
C -5.520284 2.713904 1.467108
H -3.875600 1.390538 1.966083
C -6.416838 3.132428 0.472903
H -7.055908 2.877658 -1.586192
H -5.541970 3.179498 2.457660
H -7.142666 3.924025 0.685904

E(RPBE-PBE) = -3065.32222861
No imaginary frequency
Zero-point correction= 0.338789
Sum of electronic and thermal Enthalpies= -3064.944961
Sum of electronic and thermal Free Energies= -3065.059917
Gsolv= -3065.584703

S5-5. Triplet Rh-Nitrenes species of **A**, **B** & **C** (Table 2).

$^{3}NR_{A}$

Rh 0.882231 0.760721 -0.180260
Rh 1.942085 -1.359917 0.278493
O -0.528673 0.210498 1.218015
O 0.466880 -1.812866 1.610046
O 2.397587 1.140058 -1.515233
O 3.399506 -0.862868 -1.055182
O -0.161559 -0.213030 -1.682378
O 0.866438 -2.213239 -1.234611
O 2.034557 1.555310 1.335878
O 3.011573 -0.470384 1.776033
C -0.445604 -0.930692 1.805655
C 0.059887 -1.463327 -1.892166
C 2.831190 0.782697 1.986421
N -0.091571 2.391398 -0.455186
C -1.348196 2.860621 -0.188847
O -1.547318 3.950430 0.360495
O -2.344646 2.030072 -0.625860
C -4.671002 1.569840 -1.056369
H -3.776243 3.542517 -0.764093
H -3.850643 2.556468 0.723383
H -5.665241 2.051317 -1.011408
H -4.395320 1.507837 -2.125348
C -4.750560 0.182593 -0.449908
C -3.744779 -0.774274 -0.694642
C -5.828589 -0.177144 0.380688
C -3.822315 -2.055270 -0.128582
H -2.890324 -0.498578 -1.320466
C -5.907877 -1.455993 0.951762
H -6.621554 0.554172 0.578011
C -4.904943 -2.402020 0.696668
H -3.035303 -2.790485 -0.334430
H -6.758973 -1.715135 1.590415
H -4.968035 -3.404887 1.132015
C -1.535265 -1.251152 2.798675
H -1.709109 -0.385301 3.456849
H -2.473837 -1.448609 2.251818
H -1.262411 -2.135289 3.392088
C -0.720868 -2.101862 -3.018925
H -0.336604 -3.110769 -3.224619
H -1.786998 -2.167873 -2.741362
H -0.654353 -1.475987 -3.923004
C 4.443668 0.622307 -2.616758
H 4.024831 1.042834 -3.544247
H 5.080271 1.398528 -2.158361
H 5.056126 -0.263095 -2.837828
C 3.607533 1.424543 3.112439
H 4.034782 2.382306 2.776511
H 2.920479 1.644142 3.947529
H 4.401742 0.750939 3.463636

Egas = -1687.14785959
No imaginary frequency
Zero-point correction= 0.368111
Sum of electronic and thermal Enthalpies= -1686.745072
Sum of electronic and thermal Free Energies= -1686.850350
Gsolv= -1687.279599

$^{3}NR_{B}$

Rh 0.838018 0.685920 -0.351916
Rh 1.936001 -1.256323 0.584341
O -0.624043 0.471778 1.077498
O 0.413043 -1.377231 1.955978
O 2.369969 0.804112 -1.734793
O 3.404026 -1.037308 -0.830006
O -0.148992 -0.664953 -1.577531
O 0.915517 -2.476544 -0.676285
O 1.940701 1.792825 0.973365
O 2.968539 -0.048134 1.847966
C -0.530277 -0.509894 1.908911
C 0.107622 -1.922628 -1.503235
C 3.316809 -0.063778 -1.663333
C 2.747632 1.213198 1.790978
N -0.030602 2.161441 -1.195179
C -1.217107 2.622325 -0.695157

O -1.209870 3.625081 0.035421
O -2.323478 1.971212 -1.124039
C -3.584338 2.505847 -0.641747
C -4.699933 1.577709 -1.130687
H -3.715038 3.526727 -1.042343
H -3.555948 2.564835 0.459588
H -5.657077 2.104453 -0.961863
H -4.591727 1.461924 -2.225222
C -4.742434 0.221297 -0.452615
C -3.758356 -0.755713 -0.710153
C -5.769571 -0.087877 0.459360
C -3.807934 -2.004174 -0.072955
H -2.942262 -0.525292 -1.401755
C -5.821879 -1.335987 1.097671
H -6.544683 0.659197 0.668115
C -4.840084 -2.301030 0.832185
H -3.038185 -2.754681 -0.286656
H -6.634532 -1.555824 1.798103
H -4.880667 -3.280145 1.320969
C -1.661772 -0.650009 2.898974
H -1.924650 0.335185 3.314763
H -2.551290 -1.039431 2.372747
H -1.381148 -1.344834 3.703190
C -0.595911 -2.810196 -2.503151
H -1.663596 -2.547040 -2.566580
H -0.155910 -2.646592 -3.502096
H -0.476420 -3.866999 -2.225613
C 3.463877 2.104874 2.774959
H 3.739251 3.057171 2.297402
H 2.783549 2.333327 3.613825
H 4.354526 1.595495 3.169913
C 4.452629 0.108719 -2.646799
H 4.075991 0.512968 -3.598091
H 5.176437 0.833143 -2.233913
H 4.969621 -0.848362 -2.807165

Egas = -1613.41996906
No imaginary frequency
Zero-point correction= 0.400195
Sum of electronic and thermal Enthalpies= -1612.983953
Sum of electronic and thermal Free Energies= -1613.093124
Gsolv= -1613.506003

^{3}NR$_C$

Rh 1.465638 0.231926 0.194777
Rh 3.809512 -0.131171 -0.234903
O 2.021286 1.671870 1.556385
O 4.240758 1.306163 1.144706
O 1.109273 -1.244523 -1.194093
O 3.337467 -1.558728 -1.608928
O 1.610292 -1.205053 1.671119
O 3.833527 -1.541810 1.239670
O 1.522114 1.644302 -1.301431
O 3.748758 1.285805 -1.702190
C 3.273683 1.897689 1.745951
C 2.742382 -1.781919 1.871208
C 2.092552 -1.808149 -1.799604
C 2.624441 1.863519 -1.925887
C 2.569226 2.896323 -3.026399
H 1.872162 2.556630 -3.810445
H 2.175901 3.845164 -2.626949
H 3.568141 3.052093 -3.456888
C 3.620594 2.938203 2.783242
H 3.426562 2.527461 3.788976
H 4.680952 3.216608 2.705476
H 2.979141 3.824280 2.657434
C 2.770514 -2.850954 2.937667
H 2.259007 -3.753001 2.560075
H 3.807878 -3.106247 3.195959
H 2.223077 -2.508879 3.829597
C 1.730648 -2.854925 -2.825368
H 1.107559 -2.397111 -3.611299
H 2.638523 -3.286912 -3.268699
H 1.128636 -3.646823 -2.350425
N -0.419106 0.447562 0.436543
C -1.500266 0.288929 -0.370296
O -1.494560 0.296920 -1.606286
O -2.647290 0.137280 0.397600

C -3.867743 -0.047961 -0.332236
H -3.633260 -0.141005 -1.407532
C -4.434853 -1.442554 0.097197
C -4.798725 1.126066 -0.095672
C -5.630707 1.577129 -1.136440
C -6.490499 2.664921 -0.935997
H -5.603014 1.073536 -2.108417
C -5.676617 2.884004 1.339892
H -4.149354 1.465741 1.942218
C -6.517912 3.318257 0.304639
H -7.133267 3.006698 -1.753627
H -5.684294 3.398468 2.306320
H -7.186589 4.171053 0.461407
Cl -3.217391 -2.717143 -0.296399
Cl -5.942211 -1.779709 -0.837104
Cl -4.799551 -1.507784 1.855497

Egas = -3065.32351041
No imaginary frequency
Zero-point correction= 0.338952
Sum of electronic and thermal Enthalpies= -3064.946147
Sum of electronic and thermal Free Energies= -3065.061932
Gsolv= -3065.587005

S5-6. Rh-Nitrenes C-H insertion mechanisms for **A** & **C** (Figure 6).

$^{1}TS_{A}$

Rh -0.481391 0.361257 -0.198446
Rh -2.238797 -1.200881 0.353235
O 0.029314 -0.883769 -1.768356
O -1.665801 -2.343646 -1.248681
O -1.059075 1.499840 1.410417
O -2.738217 0.020567 1.924164
O 0.749725 -0.710308 1.039109
O -0.903093 -2.206869 1.528764
O -1.881433 1.289616 -1.389742
O -3.538970 -0.184522 -0.825473
C -0.670261 -1.946613 -1.955948
C 0.292364 -1.762244 1.627439
C -2.063896 1.097176 2.107403
C -3.081962 0.841851 -1.450328
N 0.942788 1.603176 -0.777488
C 0.934223 2.946441 -0.489400
O -0.038245 3.663303 -0.727991
O 2.093609 3.531104 -0.000851
C 3.311249 2.788309 -0.145785
C 3.142561 1.372544 0.390768
H 4.075546 3.350064 0.418914
H 3.606076 2.776534 -1.212499
H 2.787350 1.376860 1.433636
H 2.127191 1.091231 -0.260030
C 4.184468 0.355907 0.133416
C 4.550187 -0.547829 1.160711
C 4.819459 0.228832 -1.126335
C 5.525901 -1.526054 0.947049
H 4.061456 -0.462514 2.137636
C 5.792494 -0.753095 -1.340098
H 4.535119 0.893257 -1.948547
C 6.151624 -1.632688 -0.306023
H 5.802833 -2.206693 1.758894
H 6.271157 -0.836979 -2.321127
H 6.912865 -2.400573 -0.477133
C -4.029541 1.588928 -2.359153
H -3.882343 2.673652 -2.242725
H -3.804462 1.332289 -3.408761
H -5.070170 1.312173 -2.137892
C -0.289061 -2.795249 -3.149207
H -0.638124 -2.299685 -4.071639
H 0.806259 -2.885651 -3.215213
H -0.754082 -3.788735 -3.078950
C 1.253875 -2.507380 2.523316
H 2.250082 -2.552598 2.055845
H 1.352248 -1.968131 3.481812
H 0.877116 -3.520054 2.725688
C -2.499421 2.006999 3.233653
H -1.621706 2.454448 3.724317
H -3.102763 2.830906 2.814336
H -3.110768 1.453499 3.960589

Egas = -1687.13463738
One imaginary frequency of -468.6216
Zero-point correction= 0.363682
Sum of electronic and thermal Enthalpies= -1686.736899
Sum of electronic and thermal Free Energies= -1686.838635
Gsolv= -1687.272794

$^{3}TS_{A}$

Rh 0.483457 0.393599 0.013312
Rh 2.241000 -1.255186 -0.017823
O -0.334206 -0.630795 1.606441
O 1.352404 -2.175447 1.573071
O 1.432959 1.279296 -1.580904
O 3.090289 -0.299929 -1.603761
O -0.576140 -0.816059 -1.277086
O 1.091363 -2.386666 -1.282047
O 1.645616 1.475966 1.317490
O 3.331112 -0.077957 1.244984
C 0.260975 -1.685974 2.037533
C -0.053246 -1.933536 -1.642625
C 2.522159 0.769496 -2.033157
C 2.801614 1.022048 1.647000
N -0.961117 1.708215 -0.003070
C -0.986106 3.079748 0.009593
O 0.007405 3.805621 -0.041908
O -2.242850 3.666092 0.051698
C -3.376484 2.827867 0.366334
C -3.431022 1.614435 -0.551728
H -4.254840 3.485888 0.249710
H -3.308842 2.522740 1.427252
H -3.456384 1.908202 -1.614835
H -2.205086 1.297197 -0.384220
C -4.283043 0.455147 -0.242569
C -4.767800 -0.352898 -1.303800
C -4.599719 0.061545 1.082993
C -5.536484 -1.492962 -1.055486
H -4.532035 -0.065602 -2.334893
C -5.372178 -1.078259 1.329204
H -4.229831 0.648547 1.929358
C -5.845164 -1.862607 0.264562
H -5.904013 -2.093978 -1.894068
H -5.607321 -1.358305 2.361583
H -6.449311 -2.754041 0.461519
C -0.397238 -2.406549 3.190765
H -0.547861 -1.707691 4.029663
H -1.391835 -2.766627 2.878464
H 0.219957 -3.256045 3.514763
C -0.865427 -2.768381 -2.605957
H -1.934176 -2.710404 -2.348041
H -0.741432 -2.365416 -3.626326
H -0.519236 -3.811720 -2.594444
C 3.594800 1.860103 2.622638
H 3.405766 2.928977 2.443383
H 3.268282 1.624181 3.650592
H 4.667273 1.633552 2.535792
C 3.196532 1.519401 -3.157105
H 2.445130 1.886045 -3.873006
H 3.715014 2.399840 -2.739573
H 3.931360 0.875819 -3.661176

Egas = -1687.12907448
One imaginary frequency of -1189.3416
Zero-point correction= 0.362852
Sum of electronic and thermal Enthalpies= -1686.732036
Sum of electronic and thermal Free Energies= -1686.835743
Gsolv= -1687.269597

INT_{A}

Rh 0.423241 0.396721 0.031576
Rh 2.208446 -1.219829 0.003413
O -0.305184 -0.517886 1.727855
O 1.388120 -2.061184 1.688876

O 1.229311 1.200260 -1.680469
O 2.950519 -0.317611 -1.670783
O -0.671667 -0.915336 -1.111385
O 1.037165 -2.431860 -1.136282
O 1.641369 1.582031 1.174281
O 3.329070 0.039684 1.138704
C 0.322402 -1.546465 2.182488
C -0.144544 -2.036776 -1.450342
C 2.312402 0.693336 -2.147598
C 2.817980 1.169693 1.480217
N -1.136274 1.668057 0.187956
C -1.095019 3.032799 -0.024235
O -0.115935 3.663988 -0.402328
O -2.293175 3.707881 0.154524
C -3.476573 2.901535 0.345644
C -3.497705 1.715578 -0.561732
H -4.307634 3.600925 0.129561
H -3.567266 2.618646 1.414950
H -3.126932 1.892693 -1.577454
H -1.800474 1.326583 0.890376
C -4.112798 0.464715 -0.293157
C -4.154027 -0.533632 -1.322112
C -4.694675 0.117047 0.970006
C -4.739708 -1.777523 -1.105113
H -3.710866 -0.294912 -2.294847
C -5.271261 -1.135389 1.177700
H -4.697839 0.845677 1.787098
C -5.302646 -2.094555 0.147659
H -4.768645 -2.512319 -1.917674
H -5.710778 -1.369858 2.153335
H -5.763684 -3.072631 0.316537
C 3.656111 2.087791 2.336758
H 3.427761 3.137376 2.099103
H 3.410978 1.916522 3.399574
H 4.725225 1.878263 2.187396
C -0.984214 -2.952232 -2.308258
H -2.032991 -2.916934 -1.975191
H -0.948304 -2.602394 -3.354798
H -0.595406 -3.979666 -2.265637
C -0.266731 -2.201154 3.410974
H -0.387933 -1.452215 4.210562
H -1.269336 -2.594236 3.172570
H 0.378983 -3.019959 3.757310
C 2.900835 1.375098 -3.360873
H 2.099510 1.659834 -4.059769
H 3.405457 2.302832 -3.040166
H 3.632443 0.720306 -3.855105

Egas = -1687.13973493
No imaginary frequency
Zero-point correction= 0.366698
Sum of electronic and thermal Enthalpies= -1686.738218
Sum of electronic and thermal Free Energies= -1686.843246
Gsolv= -1687.278613

TSr_A

Rh 0.405389 0.396525 0.030440
Rh 2.198401 -1.217357 0.026370
O -0.332421 -0.507437 1.728399
O 1.364883 -2.047979 1.712524
O 1.226279 1.195274 -1.678625
O 2.949516 -0.321402 -1.649522
O -0.671531 -0.927479 -1.119457
O 1.040102 -2.438614 -1.113352
O 1.605790 1.586670 1.184933
O 3.301558 0.054890 1.162238
C 0.297369 -1.528339 2.197397
C -0.140675 -2.051191 -1.442032
C 2.304178 0.676696 -2.144223
C 2.788714 1.189455 1.486033
N -1.177866 1.666195 0.164683
C -1.094515 3.039271 -0.034400
O -0.101429 3.649638 -0.405279
O -2.283578 3.729231 0.133204
C -3.469120 2.916408 0.286848
C -3.420489 1.700560 -0.579883
H -4.296112 3.595080 0.001346
H -3.612086 2.666928 1.357973

H -3.060636 1.865976 -1.601541
H -1.760818 1.367066 0.955551
C -4.043009 0.453432 -0.301286
C -4.081715 -0.554919 -1.319763
C -4.630817 0.119596 0.962282
C -4.674412 -1.794225 -1.094273
H -3.633305 -0.327201 -2.292673
C -5.213755 -1.128340 1.179371
H -4.633650 0.854716 1.773592
C -5.245147 -2.097182 0.158267
H -4.703484 -2.535961 -1.900589
H -5.658403 -1.351579 2.155352
H -5.711910 -3.071336 0.333961
C 2.849769 1.288405 -3.414256
H 2.367057 0.807837 -4.283300
H 2.618238 2.363257 -3.445526
H 3.934403 1.120581 -3.484028
C -0.970460 -2.980516 -2.294414
H -0.561624 -4.000340 -2.258858
H -2.015939 -2.966463 -1.949364
H -0.954515 -2.626350 -3.339931
C -0.288518 -2.163451 3.437793
H -0.334391 -1.417788 4.248799
H -1.321115 -2.491005 3.232395
H 0.320081 -3.021507 3.754942
C 3.650909 2.154412 2.262124
H 4.482262 1.623591 2.747424
H 4.063270 2.904255 1.564693
H 3.043969 2.691581 3.006555

Egas = -1687.13607558
One imaginary frequency of -41.0337
Zero-point correction= 0.366361
Sum of electronic and thermal Enthalpies= -1686.735548
Sum of electronic and thermal Free Energies= -1686.838820
Gsolv= -1687.282311

Oxazolidinone from A • $Rh_2(O_2CCH_3)_4$

Rh -0.850392 0.527053 -0.236264
Rh -2.510174 -1.075329 0.446300
O -0.173141 -0.853230 -1.608388
O -1.768889 -2.370521 -0.960163
O -1.601956 1.819780 1.174573
O -3.191051 0.290875 1.813121
O 0.412814 -0.306266 1.195099
O -1.180111 -1.816682 1.829722
O -2.196360 1.242676 -1.599813
O -3.773203 -0.294140 -0.951444
C -0.770865 -1.989958 -1.673093
C -0.018447 -1.295199 1.908023
C -2.598294 1.428724 1.886291
C -3.353297 0.687721 -1.667354
N 2.513676 1.063646 -0.027503
C 1.831432 2.002415 -0.740788
O 0.615282 2.068032 -0.973723
O 2.692648 2.945842 -1.226866
C 4.040370 2.509872 -0.925753
C 3.891479 1.470612 0.220784
H 4.636161 3.390370 -0.645633
H 4.467611 2.039665 -1.829491
H 3.978756 1.976138 1.205298
H 1.981180 0.420655 0.577511
C 4.898107 0.336908 0.157352
C 5.982664 0.305671 1.051100
C 4.776762 -0.678048 -0.811063
C 6.939069 -0.717386 0.974631
H 6.075869 1.086372 1.815606
C 5.728678 -1.702990 -0.883768
H 3.921573 -0.667659 -1.495886
C 6.813687 -1.723869 0.006671
H 7.778107 -0.731339 1.677812
H 5.621583 -2.490779 -1.636676
H 7.555659 -2.526898 -0.049882
C 0.942098 -1.862356 2.930425
H 1.900647 -2.119288 2.449516
H 1.151667 -1.103092 3.703365
H 0.512984 -2.755201 3.405914
C -0.222909 -2.989562 -2.669892

H 0.224219 -2.468305 -3.529378
H 0.564289 -3.593572 -2.184990
H -1.019353 -3.670725 -3.003946
C -4.305692 1.249279 -2.700208
H -4.421535 2.334770 -2.547752
H -3.883729 1.103029 -3.708721
H -5.284159 0.753408 -2.633779
C -3.102474 2.394445 2.937421
H -2.572463 2.206866 3.887921
H -2.903764 3.432116 2.631504
H -4.178025 2.241777 3.110987

E(RPBE-PBE) = -1687.23630189
No imaginary frequency
Zero-point correction= 0.372062
Sum of electronic and thermal Enthalpies= -1686.830321
Sum of electronic and thermal Free Energies= -1686.933083
Gsolv= -1687.365059

Ethyl Benzene

C 2.332917 0.000136 0.238433
C 1.641173 -1.211435 0.095902
C 0.268237 -1.208281 -0.186306
C -0.440101 -0.000127 -0.331905
C 0.268043 1.208162 -0.186470
C 1.640978 1.211578 0.095732
H 3.406300 0.000235 0.454889
H 2.173348 -2.163200 0.199633
H -0.265767 -2.159375 -0.301380
H -0.266115 2.159154 -0.301678
H 2.172995 2.163445 0.199332
C -1.932716 -0.000256 -0.593292
H -2.202801 0.885397 -1.197687
H -2.202756 -0.886343 -1.197067
C -2.761889 0.000186 0.705234
H -2.535763 0.890466 1.316925
H -3.843602 0.000064 0.485494
H -2.535688 -0.889633 1.317567

E(RPBE-PBE) = -310.452164483
No imaginary frequency
Zero-point correction= 0.153437
Sum of electronic and thermal Enthalpies= -310.290294
Sum of electronic and thermal Free Energies= -310.330921
Gsolv= -310.4126772

$^{1}TS_{C}$

C	0.942763	-0.476590	-1.398980
O	0.780518	-1.489748	-2.085500
N	-0.007866	0.504859	-1.327309
O	2.111910	-0.198846	-0.702218
C	3.101062	-1.238295	-0.759494
H	2.786130	-1.978491	-1.516977
C	4.411206	-0.600790	-1.318845
Cl	5.057977	0.679914	-0.240975
Cl	5.652642	-1.899741	-1.515799
Cl	4.066651	0.117179	-2.940797
C	3.226068	-1.906989	0.597643
C	3.423111	-3.297793	0.672288
C	3.090369	-1.164611	1.786437
C	3.499804	-3.939072	1.916105
H	3.510456	-3.881611	-0.250040
C	3.164057	-1.808384	3.028787
H	2.912167	-0.087148	1.733244
C	3.371451	-3.194867	3.098184
H	3.652837	-5.022256	1.960250
H	3.058030	-1.222894	3.948225
H	3.428848	-3.694664	4.070907
Rh	-1.706528	0.081884	-0.383292
Rh	-3.842892	-0.483223	0.598358
O	-1.348989	1.214626	1.300916
C	-2.244236	1.274448	2.227473
O	-3.379251	0.685888	2.203629

O	-2.282253	-1.109837	-1.959566
C	-3.418022	-1.707105	-1.944312
O	-4.292584	-1.641447	-1.005621
O	-2.615634	1.710577	-1.277075
C	-3.876403	1.879760	-1.078323
O	-4.642177	1.147875	-0.354286
O	-0.910947	-1.546245	0.579017
C	-1.685839	-2.276211	1.302809
O	-2.938639	-2.085613	1.507084
C	0.749976	2.934728	-0.567163
H	-0.225543	3.076596	-0.072725
C	0.780210	3.499582	-1.972938
H	1.685875	3.192505	-2.523283
H	-0.096442	3.138842	-2.533852
H	0.749119	4.605957	-1.954474
C	1.882807	3.172009	0.357953
C	1.765021	2.777017	1.715383
C	3.082805	3.798147	-0.052220
C	2.796801	3.014625	2.626511
H	0.849310	2.264164	2.029480
C	4.111829	4.046159	0.864403
H	3.201875	4.110934	-1.093582
C	3.976190	3.654830	2.205300
H	2.685920	2.703610	3.671018
H	5.026407	4.546644	0.529642
H	4.784744	3.844447	2.918762
H	0.631637	1.689207	-0.761796
C	-3.745894	-2.548291	-3.155255
H	-3.912508	-1.888803	-4.023953
H	-2.892628	-3.201519	-3.396768
H	-4.649470	-3.146751	-2.972681
C	-4.518007	3.045545	-1.798504
H	-3.830804	3.904878	-1.824604
H	-4.729165	2.752523	-2.841788
H	-5.464501	3.322228	-1.312502
C	-1.903347	2.092494	3.451649
H	-1.193372	1.527425	4.080650
H	-1.416501	3.036686	3.161315
H	-2.811114	2.295251	4.037249
C	-1.046289	-3.485891	1.945219
H	-0.980187	-4.294504	1.196347
H	-0.021474	-3.249437	2.271228
H	-1.654830	-3.837114	2.790901

Egas = -3375.76892155
One imaginary frequency of -619.6684
Zero-point correction= 0.488654
Sum of electronic and thermal Enthalpies= -3375.233283
Sum of electronic and thermal Free Energies= -3375.366572
Gsolv= -3375.976744

^3NR_C

C 1.046521 -1.036167 0.317917
O 0.839199 -2.072251 0.952601
N 0.114136 -0.128462 -0.074471
O 2.329241 -0.636541 -0.095426
C 3.387550 -1.519008 0.275060
H 2.945169 -2.445306 0.687210
C 4.097462 -1.943480 -1.052053
Cl 4.815460 -0.525939 -1.911881
Cl 5.408583 -3.126313 -0.684981
Cl 2.895352 -2.733380 -2.141420
C 4.290530 -0.865380 1.306611
C 4.900723 -1.650127 2.302254
C 4.491401 0.527559 1.308208
C 5.719516 -1.055852 3.271609
H 4.729146 -2.731494 2.320736
C 5.306439 1.120135 2.281335
H 3.994160 1.147823 0.558111
C 5.926971 0.331282 3.261303
H 6.187277 -1.677502 4.042033
H 5.447939 2.206090 2.272971
H 6.562088 0.796911 4.022343
Rh -1.846673 -0.147605 0.085209
Rh -4.255746 -0.138687 0.241929
O -1.891221 1.886386 0.464330
C -3.033222 2.447469 0.659083
O -4.176986 1.869061 0.621932

O -1.974027 -2.155085 -0.313334
C -3.128067 -2.719201 -0.334360
O -4.258217 -2.142170 -0.131392
O -2.063204 0.271346 -1.926732
C -3.249933 0.374014 -2.413560
O -4.347786 0.239496 -1.763619
O -1.808424 -0.532654 2.100255
C -2.918355 -0.660397 2.734645
O -4.094750 -0.531765 2.234331
C 1.094286 2.560078 -1.946027
H 0.032758 2.794940 -1.790666
C 1.454887 1.852852 -3.218906
H 2.350630 1.217551 -3.106692
H 0.623780 1.209845 -3.549849
H 1.665702 2.575923 -4.035041
C 2.026945 3.276126 -1.117971
C 1.552738 4.008139 0.012931
C 3.429686 3.291837 -1.375138
C 2.428955 4.713687 0.834137
H 0.480143 3.984542 0.237201
C 4.300760 4.006956 -0.550695
H 3.825162 2.741273 -2.233736
C 3.810024 4.721289 0.557674
H 2.041706 5.263332 1.698414
H 5.373290 4.010950 -0.771904
H 4.497149 5.281354 1.200036
H 0.751551 1.034188 -0.818162
C -3.348625 0.703672 -3.885820
H -3.094321 1.766651 -4.040303
H -2.626633 0.100835 -4.458447
H -4.370375 0.525202 -4.249469
C -3.142893 -4.197038 -0.647503
H -2.942530 -4.342815 -1.722988
H -2.342176 -4.705754 -0.088737
H -4.122569 -4.630812 -0.402201
C -2.817811 -1.020622 4.197961
H -2.667944 -2.110633 4.288366
H -1.947844 -0.524280 4.653503
H -3.743143 -0.746171 4.724573
C -3.012343 3.925130 0.981002
H -4.028116 4.342175 0.935642
H -2.606258 4.070683 1.996826
H -2.351031 4.458293 0.279385

Egas = -3375.73405819.0896
One imaginary frequency of -1697.4574
Zero-point correction= 0.485161
Sum of electronic and thermal Enthalpies= -3375.200795
Sum of electronic and thermal Free Energies= -3375.339825
Gsolv= -3375.952982

INT$_C$

C 1.086637 -0.936682 0.256935
O 0.875467 -1.966639 0.890673
N 0.172934 -0.025094 -0.179665
O 2.377896 -0.524306 -0.129906
C 3.439074 -1.386596 0.277705
H 2.998197 -2.311976 0.692636
C 4.187693 -1.822492 -1.024355
Cl 4.922703 -0.415286 -1.881432
Cl 5.491290 -2.997327 -0.606049
Cl 3.018314 -2.632213 -2.137290
C 4.308270 -0.704876 1.320059
C 4.910739 -1.466121 2.338396
C 4.484802 0.691375 1.307092
C 5.699004 -0.845126 3.316317
H 4.757697 -2.550013 2.367542
C 5.269051 1.310396 2.289096
H 3.993356 1.293511 0.538543
C 5.882698 0.545240 3.291880
H 6.161577 -1.448521 4.104186
H 5.392183 2.398382 2.268971
H 6.493988 1.031720 4.059370
Rh -1.814441 -0.132399 0.052261
Rh -4.209183 -0.208602 0.311203
O -1.915536 1.893953 0.446116
C -3.067297 2.419909 0.679648
O -4.190622 1.802232 0.687764

O -1.881407 -2.140962 -0.357452
C -3.011213 -2.749148 -0.311739
O -4.151453 -2.208548 -0.064498
O -2.127229 0.274487 -1.945684
C -3.335611 0.323942 -2.386664
O -4.398807 0.166721 -1.686122
O -1.674377 -0.515582 2.060339
C -2.749496 -0.675388 2.744468
O -3.950436 -0.593132 2.292103
C 0.797883 2.265379 -1.893835
H -0.258342 2.399803 -1.628763
C 1.109385 1.430574 -3.098239
H 2.028584 0.831364 -2.965591
H 0.279219 0.737834 -3.311003
H 1.262893 2.056474 -4.002877
C 1.744441 3.064337 -1.187456
C 1.324099 3.847576 -0.064870
C 3.128281 3.121694 -1.545793
C 2.226214 4.635078 0.645835
H 0.271588 3.800302 0.237122
C 4.022352 3.919112 -0.830750
H 3.485469 2.535623 -2.397814
C 3.583089 4.681183 0.268809
H 1.877876 5.221172 1.503112
H 5.075501 3.951243 -1.130280
H 4.289441 5.306570 0.824114
H 0.577471 0.797355 -0.665699
C -2.981220 -4.240355 -0.546863
H -2.270977 -4.482259 -1.352094
H -2.625633 -4.740284 0.370726
H -3.986653 -4.611777 -0.790804
C -2.572636 -1.009838 4.206037
H -2.229332 -2.054709 4.296920
H -1.795143 -0.367890 4.648427
H -3.523216 -0.892652 4.745184
C -3.082552 3.898737 0.992108
H -2.648575 4.063656 1.993343
H -2.460628 4.446692 0.266425
H -4.111937 4.283187 0.977605
C -3.506283 0.560531 -3.869014
H -2.764190 1.289800 -4.228284
H -3.333494 -0.387503 -4.407729
H -4.525954 0.909216 -4.085932

Egas = -3375.78784046
No imaginary frequency
Zero-point correction= 0.491667
Sum of electronic and thermal Enthalpies= -3375.248088
Sum of electronic and thermal Free Energies= -3375.386194
Gsolv= -3376.000126

TSr_C

C 1.268648 -0.110388 -0.952511
O 1.139252 -0.889304 -1.891488
N 0.306144 0.693737 -0.403430
O 2.494442 0.075901 -0.282875
C 3.480851 -0.907319 -0.605367
H 3.358419 -1.206660 -1.661899
C 4.862004 -0.191623 -0.518632
Cl 5.163848 0.508424 1.109016
Cl 6.165281 -1.378754 -0.907055
Cl 4.921045 1.135451 -1.746036
C 3.326984 -2.131047 0.286838
C 3.518260 -3.416916 -0.249309
C 2.932927 -1.997374 1.631436
C 3.338083 -4.554206 0.549273
H 3.802941 -3.526745 -1.300919
C 2.751078 -3.135186 2.429231
H 2.754615 -1.000664 2.043458
C 2.956217 -4.415800 1.892415
H 3.489926 -5.549605 0.119282
H 2.447985 -3.020066 3.475247
H 2.815666 -5.303483 2.518282
Rh -1.607254 0.054727 -0.207884
Rh -3.798884 -0.832945 0.232756
O -1.419594 0.375486 1.819481
C -2.394640 0.047650 2.592666
O -3.511395 -0.467406 2.223782

O -1.943559 -0.278798 -2.200614
C -3.050474 -0.827500 -2.553651
O -4.009831 -1.168602 -1.767645
O -2.477151 1.908260 -0.395072
C -3.753713 2.011114 -0.258973
O -4.556773 1.044234 -0.000933
O -0.903622 -1.855008 0.033459
C -1.719709 -2.808906 0.301718
O -2.991306 -2.686699 0.454533
C 0.550852 3.155548 -1.334941
H -0.537401 3.040355 -1.347506
C 1.290528 2.786162 -2.580468
H 2.323413 2.452973 -2.379331
H 0.772152 1.969655 -3.111730
H 1.360217 3.640729 -3.286601
C 1.117362 3.749026 -0.175639
C 0.271913 4.092138 0.933689
C 2.519951 4.005220 -0.023133
C 0.791253 4.651831 2.098111
H -0.804007 3.901199 0.843474
C 3.027077 4.565091 1.147735
H 3.203834 3.752475 -0.838651
C 2.174275 4.896469 2.218991
H 0.117803 4.906859 2.924319
H 4.104095 4.746619 1.233532
H 2.581028 5.337445 3.134478
H 0.651260 1.242344 0.394609
C -4.342317 3.391588 -0.424895
H -3.863525 4.088590 0.282512
H -4.133060 3.761941 -1.442343
H -5.427354 3.369994 -0.253279
C -1.125072 -4.190785 0.419213
H -1.004961 -4.614083 -0.593380
H -0.126386 -4.138541 0.879773
H -1.791827 -4.845696 0.998626
C -2.185973 0.267372 4.072789
H -1.629578 -0.590732 4.488880
H -1.585811 1.173646 4.243772
H -3.154220 0.339351 4.588692
C -3.218685 -1.119722 -4.025325
H -2.791196 -0.302310 -4.625505
H -2.663213 -2.041007 -4.272838
H -4.280354 -1.267167 -4.269500

Egas = -3375.78002695
One imaginary frequency of -15.6704
Zero-point correction= 0.491879
Sum of electronic and thermal Enthalpies= -3375.240898
Sum of electronic and thermal Free Energies= -3375.377086
Gsolv= -3375.990985

Amine from C • $Rh_2(O_2CCH_3)_4$

C	-1.274520	-0.364510	-1.572793
O	-1.213249	0.321638	-2.579215
N	-0.274557	-1.314350	-1.228750
O	-2.244947	-0.282124	-0.613059
C	-3.165688	0.815822	-0.781345
H	-2.924631	1.324720	-1.730528
C	-4.596400	0.217362	-0.984619
Cl	-5.182225	-0.670426	0.456358
Cl	-5.729598	1.572848	-1.354414
Cl	-4.576889	-0.909232	-2.401704
C	-3.031063	1.771185	0.388336
C	-3.136740	3.156394	0.163813
C	-2.774783	1.297991	1.689230
C	-3.013431	4.059419	1.228162
H	-3.317253	3.528542	-0.850158
C	-2.656403	2.204370	2.751585
H	-2.647662	0.226655	1.865437
C	-2.779346	3.584073	2.526819
H	-3.098376	5.134681	1.040985
H	-2.467670	1.826773	3.762095
H	-2.686775	4.287897	3.360609
Rh	1.594244	-0.203145	-0.295293
Rh	3.703848	0.797495	0.289543
O	2.295816	-1.979164	0.496519
C	3.478351	-1.995945	1.009705
O	4.281720	-1.002186	1.089834

O	1.032007	1.619558	-1.054024
C	1.865376	2.596407	-1.002917
O	3.055477	2.556751	-0.521621
O	2.408264	-0.740013	-2.111183
C	3.632964	-0.411841	-2.349453
O	4.415085	0.208555	-1.547715
O	0.899207	0.397334	1.550656
C	1.691101	1.046721	2.328635
O	2.913476	1.357470	2.087660
C	-0.614751	-2.714697	-0.749242
H	0.387607	-3.135612	-0.553748
C	-1.251449	-3.494586	-1.908707
H	-2.224798	-3.067021	-2.205114
H	-0.589385	-3.476216	-2.791804
H	-1.405708	-4.549378	-1.626559
C	-1.385218	-2.786092	0.567014
C	-0.756705	-2.386419	1.763792
C	-2.680713	-3.329031	0.640801
C	-1.415650	-2.507639	2.994052
H	0.254716	-1.975717	1.726391
C	-3.335988	-3.464275	1.874129
H	-3.196778	-3.651996	-0.267563
C	-2.708818	-3.049939	3.056164
H	-0.908775	-2.188271	3.911496
H	-4.343156	-3.893055	1.905232
H	-3.219325	-3.155860	4.019250
H	0.295246	-1.413620	-2.082338
C	1.379536	3.917978	-1.555169
H	0.969631	3.770453	-2.567336
H	0.560930	4.298380	-0.920343
H	2.197067	4.652081	-1.578740
C	4.176639	-0.779441	-3.712117
H	3.895867	-1.813266	-3.968762
H	3.730216	-0.115773	-4.472870
H	5.269496	-0.665250	-3.733916
C	3.966889	-3.326184	1.540419
H	3.144696	-3.869124	2.031655
H	4.323123	-3.945025	0.698067
H	4.801024	-3.176241	2.240694
C	1.117783	1.479329	3.660276
H	1.850756	2.076297	4.220713
H	0.198895	2.064582	3.490945
H	0.838189	0.589584	4.249858

Egas = -3375.85145971
No imaginary frequency
Zero-point correction = 0.498606
Sum of electronic and thermal Enthalpies = -3375.306581
Sum of electronic and thermal Free Energies = -3375.436824
Gsolv= -3376.002852

S5-7. Rh-Nitrenes C-H insertion mechanisms for **B** (Figure 3)

$^{1}TS_{B}$

Rh -0.290962 0.371405 -0.209157
Rh -1.918899 -1.294608 0.413697
O 0.457005 -0.979865 -1.589531
O -1.117692 -2.544063 -1.000550
O -1.079313 1.598998 1.232995
O -2.655356 0.028385 1.794918
O 0.968224 -0.419828 1.201583
O -0.576625 -2.000324 1.785939
O -1.687966 1.014169 -1.581450
O -3.224364 -0.567878 -0.960649
C -0.118638 -2.126812 -1.689341
C 0.566895 -1.435916 1.888542
C -2.090678 1.176087 1.906144
C -2.830338 0.436642 -1.660769
N 1.009159 1.760006 -0.871606
C 0.807319 3.100688 -0.603308
O -0.268632 3.653276 -0.806757
O 1.869284 3.862692 -0.127020
C 3.174393 3.305678 -0.279760
C 3.239757 1.878186 0.194239
H 3.849209 3.957164 0.310309
H 3.490576 3.371796 -1.341816
H 2.819831 1.702083 1.195360
H 2.159929 1.409779 -0.672155

C -3.800323 1.010307 -2.667473	H -3.320538 1.615124 3.623801
H -4.096094 2.024074 -2.348817	C 4.431801 1.041515 -0.182921
H -3.308151 1.106091 -3.648453	H 5.302830 1.404427 0.412400
H -4.692302 0.373401 -2.748755	H 4.700483 1.224509 -1.242101
C 0.438942 -3.063254 -2.739726	C 4.261077 -0.467622 0.065976
H 0.233279 -2.650794 -3.742197	H 3.999955 -0.630986 1.129125
H 1.533326 -3.139199 -2.634229	H 3.396080 -0.831474 -0.520212
H -0.020171 -4.057917 -2.653298	C 5.511657 -1.282252 -0.290967
C 1.545602 -2.003567 2.890979	H 5.777065 -1.099699 -1.350514
H 2.419173 -2.418771 2.359611	H 6.371168 -0.920043 0.306691
H 1.910490 -1.204209 3.556381	C 5.321694 -2.786175 -0.062452
H 1.067992 -2.796074 3.483674	H 4.491678 -3.179576 -0.676763
C -2.680756 2.145889 2.904471	H 6.230593 -3.354514 -0.323536
H -1.878730 2.690159 3.426207	H 5.084655 -3.002861 0.995178
H -3.289659 2.890748 2.363396	

Egas = -1613.40076640
One imaginary frequency of -883.7165
Zero-point correction= 0.394806
Sum of electronic and thermal Enthalpies= -1612.970972
Sum of electronic and thermal Free Energies= -1613.074205
Gsolv= -1613.494127

Oxazolidinone from B • $Rh_2(O_2CCH_3)_4$

Rh	0.189055	0.325986	-0.213855	H	-3.732049	2.882816	0.930180
Rh	1.914594	-1.224778	0.431715	H	-2.437704	1.727377	1.453456
O	-0.940598	-0.397827	1.365543	H	-3.381075	2.331520	-1.420470
O	0.693832	-1.902415	1.932186	H	-1.117893	1.760883	-2.039875
O	1.412645	0.955078	-1.737107	C	-3.338091	0.281060	-0.718173
O	3.070387	-0.490668	-1.089142	H	-3.186104	-0.150860	-1.725316
O	-0.549641	-1.126572	-1.486280	H	-2.738475	-0.324528	-0.021604
O	1.098524	-2.593873	-0.858866	C	-4.830660	0.215696	-0.351324
O	1.026465	1.682060	1.078916	H	-5.405886	0.896118	-1.010120
O	2.670749	0.203196	1.686292	H	-4.980583	0.586351	0.683008
C	-0.446085	-1.341723	2.091569	C	-5.422842	-1.201800	-0.458135
C	0.057469	-2.261926	-1.528709	H	-5.302782	-1.565112	-1.497471
C	2.577559	0.418502	-1.848757	H	-6.512505	-1.142312	-0.278200
C	2.083395	1.344171	1.729761	C	-4.807637	-2.218268	0.513510
N	-1.350704	1.849829	-1.043876	H	-3.732699	-2.376860	0.317465
C	-0.964315	3.150651	-0.542568	H	-4.910802	-1.878492	1.560767
O	-0.071854	3.853173	-0.955890	H	-5.307547	-3.198574	0.431533
O	-1.793755	3.468071	0.493090	C	3.429159	0.896508	-3.003794
C	-2.776926	2.412269	0.656678	H	3.302280	1.980914	-3.144236
C	-2.798081	1.708569	-0.708266	H	4.486333	0.651428	-2.827862

H	3.100804	0.395077	-3.931184	H	-2.334752	-1.970720	2.926834
C	-0.539433	-3.312738	-2.440880	H	-0.884729	-2.741359	3.676677
H	0.189585	-4.112467	-2.634150	C	2.700360	2.410539	2.606518
H	-1.428492	-3.754041	-1.956882	H	3.392618	1.960689	3.332381
H	-0.866574	-2.855869	-3.387905	H	3.259109	3.119196	1.970660
C	-1.294144	-1.813303	3.253453	H	1.913054	2.980478	3.123529
H	-1.304772	-1.035800	4.037009				

Egas = -1613.50308634
No imaginary frequency
Zero-point correction= 0.404801
Sum of electronic and thermal Enthalpies = -1613.063638
Sum of electronic and thermal Free Energies = -1613.166436
Gsolv= -1613.580989

S5-8. Ketone Formation from **D** (Figure 10).

^{1}NR$_D$

Rh -1.809406 -0.069039 -0.494167
Rh -3.781514 0.161903 0.894100
O -0.686405 -0.242608 1.224010
O -2.558102 -0.022303 2.535876
O -3.037899 0.129491 -2.143952
O -4.900121 0.331411 -0.813779
O -1.655717 1.985467 -0.356992
O -3.532442 2.176564 0.933339
O -4.042517 -1.850411 0.860380
C -1.298873 -0.175722 2.356346
C -2.533385 2.663236 0.293473
C -4.301443 0.271318 -1.948236
C -3.184760 -2.551550 0.214514
N -0.302986 -0.234240 -1.651177
C 0.914220 -0.632678 -1.176223
O 1.166324 -1.850105 -1.148861
O 1.772055 0.363924 -0.866247
C -0.424220 -0.270496 3.586483
H 0.269994 -1.120266 3.490542
H 0.183393 0.646695 3.672600
H -1.042254 -0.381744 4.488284
C -2.372563 4.164421 0.271387
H -1.309725 4.435357 0.363302
H -2.734128 4.551240 -0.697232
H -2.960546 4.622204 1.079570
C -3.344316 -4.052266 0.218346
H -3.310313 -4.435253 -0.814007
H -2.501544 -4.507351 0.765520
H -4.291597 -4.333421 0.699332
C -5.164760 0.342927 -3.187828
H -4.614719 0.824075 -4.009940
H -5.422118 -0.682896 -3.504921
H -6.097313 0.884627 -2.972907
C 3.125449 -0.051465 -0.491335
C 3.974604 -0.300467 -1.740302
C 3.701330 1.067379 0.374081
H 3.041453 -0.986757 0.092101
C 5.428230 -0.619209 -1.349729
H 3.941814 0.607034 -2.373643
H 3.537651 -1.128859 -2.323562
C 5.155031 0.752979 0.766646
H 3.658941 2.013378 -0.199406
H 3.072773 1.205212 1.272049
C 6.046797 0.495099 -0.471982
H 6.040800 -0.764822 -2.257208
H 5.459929 -1.578404 -0.796593
H 5.573826 1.583542 1.362513
H 5.175772 -0.140435 1.421345
H 6.046241 1.426175 -1.075436
C 7.488956 0.198806 -0.091081
C 8.528038 1.058090 -0.495527
C 7.827835 -0.936835 0.673217
C 9.862409 0.796754 -0.151703
H 8.284438 1.946211 -1.091038

C 9.159108 -1.202870 1.020010
H 7.041485 -1.626281 1.001987
C 10.183050 -0.336338 0.608660
H 10.652346 1.480719 -0.480119
H 9.398041 -2.091665 1.613929
H 11.223571 -0.544241 0.878903

Egas = -1842.98746241
No imaginary frequency
Zero-point correction= 0.459653
Sum of electronic and thermal Enthalpies= -1842.489475
Sum of electronic and thermal Free Energies= -1842.602397
Gsolv= -1843.084346

β-C-H amination TS for substrate D

Rh 1.288988 0.283102 -0.233848
Rh 3.011555 -1.272345 0.425616
O 0.033805 -0.633027 1.101554
O 1.679213 -2.077024 1.756932
O 2.682801 1.069513 -1.531608
O 4.294259 -0.436472 -0.908065
O 0.700125 -1.078866 -1.673214
O 2.333320 -2.556497 -1.024747
O 1.938249 1.535406 1.261822
O 3.621412 0.082327 1.835709
C 0.491372 -1.607745 1.812995
C 1.349725 -2.186334 -1.760887
C 3.860913 0.564536 -1.589575
C 2.963276 1.179259 1.952253
N -0.165379 1.512964 -0.897575
C -0.237618 2.860482 -0.599606
O 0.721743 3.616436 -0.703694
O -1.479042 3.395858 -0.234941
H -1.190083 0.975975 -0.719145
C 3.441651 2.170555 2.989715
H 3.874291 3.047607 2.478882
H 2.589762 2.528920 3.589176
H 4.201226 1.713405 3.639219
C -0.464692 -2.223318 2.809325
H -0.640113 -1.514341 3.636819
H -1.436363 -2.424157 2.330032
H -0.044860 -3.153858 3.216239
C 0.927230 -3.127682 -2.867995
H -0.159911 -3.070240 -3.028225
H 1.423754 -2.823461 -3.806129
H 1.230190 -4.157986 -2.631827
C 4.826686 1.230230 -2.542815
H 4.354086 1.354292 -3.530071
H 5.069609 2.238415 -2.166294
H 5.749346 0.639703 -2.631563
C -2.602626 2.545005 -0.474450
C -2.337140 1.172698 0.077057
C -3.881097 3.101758 0.173843
H -2.766933 2.473038 -1.572652
C -3.397329 0.155369 -0.206561
H -1.923438 1.170952 1.095547
C -5.044216 2.122072 -0.077218
H -3.703323 3.231260 1.257466
H -4.106567 4.098062 -0.245424
C -4.743189 0.687078 0.423039
H -3.150980 -0.829002 0.223625
H -3.547067 0.023045 -1.294502
H -5.959785 2.490934 0.417868
H -5.269069 2.091887 -1.160858
H -4.581662 0.742303 1.517495
C -5.872992 -0.295297 0.169033
C -6.355506 -0.540499 -1.133190
C -6.451634 -1.004575 1.239201
C -7.388074 -1.460740 -1.355420
H -5.920713 -0.007755 -1.986712
C -7.485292 -1.926418 1.021045
H -6.086807 -0.827959 2.258107
C -7.956968 -2.157903 -0.278634
H -7.749149 -1.635286 -2.374383
H -7.921825 -2.464382 1.869070
H -8.762382 -2.878562 -0.453233

Egas = -1842.96600292
One imaginary frequency -451.9353
Zero-point correction= 0.454601
Sum of electronic and thermal Enthalpies= -1842.473689
Sum of electronic and thermal Free Energies=-1842.583921
Gsolv= -1843.074813

α-C-H amination TS for substrate D

Rh	1.390167	0.284861	-0.213572
Rh	3.372743	-0.929972	0.449142
O	1.019086	0.621698	1.788100
O	2.915615	-0.516318	2.403609
O	1.848293	-0.138063	-2.174316
O	3.737229	-1.277465	-1.540197
O	0.349083	-1.505090	-0.099898
O	2.237820	-2.636698	0.540649
O	2.577621	1.966620	-0.303509
O	4.445694	0.803095	0.343012
C	1.861559	0.170435	2.650785
C	0.992540	-2.563597	0.256106
C	2.917766	-0.818118	-2.410979
C	3.826244	1.868240	-0.015791
N	-0.270665	1.305597	-0.761363
C	-0.839071	2.550156	-0.375646
O	-0.315591	3.514739	0.119409
O	-2.233889	2.408324	-0.583378
H	-0.784132	1.126304	-1.992279
C	1.584644	0.513316	4.098866
H	1.913326	1.549186	4.293134
H	0.504480	0.462177	4.305387
H	2.136841	-0.163671	4.766279
C	0.180303	-3.837434	0.366785
H	-0.449760	-3.793646	1.272306
H	-0.489083	-3.940123	-0.502044
H	0.844693	-4.710125	0.438450
C	4.640869	3.137421	-0.138461
H	4.845009	3.337395	-1.204769
H	4.070827	3.991890	0.257600
H	5.597699	3.031516	0.392489
C	3.239289	-1.071998	-3.868419
H	2.329855	-1.371958	-4.412556
H	3.609224	-0.139203	-4.328318
H	4.012789	-1.847355	-3.961278
C	-2.322141	1.006647	-0.661869
C	-3.037379	0.485152	-1.874219
C	-2.573415	0.275848	0.624421
C	-4.565445	0.475170	-1.579558
H	-2.699774	-0.547186	-2.081062
H	-2.824576	1.109737	-2.760573
C	-4.099248	0.248023	0.903646
H	-2.181280	-0.751843	0.524581
H	-2.024014	0.757752	1.449543
C	-4.891530	-0.322832	-0.295601
H	-5.096788	0.044101	-2.446345
H	-4.917271	1.517826	-1.472280
H	-4.295407	-0.353054	1.809116
H	-4.445267	1.275843	1.120550
H	-4.544601	-1.364435	-0.457206
C	-6.388480	-0.375107	-0.026188
C	-7.070750	-1.605972	-0.029387
C	-7.127867	0.797123	0.232386
C	-8.449433	-1.670103	0.218981
H	-6.511141	-2.527771	-0.228562
C	-8.505644	0.738020	0.480939
H	-6.623024	1.770064	0.242306
C	-9.171979	-0.496664	0.475378
H	-8.958518	-2.639650	0.212575
H	-9.060764	1.660645	0.680558
H	-10.248304	-0.542669	0.670366

Zero-point correction= 0.452606
One imaginary frequency -179.5101
Sum of electronic and thermal Enthalpies= -1842.469446
Sum of electronic and thermal Free Energies= -1842.581161
Gsolv= -1843.068293

Salt-K_D

C -1.704119 -0.397793 -0.343144
O -1.568763 0.858614 -0.374949
N -2.773056 -1.187901 -0.582554
O -3.900549 -0.278825 -0.962878
S -5.059606 -0.253277 0.213471
C -5.464712 -1.989756 0.416228
H -4.496137 -2.509888 0.485504
H -6.061586 -2.095584 1.332880
H -6.024837 -2.313511 -0.471967
O -4.485859 0.265123 1.487809
O -6.165956 0.519273 -0.403457
O -0.635515 -1.224556 -0.042077
K -3.472955 2.414775 -0.037460
C 0.642958 -0.576421 0.114945
C 1.502479 -1.497489 0.983255
C 1.302856 -0.322210 -1.246505
H 0.487825 0.393244 0.625513
C 2.920720 -0.929473 1.155490
H 1.550394 -2.492986 0.500894
H 1.017023 -1.641855 1.964982
C 2.721035 0.247679 -1.080283
H 1.342447 -1.281703 -1.798039
H 0.670326 0.368248 -1.830518
C 3.608675 -0.670931 -0.206992
H 3.537518 -1.618258 1.760529
H 2.871408 0.021810 1.722036
H 3.194231 0.395881 -2.067875
H 2.664797 1.249751 -0.609913
H 3.678065 -1.648225 -0.727744
C 5.023313 -0.137450 -0.049528
C 6.124799 -0.874819 -0.523530
C 5.276733 1.102724 0.571834
C 7.435301 -0.395777 -0.385045
H 5.948855 -1.841945 -1.009782
C 6.583766 1.586890 0.713235
H 4.439310 1.700037 0.950901
C 7.670084 0.838951 0.234900
H 8.274275 -0.990127 -0.762915
H 6.755221 2.553421 1.199616
H 8.691889 1.216936 0.345242

Egas = -1971.71720012
No imaginary frequency
Zero-point correction= 0.304661
Sum of electronic and thermal Enthalpies= -1971.389152
Sum of electronic and thermal Free Energies= -1971.467947
Gsolv= -1971.832431

Hydride transfer TS for substrate D

Rh	2.158546	-0.494582	-0.714211
Rh	4.124265	-1.485264	-1.746229
O	1.021614	-1.692460	-1.935580
O	2.882928	-2.617388	-2.913103
O	3.418001	0.677840	0.444149
O	5.266109	-0.291036	-0.517852
O	2.305262	0.978779	-2.183627
O	4.102065	-0.032659	-3.181004
O	2.230350	-2.019559	0.666514
O	4.088685	-2.936342	-0.313124
C	1.615542	-2.472584	-2.767767
C	3.214452	0.882640	-3.093643
C	4.689908	0.510153	0.296849
C	3.161329	-2.906913	0.568020
N	0.420169	0.382102	0.139900
C	-0.687545	0.569111	-0.628926
O	-0.659996	1.241380	-1.680852
O	-1.856054	0.023463	-0.131329
C	0.718941	-3.282537	-3.679507
H	-0.104896	-3.729854	-3.101179
H	0.269422	-2.613480	-4.433581
H	1.296039	-4.065708	-4.191095
C	3.228620	1.972249	-4.145544
H	2.206646	2.150677	-4.516787
H	3.596497	2.915546	-3.703500
H	3.891435	1.693398	-4.976746
C	3.158227	-4.007362	1.607667
H	3.400479	-3.585075	2.598306
H	2.156429	-4.461314	1.677200
H	3.902260	-4.774582	1.351824

C	5.587783	1.323269	1.206982
H	5.078562	2.239121	1.542477
H	5.835751	0.721991	2.099569
H	6.529594	1.569672	0.694251
C	-3.074592	0.294692	-0.880884
C	-3.681499	-1.041216	-1.315815
C	-4.043784	1.096237	-0.007946
H	-2.787704	0.886809	-1.767574
C	-5.015107	-0.820668	-2.050177
H	-3.845262	-1.670590	-0.418505
H	-2.963511	-1.578258	-1.961718
C	-5.376705	1.323500	-0.743239
H	-4.229247	0.537992	0.931332
H	-3.585720	2.063284	0.270231
C	-6.018374	-0.010686	-1.193534
H	-5.461716	-1.791401	-2.330906
H	-4.827469	-0.278006	-2.997377
H	-6.079307	1.877789	-0.095735
H	-5.202383	1.961809	-1.631473
H	-6.219696	-0.603189	-0.277361
C	-7.345730	0.194106	-1.906791
C	-8.530922	-0.361703	-1.389059
C	-7.428526	0.935821	-3.103300
C	-9.760715	-0.186071	-2.040056
H	-8.485904	-0.941875	-0.459418
C	-8.654453	1.114970	-3.757785
H	-6.522724	1.380913	-3.531035
C	-9.826698	0.554155	-3.228517
H	-10.668600	-0.628652	-1.616275
H	-8.694656	1.695778	-4.685598
H	-10.784793	0.694374	-3.739777
H	1.817086	-0.769441	2.882943
C	0.824873	-0.825985	3.360888
C	-0.018635	0.342911	2.909902
C	0.168395	-2.196802	3.153252
H	0.983330	-0.641035	4.446843
C	-1.514099	0.270364	3.037813
H	0.117138	-0.016058	1.250898
C	-1.301284	-2.225256	3.627165
H	0.755851	-2.960135	3.693479
H	0.225798	-2.456695	2.081933
C	-2.101628	-1.140379	2.873751
H	-1.983630	0.981219	2.335964
H	-1.721444	0.679526	4.051172
H	-1.318064	-1.980084	4.709404
H	-2.126267	-1.383735	1.798182
H	-3.148883	-1.131464	3.224073
O	0.608068	1.485131	2.908221
C	-1.920286	-3.604155	3.455994
C	-2.384442	-4.325751	4.571509
C	-2.041645	-4.196155	2.181495
C	-2.951986	-5.600153	4.425493
H	-2.299448	-3.880130	5.569986
C	-2.607960	-5.469167	2.031164
H	-1.692229	-3.656730	1.293344
C	-3.065236	-6.177075	3.153074
H	-3.306123	-6.141885	5.308984
H	-2.692867	-5.910036	1.032079
H	-3.507728	-7.171593	3.034964
C	-0.222039	2.840931	3.033295
O	-0.818011	3.002306	4.079107
N	0.053735	3.430683	1.881446
O	-0.664778	4.728118	1.896597
S	0.387981	5.950045	1.488935
O	1.258520	5.516347	0.366191
O	-0.465518	7.136421	1.354478
C	1.413773	6.071597	2.967767
H	0.769849	6.371906	3.806274
H	1.866202	5.086582	3.152818
H	2.189075	6.827624	2.774942
K	1.270298	2.916240	0.551487

Egas = -3814.72909373
One imaginary frequency -199.3511
Zero-point correction= 0.760516
Sum of electronic and thermal Enthalpies= -3813.906409
Sum of electronic and thermal Free Energies= -3814.073293
Gsolv= -3814.923991

INT$_D$

Rh	0.166667	-0.888763	0.489973
Rh	-0.116284	-0.534755	2.879284
O	1.918435	-1.869976	0.923291
O	1.633411	-1.548698	3.180875
O	-1.615930	0.146458	0.195031
O	-1.869983	0.461773	2.451615
O	1.175272	0.940420	0.448366
O	0.950528	1.206454	2.715491
O	-0.873952	-2.653685	0.703229
O	-1.158347	-2.296712	2.949842
C	2.270379	-1.984951	2.155308
C	1.356489	1.571860	1.561641
C	-2.245660	0.586468	1.237902
C	-1.322061	-2.966941	1.872874
N	0.287118	-1.440378	-1.603603
C	1.301086	-1.399271	-2.501007
O	1.628480	-2.278593	-3.323523
O	1.940786	-0.153391	-2.485338
C	3.589553	-2.683049	2.412075
H	3.721826	-3.519789	1.709168
H	4.416358	-1.970334	2.245638
H	3.638539	-3.040169	3.450844
C	2.109070	2.884220	1.471764
H	1.451177	3.662228	1.044243
H	2.430759	3.210246	2.470959
H	2.980576	2.777924	0.806181
C	-2.152901	-4.228421	1.973559
H	-1.914565	-4.931540	1.162006
H	-1.996229	-4.703998	2.953454
H	-3.222408	-3.962668	1.897024
C	-3.564756	1.287754	0.987401
H	-3.698286	2.111355	1.705177
H	-3.628286	1.664860	-0.044713
H	-4.390092	0.570950	1.146329
C	3.041525	0.005252	-3.413203
C	2.547546	0.588643	-4.742645
C	4.079914	0.914407	-2.754875
H	3.468524	-0.996035	-3.599442
C	3.721473	0.854867	-5.700750
H	2.013042	1.540595	-4.539632
H	1.822510	-0.108285	-5.198804
C	5.258459	1.175827	-3.708629
H	3.599339	1.876442	-2.483240
H	4.429029	0.454696	-1.813159
C	4.790553	1.770680	-5.059045
H	3.355058	1.306845	-6.640053
H	4.188896	-0.109080	-5.981088
H	5.989283	1.855519	-3.235016
H	5.794480	0.225303	-3.898217
H	4.300940	2.741996	-4.837572
C	5.952563	2.049093	-5.999800
C	6.239635	3.362487	-6.416997
C	6.773065	1.006884	-6.478515
C	7.309103	3.632246	-7.283427
H	5.612817	4.186399	-6.054574
C	7.842785	1.270337	-7.344314
H	6.574402	-0.026452	-6.171150
C	8.115578	2.585352	-7.750690
H	7.511508	4.663170	-7.593117
H	8.466412	0.444596	-7.703419
H	8.951397	2.791423	-8.427336
H	-3.055278	-1.277366	-2.005984
C	-3.055313	-2.100667	-2.745451
C	-2.063858	-1.775385	-3.817241
C	-2.878561	-3.469809	-2.071203
H	-4.042642	-2.030130	-3.251550
C	-1.192784	-2.812038	-4.453863
H	-0.073849	-2.400734	-1.603141
C	-2.469830	-4.575877	-3.068860
H	-3.826030	-3.741567	-1.573625
H	-2.121978	-3.389919	-1.270858
C	-1.145593	-4.181424	-3.753918
H	-0.160823	-2.407458	-4.534000
H	-1.566829	-2.899685	-5.495284
H	-3.257677	-4.643671	-3.847832
H	-0.328589	-4.158972	-3.011711
H	-0.857332	-4.947804	-4.493688
O	-2.026459	-0.616479	-4.362252
C	-3.233767	0.490169	-3.983255
N	-2.501601	1.413960	-3.403958
O	-3.452055	2.465244	-2.961208
S	-2.703960	3.954060	-3.066764
O	-3.673002	4.873342	-2.460601
O	-1.319019	3.856664	-2.544160
C	-2.613178	4.200444	-4.849966
H	-2.083709	3.340550	-5.285637

H -3.638990 4.272380 -5.238187
H -2.058763 5.133718 -5.027870
O -4.343506 0.166874 -4.329299
C -2.375802 -5.932895 -2.387147
C -3.266587 -6.968139 -2.724986
C -1.404601 -6.184263 -1.395157
C -3.196057 -8.219082 -2.093990
H -4.026593 -6.790434 -3.495397
C -1.330164 -7.433530 -0.763446
H -0.695271 -5.398071 -1.111044
C -2.226416 -8.456063 -1.110053
H -3.900256 -9.009491 -2.374489
H -0.565515 -7.608881 0.000906
H -2.167079 -9.431758 -0.616937
K -0.210995 1.422667 -1.844247

Egas = -3814.76312190
Zero-point correction= 0.763995
No imaginary frequency
Sum of electronic and thermal Enthalpies= -3813.935650
Sum of electronic and thermal Free Energies= -3814.107425
Gsolv= -3814.956472

4-Phenylcyclohexyl carbamate (Primary carbamate from D)

C 0.957943 -0.532800 -0.220130
C 0.145870 0.523097 -1.007215
C -1.335815 0.129033 -1.130004
C -1.951386 -0.114493 0.251372
C -1.174440 -1.169399 1.037888
C 0.306930 -0.775118 1.163360
H -1.906463 0.918809 -1.648043
H 0.224621 1.500990 -0.493201
H 0.586703 0.664401 -2.010187
H 0.890331 -1.485605 -0.784484
H -1.986478 0.839697 0.808058
H -1.629501 -1.302940 2.035498
H -1.260782 -2.139952 0.511549
H 0.394556 0.146605 1.771342
H 0.863117 -1.559436 1.707140
H -1.432345 -0.799467 -1.725999
O -3.317338 -0.600343 0.122568
C -4.272843 0.375281 -0.012269
O -4.062839 1.582964 -0.035953
N -5.519179 -0.210131 -0.064722
H -6.265315 0.386895 -0.409843
H -5.575971 -1.200019 -0.292458
C 2.430756 -0.171392 -0.109583
C 3.416370 -1.001463 -0.675828
C 2.851923 0.995689 0.560023
C 4.778300 -0.682102 -0.579544
H 3.108050 -1.913620 -1.200940
C 4.211155 1.320173 0.659035
H 2.107456 1.661530 1.011420
C 5.180920 0.481779 0.089286
H 5.525544 -1.345173 -1.028672
H 4.514652 2.232428 1.183869
H 6.243386 0.735090 0.166742

Egas = -709.972239418
No imaginary frequency
Zero-point correction= 0.276526
Sum of electronic and thermal Enthalpies= -709.679919
Sum of electronic and thermal Free Energies= -709.739134
Gsolv= -709.946439

4-Phenylcyclohexanone (ketone of D)

C -0.974789 0.145741 1.261121
C -2.475402 -0.215715 1.309901
C -3.179290 0.076348 -0.012952
C -2.465523 -0.468541 -1.247768

C -0.965453 -0.103490 -1.257550
C -0.261218 -0.511108 0.057053
H -2.575351 -1.302741 1.508989
H -3.003076 0.319521 2.116103
H -0.867101 1.245184 1.192442
H -0.483748 -0.156951 2.203046
H -2.565222 -1.573512 -1.230959
H -2.986289 -0.101374 -2.147088
H -0.466356 -0.583662 -2.117956
H -0.857101 0.988303 -1.403544
H -0.370191 -1.610244 0.165764
O -4.233977 0.697958 -0.078261
C 1.227982 -0.201515 0.027256
C 2.175739 -1.238488 0.111574
C 1.699836 1.121549 -0.091315
C 3.551308 -0.968593 0.078914
H 1.827337 -2.274340 0.203445
C 3.073094 1.396792 -0.124384
H 0.986004 1.950491 -0.160301
C 4.005198 0.351959 -0.039543
H 4.268791 -1.793375 0.145771
H 3.416757 2.432537 -0.217431
H 5.078470 0.566862 -0.066038

Egas = -540.240155385
No imaginary frequency
Zero-point correction= 0.226521
Sum of electronic and thermal Enthalpies= -540.001337
Sum of electronic and thermal Free Energies= -540.051779
Gsolv= -540.200558

MsON=C=O

N -1.295141 0.952679 -0.373709
C -2.180983 0.109865 -0.081665
O -3.160372 -0.436660 0.282763
O -0.116875 0.542866 -1.053368
S 0.994013 -0.258475 0.007464
C 1.555148 1.134132 1.006928
H 0.669331 1.606763 1.455552
H 2.219850 0.734117 1.786762
H 2.091340 1.833774 0.351298
O 0.221028 -1.183360 0.854512
O 2.048250 -0.694320 -0.914989

Egas = -831.092688105
No imaginary frequency
Zero-point correction= 0.062117
Sum of electronic and thermal Enthalpies= -831.020895
Sum of electronic and thermal Free Energies= -831.065221
Gsolv= -831.228173

HN=C=O

N 0.616168 -1.009764 0.000000
C 0.000000 0.054001 0.000000
O -0.742940 0.977421 0.000000
H 1.630348 -1.075028 0.000000

Egas = -168.498974285
No imaginary frequency
Zero-point correction= 0.020809
Sum of electronic and thermal Enthalpies= -168.473940
Sum of electronic and thermal Free Energies= -168.501133
Gsolv= -168.564101

S5-9. By-products formation for Substrate **E** (Figure 12)

$^{1}NR_{E}$

Rh -0.832720 -0.218605 0.102282
Rh -3.095842 0.634445 0.040176
O -0.795812 0.291223 2.093729
O -2.955356 1.072142 2.028748
O -0.976702 -0.651409 -1.909939
O -3.129729 0.135799 -1.962060
O -0.167991 1.693739 -0.377214
O -2.323808 2.478581 -0.426313
O -1.631111 -2.053526 0.553377
O -3.775174 -1.234372 0.520902
C -1.848914 0.799118 2.623356
C -1.056326 2.615689 -0.528381
C -2.096012 -0.390462 -2.499300
C -2.904954 -2.165445 0.677259
N 1.081982 -1.056211 0.129070
C 1.743691 -1.928769 0.956659
O 1.399384 -2.221913 2.091791
O 2.890310 -2.484087 0.337210
C 4.132986 -2.343734 1.034150
C 5.051713 -1.356950 0.275121
H 4.618474 -3.335023 1.054623
H 3.946529 -2.027408 2.076018
H 6.068665 -1.478740 0.690001
H 5.115905 -1.702732 -0.774475
C 4.685840 0.114532 0.353092
C 3.581098 0.719925 -0.301765
C 5.490100 0.956401 1.149330
C 3.296466 2.089664 -0.143204
C 5.226732 2.323464 1.293470
H 6.349998 0.516877 1.666755
C 4.123964 2.895265 0.646779
H 2.394567 2.512008 -0.595547
H 5.879776 2.938412 1.920781
H 3.895914 3.958750 0.766277
C -1.779838 1.078362 4.109623
H -1.886492 0.127172 4.659465
H -0.797897 1.502662 4.370437
H -2.587920 1.759315 4.413028
C -0.537852 4.008206 -0.828461
H -0.191571 4.478041 0.109025
H 0.318394 3.959616 -1.520133
H -1.335731 4.631111 -1.257491
C -3.416566 -3.543305 1.039778
H -3.141542 -4.260945 0.248648
H -2.932915 -3.881768 1.970738
H -4.508363 -3.529832 1.164840
C -2.196772 -0.781747 -3.958771
H -1.215356 -0.702478 -4.451160
H -2.528802 -1.832624 -4.029262
H -2.936607 -0.151819 -4.473853
I 2.215890 -0.406615 -1.402210

Egas = -1697.85818006
No imaginary frequency
Zero-point correction= 0.358347
Sum of electronic and thermal Enthalpies= -1697.463927
Sum of electronic and thermal Free Energies= -1697.569696
Gsolv= -1698.065509

β-C-H amination TS for substrate E

Rh 0.842750 -0.127059 0.451924
Rh 2.644574 0.826888 -0.841143
O 0.652050 -1.546784 -1.038015
O 2.403666 -0.659209 -2.225537
O 1.143993 1.350771 1.857784
O 2.816907 2.282648 0.591652
O -0.412352 1.153785 -0.514902
O 1.261808 1.949820 -1.845412
O 2.313534 -1.267840 1.349700
O 4.005698 -0.298916 0.150790
C 1.459234 -1.506940 -2.040368
C 0.035412 1.869487 -1.489535
C 2.058591 2.225713 1.624947
C 3.545982 -1.109325 1.037877

N -0.431555 -0.932153 1.740435
C -0.824680 -2.245208 1.690960
O -0.031316 -3.168659 1.506371
O -2.154723 -2.555681 1.918400
C -3.085364 -1.574962 2.404211
C -2.748661 -0.087413 2.150344
H -3.164231 -1.720570 3.497287
H -4.059325 -1.845450 1.959693
H -2.704576 0.458191 3.107717
H -1.496604 -0.188272 1.932001
C -3.484301 0.705786 1.145683
C -4.110769 1.899654 1.589647
C -3.615565 0.350479 -0.230803
C -4.889729 2.685057 0.741560
H -3.992594 2.186437 2.640389
C -4.411170 1.148159 -1.077157
C -5.053194 2.294964 -0.597946
H -5.374783 3.589682 1.120852
H -4.523033 0.882452 -2.133484
H -5.667526 2.894078 -1.277547
C 4.544850 -1.943981 1.804424
H 5.507405 -1.971115 1.273895
H 4.698066 -1.499785 2.803211
H 4.150692 -2.961618 1.947592
C 1.239330 -2.554051 -3.109078
H 1.013551 -3.525929 -2.643309
H 0.369597 -2.264765 -3.726616
H 2.121992 -2.633302 -3.759288
C -0.994594 2.635347 -2.283054
H -1.902188 2.791374 -1.681096
H -0.580339 3.593028 -2.631826
H -1.272381 2.038075 -3.171125
C 2.274814 3.271982 2.695707
H 1.317774 3.541808 3.166878
H 2.932318 2.854937 3.478475
H 2.760583 4.161048 2.268342
I -2.484498 -1.163312 -1.003611

Egas = -1697.81803545
One imaginary frequency -596.6745
Zero-point correction= 0.353047
Sum of electronic and thermal Enthalpies= -1697.429629
Sum of electronic and thermal Free Energies= -1697.532999
Gsolv= -1698.046863

Oxazolidinone from E • $Rh_2(O_2CCH_3)_4$

Rh 0.742597 -0.377534 -0.114294
Rh 2.834029 0.666635 0.477269
O -0.087026 0.597307 1.507310
O 1.900543 1.599565 2.034807
O 1.737281 -1.263243 -1.700312
O 3.715328 -0.266353 -1.112521
O 1.265927 -1.958135 1.103886
O 3.251777 -0.951566 1.661226
O 0.312787 1.282513 -1.262360
O 2.319336 2.248980 -0.714829
C 0.646415 1.419177 2.192437
C 2.392396 -1.901164 1.724480
C 2.994651 -1.020637 -1.853491
C 1.173901 2.243921 -1.291208
N -2.527289 1.705882 0.429070
C -2.461060 3.039715 0.088708
O -1.683684 3.892496 0.493402
O -3.471942 3.316427 -0.819607
C -4.270757 2.125685 -0.982341
C -3.325345 0.982547 -0.561157
H -4.591138 2.056278 -2.032617
H -5.159296 2.213802 -0.331100
H -2.691524 0.732266 -1.437154
H -1.674137 1.276541 0.818264
C -3.910621 -0.301473 -0.000984
C -3.166521 -1.506368 -0.039072
C -5.163368 -0.326571 0.640338
C -3.671246 -2.682519 0.543670
C -5.677638 -1.499239 1.205788
H -5.750452 0.594075 0.706619
C -4.928316 -2.681088 1.160059
H -3.076273 -3.600778 0.533632
H -6.658315 -1.483795 1.691313
H -5.314065 -3.602443 1.607421

C 0.767270 3.487658 -2.043738
H 0.260653 3.219761 -2.984197
H 0.050196 4.049063 -1.418035
H 1.644515 4.118626 -2.245340
C -0.070295 2.254860 3.222233
H -0.623170 3.048610 2.685457
H -0.800965 1.644747 3.776251
H 0.649756 2.716416 3.912457
C 2.725230 -3.081392 2.611021
H 1.922713 -3.226254 3.353003
H 2.779127 -3.998461 2.000636
H 3.684046 -2.920546 3.123259
C 3.666064 -1.690938 -3.031276
H 3.488038 -2.778150 -2.996103
H 3.226910 -1.313648 -3.970286
H 4.745776 -1.486575 -3.023748
I -1.316542 -1.648438 -1.021659

Egas = -1697.96127720
No imaginary frequency
Zero-point correction= 0.362096
Sum of electronic and thermal Enthalpies= -1697.564166
Sum of electronic and thermal Free Energies= -1697.667196
Gsolv= -1698.155409

Salt-K$_E$

C 0.654960 0.123407 1.936227
O 0.411603 1.358793 1.947923
N 1.744549 -0.576663 1.573086
O 2.779125 0.430609 1.194698
S 3.161042 0.285221 -0.397647
C 3.646293 -1.443838 -0.530070
H 2.918852 -2.016444 0.066567
H 3.622766 -1.725780 -1.592505
H 4.657421 -1.541866 -0.110454
O 1.940014 0.476815 -1.242116
O 4.310639 1.182688 -0.612141
O -0.306147 -0.799299 2.374341
C -1.564552 -0.243557 2.752834
H -1.432297 0.818664 3.020073
C -2.661564 -0.384714 1.669929
K 0.369888 2.412934 -0.314871
C -2.481675 0.418342 0.399516
C -1.819324 -0.093351 -0.751393
C -3.029697 1.717001 0.339156
C -1.778415 0.696861 -1.924939
C -2.964063 2.504602 -0.818563
H -3.535305 2.107478 1.229718
C -2.347351 1.977370 -1.966776
H -1.301908 0.302744 -2.828501
H -3.422298 3.498900 -0.836125
H -2.321431 2.551447 -2.899490
H -2.803574 -1.454113 1.438422
H -3.598676 -0.052157 2.155774
H -1.889770 -0.808883 3.643471
I -0.900372 -1.903438 -0.714921

Egas = -1826.53001741
No imaginary frequency
Zero-point correction= 0.203524
Sum of electronic and thermal Enthalpies= -1826.305480
Sum of electronic and thermal Free Energies= -1826.378031
Gsolv= -1826.799971

TS'$_E$

Rh	2.168049	-0.639418	0.118606
Rh	4.163533	-0.808856	-1.249523
O	2.672099	-2.456439	0.918599
O	4.591765	-2.585885	-0.335870
O	1.772670	1.182931	-0.806889
O	3.633375	0.970852	-2.129261
O	1.159520	-1.645873	-1.371581
O	3.053750	-1.789723	-2.660797

O	3.305092	0.437728	1.486446
O	5.202372	0.156317	0.230569
C	3.762150	-3.022154	0.543258
C	1.814221	-2.002051	-2.423755
C	2.572614	1.573933	-1.750215
C	4.570067	0.575744	1.257844
N	0.499323	-0.480865	1.417010
C	0.438801	-0.859700	2.742469
O	1.373141	-1.293251	3.404982
O	-0.820209	-0.578933	3.318013
C	-1.497476	-1.647744	3.992184
C	-2.716042	-2.112920	3.160817
H	-1.855372	-1.248194	4.956495
H	-0.789142	-2.470671	4.196340
H	-3.360444	-2.704242	3.836717
H	-3.305896	-1.213009	2.893742
C	-2.422359	-2.957088	1.933395
C	-1.853148	-2.458196	0.734518
C	-2.775271	-4.322667	1.951599
C	-1.618260	-3.296139	-0.369742
C	-2.575648	-5.156476	0.843568
H	-3.228566	-4.733910	2.860553
C	-1.991078	-4.644492	-0.322711
H	-1.143665	-2.888321	-1.265666
H	-2.875589	-6.207998	0.895184
H	-1.825657	-5.288634	-1.192057
C	4.111846	-4.321567	1.235514
H	4.548166	-4.095774	2.224138
H	3.202966	-4.919882	1.402084
H	4.845697	-4.888041	0.644270
C	1.037616	-2.775409	-3.469558
H	1.016311	-3.844290	-3.192163
H	-0.001238	-2.413565	-3.520705
H	1.522818	-2.683445	-4.452148
C	5.362633	1.327468	2.306897
H	5.156196	2.410162	2.227297
H	5.064017	0.998667	3.314882
H	6.439908	1.168600	2.157169
C	2.198439	2.860129	-2.450731
H	2.886479	3.055269	-3.285108
H	1.161473	2.789315	-2.819479
H	2.223065	3.709996	-1.746263
I	-1.374044	-0.420417	0.563349
H	-5.129621	1.766875	1.514548
C	-4.308466	1.938149	0.800627
H	-3.395821	0.607891	1.031018
C	-4.762759	1.861260	-0.670526
H	-4.939522	2.904502	-0.992797
H	-5.745448	1.358503	-0.696010
O	-3.622748	3.055074	1.203010
C	-2.149948	3.188619	0.606093
O	-1.357986	2.257394	0.824011
N	-2.126098	4.375825	0.027480
O	-0.738746	4.536692	-0.533951
S	0.199582	5.512187	0.411344
O	0.327492	4.942010	1.780343
O	1.424538	5.723106	-0.386824
C	-0.776900	7.018851	0.519791
H	-0.781481	7.492265	-0.472087
H	-1.793169	6.721519	0.818533
H	-0.306070	7.669220	1.270820
K	1.180764	2.319318	1.628144
C	-3.817373	1.192592	-1.647132
C	-3.810884	-0.210789	-1.855105
C	-2.920208	1.985715	-2.391475
C	-2.927976	-0.769561	-2.802100
C	-2.034676	1.423452	-3.316902
H	-2.914992	3.066326	-2.214801
C	-2.051149	0.039146	-3.533340
H	-2.929453	-1.849769	-2.982574
H	-1.347185	2.066515	-3.875611
H	-1.382005	-0.415110	-4.271250
I	-4.987087	-1.469014	-0.738435

Egas = -3524.38159820
Zero-point correction= 0.554535
One imaginary frequency -844.1042
Sum of electronic and thermal Enthalpies= -3523.769186
Sum of electronic and thermal Free Energies= -3523.924031
Gsolv= -3524.855911

INT'$_E$

Rh	-1.467980	-0.061652	0.475578	H	6.880722	-0.324150	-2.387710
Rh	-3.288982	-0.256437	-1.106224	C	-0.454327	-3.324354	-2.150327
O	-0.682962	-1.757408	-0.386466	H	0.225090	-3.809977	-1.434311
O	-2.298727	-1.800047	-2.016201	H	-1.143746	-4.054881	-2.599787
O	-2.418189	1.593064	1.277430	H	0.155994	-2.883529	-2.960279
O	-4.207613	1.298757	-0.130849	C	-4.333340	-2.778038	2.207714
O	-2.573667	-1.257850	1.723156	H	-3.725646	-3.697628	2.259635
O	-4.199958	-1.577262	0.140796	H	-4.389180	-2.363403	3.226588
O	-0.543472	1.176918	-0.905671	H	-5.338872	-3.023859	1.838147
O	-2.317294	1.064908	-2.338921	C	-0.394193	2.340675	-2.985316
C	-1.217749	-2.215391	-1.462243	H	0.330155	2.978388	-2.455674
C	-3.654534	-1.791709	1.285350	H	0.168716	1.689066	-3.679165
C	-3.594747	1.874872	0.836298	H	-1.092501	2.947534	-3.580657
C	-1.154263	1.471531	-2.010724	C	-4.331903	2.984873	1.555273
N	-0.034798	0.100383	1.936305	H	-4.696250	2.605576	2.525875
C	0.843868	-0.904764	2.224991	H	-3.652156	3.826555	1.761802
O	0.568844	-2.103798	2.268761	H	-5.190510	3.326049	0.959884
O	2.129394	-0.417048	2.490436	H	0.336486	1.049859	2.152409
C	3.112878	-1.452235	2.681649	C	1.456188	2.672157	2.082608
C	3.468905	-2.237575	1.398891	H	1.478014	2.393274	3.141691
H	2.739010	-2.169166	3.432807	C	0.334179	3.447459	1.501215
H	3.995547	-0.925986	3.081623	H	-0.530711	3.383861	2.178694
H	3.984954	-3.150337	1.752500	H	0.039726	3.060301	0.512489
H	2.524987	-2.589712	0.949060	O	2.661279	2.479463	1.547413
C	4.374308	-1.584513	0.370432	C	3.027653	2.881172	0.217439
C	5.755646	-1.868595	0.440027	O	2.292934	3.514033	-0.510192
C	3.916180	-0.783366	-0.708029	I	1.970418	-0.070175	-0.809759
C	6.666028	-1.410149	-0.518000	C	4.484236	2.566555	0.028548
H	6.114802	-2.489328	1.269484	H	5.080292	3.370232	0.500897
C	4.833182	-0.352235	-1.691398	H	4.766367	1.611334	0.495970
C	6.193756	-0.666530	-1.606931	H	4.715269	2.547877	-1.045521
H	7.728967	-1.656385	-0.428649	H	0.611509	4.511883	1.356679
H	4.481475	0.238266	-2.544628				

Egas = -2005.17023488
No imaginary frequency
Zero-point correction= 0.473983
Sum of electronic and thermal Enthalpies= -2004.651998
Sum of electronic and thermal Free Energies= -2004.773569
Gsolv= -2005.379833

Proton transfer TS$_E$

Rh 2.650311 -0.810942 -0.053319
Rh 4.960319 -0.327811 -0.507406
O 2.833230 0.199444 1.726971
O 5.036829 0.656547 1.296465

O 2.556456 -1.764357 -1.872660
O 4.772445 -1.327404 -2.283170
O 3.268851 -2.577547 0.793213
O 5.469990 -2.078968 0.409100
O 2.190513 1.021681 -0.941872
O 4.395277 1.418632 -1.412514
C 3.980394 0.713663 2.020132
C 4.530676 -2.831236 0.848699
C 3.624029 -1.816940 -2.587819
C 3.158916 1.739060 -1.413784
N -3.621411 0.150207 0.864187
C -3.292178 0.709242 -0.332862
O -2.188548 0.586416 -0.891117
O -4.297758 1.503723 -0.842838
C -4.001770 2.148234 -2.094444
C -3.956828 3.676579 -1.960270
H -4.793516 1.855853 -2.805390
H -3.025545 1.802962 -2.454025
H -3.846876 4.082100 -2.987854
H -4.937253 4.034817 -1.601554
C -2.877559 4.254128 -1.059654
C -1.485855 4.130526 -1.300034
C -3.285882 5.020212 0.052012
C -0.566834 4.778626 -0.448191
C -2.373399 5.654290 0.901706
H -4.360595 5.121781 0.239758
C -1.002185 5.534952 0.645932
H 0.508824 4.698233 -0.641679
H -2.730725 6.239646 1.754594
H -0.267170 6.015261 1.298411
C 4.080647 1.421900 3.351882
H 4.882142 2.174544 3.326947
H 3.117984 1.886817 3.614784
H 4.324506 0.683860 4.136520
C 4.927034 -4.141394 1.491156
H 4.546112 -4.181540 2.525405
H 4.468674 -4.978785 0.938967
H 6.020145 -4.253159 1.493937
C 2.793214 3.086538 -1.997188
H 3.394679 3.282392 -2.899524
H 1.720624 3.142415 -2.234985
H 3.045283 3.876171 -1.265369
C 3.505424 -2.496897 -3.934550
H 2.787788 -3.329423 -3.880951
H 3.129302 -1.770058 -4.676062
H 4.488947 -2.856177 -4.270540
H -4.633219 0.015628 0.983288
C -5.832128 -2.860020 0.466227
H -6.047342 -3.211712 1.510018
C -5.634579 -3.979812 -0.564597
H -6.627364 -4.121605 -1.037744
H -5.416184 -4.925805 -0.031154
C -4.602330 -3.685472 -1.631368
C -3.283118 -3.335685 -1.260925
C -4.916966 -3.796874 -2.996091
C -2.309313 -3.109023 -2.247663
C -3.946228 -3.571141 -3.980600
H -5.938597 -4.065104 -3.286608
C -2.640897 -3.227499 -3.604416
H -1.291740 -2.818670 -1.965682
H -4.211331 -3.660262 -5.038981
H -1.874561 -3.045100 -4.364688
O -5.917833 -1.674265 0.167591
C -1.802945 -1.167873 3.268655
O -2.476923 -2.206933 3.324080
N -1.543597 -0.269780 4.199509
O -0.593650 0.719317 3.640394
S -1.101027 2.289773 3.885756
O -0.454785 2.991170 2.740511
O -0.823446 2.749196 5.254710
C -2.882558 2.206253 3.622678
H -3.096349 1.677356 2.677452
H -3.327444 1.668814 4.470761
H -3.223706 3.252227 3.584349
K -0.136778 1.554146 0.393643
H -2.977960 -1.029151 0.976806
O -1.200487 -0.889043 1.882758
C 0.009152 -1.400689 1.622236
O 0.485298 -1.115382 0.500210
C 0.698047 -2.239574 2.660091
H -0.003627 -2.974199 3.089140
H 1.572850 -2.733347 2.218514
H 1.026133 -1.579486 3.482472
I -0.740289 2.954341 -2.822910
I -2.856664 -3.109885 0.749321

Egas = -3753.27539349
One imaginary frequency -719.3493
Zero-point correction= 0.619912
Sum of electronic and thermal Enthalpies= -3752.591988
Sum of electronic and thermal Free Energies= -3752.762505
Gsolv= -3753.744862

Product complex from the protonation of substrate E

Rh 3.234955 -0.127551 -0.535708
Rh 5.472316 0.234268 0.258830
O 2.657711 -0.325971 1.444688
O 4.811443 -0.064463 2.173909
O 3.932427 0.145886 -2.445096
O 6.065083 0.501169 -1.680756
O 3.676135 -2.121462 -0.679782
O 5.810293 -1.770790 0.080134
O 2.895951 1.903833 -0.307589
O 5.047734 2.240843 0.403586
C 3.562730 -0.275677 2.366219
C 4.851654 -2.516544 -0.337665
C 5.181234 0.405397 -2.607677
C 3.871752 2.644318 0.105736
N -0.667884 1.139718 -3.032547
C -1.097910 2.251828 -2.375132
O -0.468985 2.860042 -1.502837
O -2.335752 2.603678 -2.846193
C -2.902755 3.834559 -2.351782
C -3.915664 3.640230 -1.203883
H -3.420900 4.256375 -3.228502
H -2.089898 4.513341 -2.044957
H -4.635583 4.475442 -1.293962
H -4.501763 2.725051 -1.397163
C -3.340951 3.696733 0.197470
C -3.320823 4.957520 0.831882
C -2.831406 2.592353 0.918020
C -2.812135 5.142959 2.120980
H -3.726710 5.817160 0.285641
C -2.329751 2.770528 2.222935
C -2.316922 4.037648 2.822092
H -2.816453 6.138418 2.576003
H -1.938647 1.924850 2.798321
H -1.924887 4.146256 3.838766
C 3.091069 -0.492597 3.784261
H 3.925082 -0.375389 4.490020
H 2.283280 0.216919 4.030813
H 2.655840 -1.501625 3.881139
C 5.113359 -4.003316 -0.415846
H 4.578116 -4.509833 0.405941
H 4.723355 -4.407207 -1.363363
H 6.189058 -4.210074 -0.325670
C 3.576015 4.121715 0.243237
H 3.270253 4.530483 -0.734346
H 2.729500 4.272583 0.935391
H 4.458256 4.660127 0.616009
C 5.643331 0.651666 -4.026834
H 5.088268 0.008377 -4.726228
H 5.438612 1.702474 -4.297515
H 6.724899 0.473558 -4.114009
H -1.343135 0.622561 -3.589805
C -6.021509 -0.995511 1.782331
H -6.268866 -1.622312 2.682896
C -6.083611 -1.786823 0.489105
H -7.074153 -2.286563 0.458792
H -5.352591 -2.614042 0.599373
C -5.764686 -1.063224 -0.794041
C -4.702863 -1.505656 -1.622530
C -6.492877 0.081899 -1.165197
C -4.412068 -0.798306 -2.806075
C -6.184502 0.788428 -2.334139
H -7.292469 0.437624 -0.507946
C -5.136965 0.345795 -3.156470
H -3.584981 -1.124817 -3.446121
H -6.751288 1.688083 -2.595214
H -4.862621 0.905215 -4.056324
O -5.721193 0.181229 1.908438
C -0.602342 -2.880133 0.563250
O -0.862111 -3.897581 -0.092681
N -1.079747 -2.540854 1.744025
O -0.583854 -1.200521 2.132337
S -0.913590 -0.921282 3.733623

O -0.527485 0.511865 3.834517
O -0.291064 -1.924786 4.608111
C -2.714711 -1.114939 3.799919
H -3.210852 -0.175904 3.511367
H -2.966403 -1.923515 3.093783
H -2.970047 -1.395132 4.831914
K 0.421696 1.156503 0.746261
H 0.171157 0.658044 -2.697600
O 0.369560 -1.854991 0.005192
C 0.504263 -1.587707 -1.287858
O 1.274311 -0.640586 -1.597495
C -0.250549 -2.338778 -2.346476
H 0.158239 -3.361565 -2.411589
H -1.313199 -2.459021 -2.074551
H -0.120316 -1.832952 -3.313977
I -2.722317 0.669544 0.088292
I -3.432038 -2.997954 -0.979951

Egas = -3753.32846106
No imaginary frequency
Zero-point correction= 0.624160
Sum of electronic and thermal Enthalpies= -3752.639542
Sum of electronic and thermal Free Energies= -3752.812638
Gsolv= -3753.792742

2-(2-iodophenyl)acetaldehyde

C -0.089977 2.152658 -0.264367
C -0.058268 0.773523 0.017884
C 1.182935 0.164072 0.326482
C 2.352297 0.947863 0.328988
C 2.311393 2.316481 0.046841
C 1.080732 2.917741 -0.250686
H -1.037684 2.647033 -0.499112
H 3.306421 0.462315 0.561961
H 3.231662 2.908735 0.057969
H 1.026866 3.987935 -0.473398
C 1.290584 -1.315258 0.626016
C 1.997893 -2.070066 -0.488592
H 0.258466 -1.706415 0.692431
H 1.807455 -1.519793 1.578603
O 2.937247 -2.830276 -0.323185
H 1.584221 -1.870148 -1.515608
I -1.775112 -0.331878 0.002401

Egas = -394.16260959
No imaginary frequency
Zero-point correction= 0.123897
Sum of electronic and thermal Enthalpies= -393.940851
Sum of electronic and thermal Free Energies= -393.990638
Gsolv= -394.015490

2-Iodophenethyl carbamate (primary carbamate of E)

N 2.859862 0.460162 1.671675
C 2.393302 1.008702 0.506605
O 2.968593 0.970162 -0.575893
O 1.217622 1.670125 0.761160
C 0.679117 2.423758 -0.346723
C -0.278807 1.613166 -1.236019
H 1.510753 2.815014 -0.955720
H 0.143265 3.258775 0.133310
H -0.710300 2.336205 -1.953828
H 0.310535 0.900112 -1.833357
C -1.394784 0.914608 -0.492574
C -1.260698 -0.408327 -0.006169
C -2.603148 1.600918 -0.268278
C -2.340755 -1.003135 0.674143
C -3.668567 1.009226 0.417808
H -2.703050 2.623582 -0.650472
C -3.532967 -0.303800 0.887643
H -2.258731 -2.029360 1.046606

H -4.598157 1.564931 0.576871
H -4.356925 -0.790641 1.418888
H 2.276028 0.441873 2.503421
H 3.661344 -0.159469 1.616305
I 0.476610 -1.442173 -0.300085

Egas = -564.779661384
No imaginary frequency
Zero-point correction= 0.175133
Sum of electronic and thermal Enthalpies= -564.590801
Sum of electronic and thermal Free Energies= -564.645407
Gsolv= -564.905775

EtOAc

O	0.056762	-0.776094	-0.256006
C	-0.902919	0.177255	-0.062544
O	-0.712587	1.378406	-0.179920
C	1.376471	-0.272495	-0.602495
H	1.842334	-1.097056	-1.165031
H	1.258369	0.603706	-1.260524
C	2.176859	0.081407	0.643774
H	3.200322	0.383243	0.361444
H	1.704825	0.921481	1.177478
H	2.246949	-0.782325	1.325536
C	-2.216080	-0.473170	0.319236
H	-3.002649	0.291764	0.364386
H	-2.488126	-1.255612	-0.407660
H	-2.121412	-0.961665	1.303948

Egas = -307.331606338
No imaginary frequency
Zero-point correction= 0.115890
Sum of electronic and thermal Enthalpies= -307.207349
Sum of electronic and thermal Free Energies= -307.247710
Gsolv= -307.346279

TS”$_E$

Rh	1.560853	0.103761	-0.235227
Rh	3.709986	-0.634535	0.602068
O	2.454249	0.357698	-2.063606
O	4.505048	-0.277667	-1.248105
O	0.790955	-0.218201	1.669487
O	2.828659	-0.954528	2.428412
O	2.096005	2.007526	0.326309
O	4.131331	1.296528	1.122309
O	1.122492	-1.855141	-0.723869
O	3.178581	-2.543368	0.024843
C	3.716007	0.141909	-2.170548
C	3.247397	2.192801	0.861840
C	1.587126	-0.680901	2.571060
C	2.029276	-2.745565	-0.498029
N	-0.277418	0.692140	-1.060466
C	-0.659713	1.685731	-1.922119
O	-0.006743	2.676822	-2.214005
O	-1.941362	1.431452	-2.482580
C	-2.914902	2.467706	-2.343988
C	-4.013561	2.021680	-1.350392
H	-3.378180	2.631265	-3.333191
H	-2.423118	3.406985	-2.032830
H	-4.858851	2.725334	-1.461643
H	-4.393087	1.039356	-1.693937
C	-3.627278	1.980655	0.117579
C	-4.153193	2.979662	0.965419
C	-2.764876	1.025159	0.715257
C	-3.851682	3.037672	2.330550
H	-4.821133	3.731186	0.529806
C	-2.441260	1.087858	2.083722
C	-2.989393	2.088766	2.895209
H	-4.282573	3.831751	2.948713
H	-1.720451	0.380575	2.503877
H	-2.727620	2.133760	3.956958

C	4.325719	0.431783	-3.525757	H	-4.363132	-1.143195	-1.617121
H	3.713013	-0.024691	-4.319485	C	-5.464046	-2.069601	-1.258297
H	4.325069	1.521883	-3.697105	H	-4.968918	-3.052494	-1.270569
H	5.357108	0.054819	-3.574903	C	-6.616657	-1.912290	-2.215180
C	3.607346	3.626130	1.193069	H	-6.275389	-2.120342	-3.241817
H	3.964430	4.128169	0.276932	H	-7.024246	-0.889195	-2.182517
H	2.717902	4.169958	1.546119	O	-5.896248	-1.745596	0.066634
H	4.406835	3.658673	1.947482	C	-4.950895	-1.782755	1.041294
C	1.703510	-4.160657	-0.930052	O	-3.772749	-2.065626	0.829802
H	0.633561	-4.373316	-0.780038	I	-1.791650	-0.451015	-0.406685
H	1.922326	-4.270479	-2.006866	C	-5.513495	-1.400995	2.386119
H	2.319107	-4.882749	-0.374423	H	-5.101156	-0.415226	2.665866
C	0.989326	-0.895058	3.948212	H	-6.610976	-1.347608	2.375804
H	0.895860	0.077840	4.462269	H	-5.172336	-2.126067	3.141586
H	-0.018706	-1.332299	3.863517	H	-7.429682	-2.618193	-1.968066
H	1.635732	-1.549414	4.550401				

Egas = -2005.18049171
Zero-point correction= 0.468685
One imaginary frequency -125.2282
Sum of electronic and thermal Enthalpies= -2004.666836
Sum of electronic and thermal Free Energies= -2004.795076
Gsolv= -2005.403931

INT”$_E$

Rh	-1.406385	-0.536302	0.098290	H	2.592866	-4.328349	-1.639361
Rh	-3.411999	0.832139	0.151357	H	3.483859	-4.604322	-0.095231
O	-2.382871	-1.792817	-1.193947	H	4.773051	-3.026347	-1.485238
O	-4.291938	-0.515471	-1.112418	H	3.328902	-2.048287	-1.800617
O	-0.551443	0.802911	1.434831	C	4.167458	-1.815615	0.195519
O	-2.417441	2.137505	1.395035	C	5.218710	-2.245524	1.033080
O	-2.148523	-1.581051	1.699481	C	3.449666	-0.663492	0.602280
O	-4.033521	-0.271124	1.761717	C	5.540570	-1.584832	2.222874
O	-0.776897	0.640600	-1.508380	H	5.790313	-3.131854	0.734769
O	-2.695974	1.891473	-1.454920	C	3.758236	-0.002419	1.806255
C	-3.599619	-1.524116	-1.504970	C	4.804129	-0.460062	2.616086
C	-3.289248	-1.227293	2.179526	H	6.362450	-1.952479	2.845433
C	-1.215988	1.865483	1.736826	H	3.176965	0.868051	2.123556
C	-1.538660	1.604886	-1.910543	H	5.036597	0.059093	3.550882
N	0.353597	-1.739870	0.269728	C	-4.292912	-2.522691	-2.408121
C	0.892678	-2.670291	-0.583564	H	-3.565027	-2.984762	-3.091642
O	0.740959	-2.740473	-1.800476	H	-4.736144	-3.323406	-1.790199
O	1.822203	-3.472764	0.107101	H	-5.100465	-2.033544	-2.972492
C	2.937281	-3.862462	-0.699705	C	-3.813882	-2.052830	3.335666
C	3.839915	-2.646676	-1.029599	H	-4.326908	-2.946590	2.939001

H	-2.983318	-2.394722	3.971672
H	-4.536923	-1.470146	3.924895
C	-0.974969	2.483086	-3.006910
H	-0.190821	3.128749	-2.572355
H	-0.513539	1.862066	-3.791906
H	-1.762804	3.116244	-3.438901
C	-0.464258	2.910338	2.535467
H	0.225847	2.426496	3.244712
H	0.132383	3.516022	1.829107
H	-1.161819	3.573301	3.067348
C	3.208575	1.673182	-1.425812
H	2.524940	2.156279	-2.146019
C	4.438076	1.095597	-2.095100
H	5.067831	0.532364	-1.386480
O	3.633144	2.647935	-0.464700
C	2.701151	3.591209	-0.102350
O	1.564607	3.619485	-0.552659
I	1.867697	0.142493	-0.524148
C	3.258316	4.520128	0.947201
H	2.765356	5.499509	0.866862
H	3.030029	4.104768	1.945638
H	4.349816	4.625628	0.862427
H	5.044262	1.912793	-2.524602
H	0.562650	-1.965245	1.251844
H	4.144029	0.419620	-2.915437

Egas = -2005.22290278
Zero-point correction= 0.476510
No imaginary frequency
Sum of electronic and thermal Enthalpies= -2004.702258
Sum of electronic and thermal Free Energies= -2004.825038
Gsolv= -2005.413429

Protonation TS$_E$

Rh	1.583959	-0.353804	0.383495
Rh	3.543952	0.386387	-0.852628
O	2.176382	0.876744	1.923035
O	4.069363	1.507950	0.782493
O	1.100763	-1.548778	-1.227142
O	2.904767	-0.781000	-2.421899
O	2.749265	-1.881794	1.106789
O	4.582416	-1.191722	-0.084021
O	0.578679	1.286510	-0.502084
O	2.473201	1.947562	-1.610249
C	3.291485	1.505002	1.803662
C	3.974633	-1.974085	0.729523
C	1.844366	-1.484194	-2.278331
C	1.239740	2.055601	-1.307548
N	0.082325	-1.377467	1.452242
C	-0.944046	-1.037259	2.194560
O	-1.096633	0.051561	2.859583
O	-1.961405	-1.981096	2.224190
C	-3.191801	-1.579450	2.840966
C	-4.049249	-0.632185	1.974800
H	-2.988027	-1.090303	3.808503
H	-3.729950	-2.525469	3.016446
H	-4.953586	-0.420012	2.577682
H	-3.528679	0.332986	1.881763
C	-4.499322	-1.175402	0.631132
C	-5.734058	-1.857811	0.585378
C	-3.770372	-1.036451	-0.580842
C	-6.250710	-2.392017	-0.599460
H	-6.300481	-1.965657	1.518092
C	-4.305589	-1.560072	-1.777643
C	-5.529875	-2.236623	-1.790242
H	-7.211499	-2.916371	-0.592503
H	-3.760530	-1.446006	-2.720443
H	-5.918949	-2.636000	-2.732115
C	3.747081	2.302437	3.008285
H	4.412396	1.672495	3.624655
H	4.316990	3.187863	2.688790
H	2.885492	2.596072	3.625821
C	4.779558	-3.103519	1.335354
H	4.140157	-3.983453	1.501867
H	5.628113	-3.359304	0.684039
H	5.175349	-2.781435	2.314560
C	0.478818	3.205714	-1.938666
H	-0.434134	2.843991	-2.443358
H	0.169809	3.924938	-1.159781
H	1.115964	3.725984	-2.667340

C	1.411343	-2.345418	-3.446153
H	1.917095	-2.027647	-4.369047
H	1.670083	-3.398904	-3.241245
H	0.317205	-2.289169	-3.566475
C	-2.101984	2.533908	0.631985
H	-1.680769	2.813962	-0.334320
C	-1.362116	2.433578	1.826727
H	-1.899751	2.763111	2.729191
O	-3.454196	2.579937	0.711299
C	-4.160853	2.770434	-0.509298
O	-3.598210	3.081803	-1.535546
I	-1.904940	-0.120979	-0.669364
C	-5.612080	2.467042	-0.292724
H	-6.203288	2.857442	-1.131687
H	-5.728426	1.368204	-0.245666
H	-5.967532	2.880557	0.664004
H	-0.328880	2.793734	1.735054
H	-0.051777	-2.297277	1.013434
H	-1.157760	1.226774	2.181291

Egas = -2005.18589743
One imaginary frequency -737.8958
Zero-point correction= 0.470895
Sum of electronic and thermal Enthalpies= -2004.671182
Sum of electronic and thermal Free Energies= -2004.794272
Gsolv= -2005.403392

Product complex of hydride transfer from EtOAc to substrate E

Rh	-1.237953	-0.152416	0.408696
Rh	-3.295929	-0.196078	-0.854735
O	-0.631519	-1.758313	-0.766343
O	-2.473149	-1.570754	-2.122117
O	-2.028273	1.362463	1.549457
O	-4.031310	1.210979	0.444402
O	-2.076504	-1.514171	1.701672
O	-3.946856	-1.681164	0.393302
O	-0.607375	1.285332	-0.959435
O	-2.590662	1.265885	-2.098643
C	-1.325286	-2.053243	-1.806871
C	-3.234251	-1.997719	1.408801
C	-3.258453	1.686514	1.349616
C	-1.382930	1.648800	-1.925896
N	0.321696	-0.184411	2.148397
C	1.465364	-1.024945	2.091964
O	2.608966	-0.668975	2.344406
O	1.098379	-2.255818	1.680576
C	2.179914	-3.174969	1.383730
C	2.680636	-3.040207	-0.071335
H	1.719508	-4.163902	1.530414
H	2.996063	-3.031537	2.110861
H	3.007131	-4.053525	-0.373190
H	1.798868	-2.807587	-0.693474
C	3.856026	-2.102596	-0.303618
C	5.145325	-2.647930	-0.128796
C	3.753258	-0.743235	-0.693849
C	6.308141	-1.892841	-0.309006
H	5.229099	-3.702932	0.159353
C	4.929225	0.012587	-0.882763
C	6.195577	-0.552147	-0.696522
H	7.290811	-2.351825	-0.160361
H	4.864197	1.062586	-1.188395
H	7.090998	0.057654	-0.855179
C	-0.703929	-3.049335	-2.761342
H	0.190035	-2.590050	-3.221064
H	-0.367047	-3.941857	-2.209295
H	-1.415673	-3.336884	-3.547743
C	-3.807949	-3.013327	2.371399
H	-3.016965	-3.689605	2.730510
H	-4.228831	-2.489460	3.247627
H	-4.611643	-3.585862	1.886663
C	-0.780757	2.572507	-2.958963
H	-0.026641	3.230542	-2.500450
H	-0.266994	1.957477	-3.720827
H	-1.565003	3.158813	-3.459840
C	-3.849360	2.707953	2.296434
H	-4.280261	2.185871	3.169081
H	-3.067845	3.391961	2.659369
H	-4.654911	3.268727	1.799940
C	1.437404	4.217661	-0.087711
H	0.506964	3.736555	0.227573

C	1.555307	5.161702	-1.030380	H	3.621471	1.351335	2.433719
H	2.525207	5.588228	-1.299278	H	4.524116	2.551036	1.422174
O	2.575405	3.751675	0.557453	H	3.957248	2.990055	3.065113
C	2.394836	2.882714	1.611138	H	0.664749	5.527042	-1.546039
O	1.287003	2.581157	2.039832	H	0.595228	0.784799	2.381458
I	1.924201	0.232130	-0.867358	H	-0.405668	-0.555864	2.773043
C	3.715756	2.412407	2.154756				

Egas = -2005.24278416
No imaginary frequency
Zero-point correction= 0.477065
Sum of electronic and thermal Enthalpies= -2004.720857
Sum of electronic and thermal Free Energies= -2004.846466
Gsolv= -2005.449050

Vinyl acetate

O	0.248864	-0.999651	-0.317027	H	2.775482	0.881202	0.808524
C	-0.743477	0.024057	-0.206659	H	1.063324	1.198897	0.952242
O	-0.546840	1.150281	-0.732513	C	-2.052793	-0.251736	0.555726
C	1.544732	-0.400526	-0.398730	H	-2.412778	0.658776	0.987327
H	2.287254	-1.166517	-0.481313	H	-2.784963	-0.639353	-0.121459
C	1.805846	0.432906	0.869659	H	-1.870357	-0.966254	1.331021

Egas = -306.101958402
Zero-point correction= 0.092102
No imaginary frequency
Sum of electronic and thermal Enthalpies= -306.001962
Sum of electronic and thermal Free Energies= -306.041124
Gsolv= -306.106432

S5-10. Hydrogen transfer to **^{3}NR$_{A}$** (Table S2-3)

H_2O

O 0.000000 0.000000 0.121830
H 0.000000 0.763376 -0.487321
H 0.000000 -0.763376 -0.487321

Egas = -76.3224807687
No imaginary frequency
Zero-point correction= 0.020650
Sum of electronic and thermal Enthalpies= -76.298052
Sum of electronic and thermal Free Energies= -76.319524
Gsolv= -76.375478

CH_2Cl_2

C 0.000000 0.000000 0.764709
H -0.906187 0.000000 1.383214
H 0.906187 0.000000 1.383214
Cl 0.000000 1.498015 -0.216314
Cl 0.000000 -1.498015 -0.216314

Egas = -959.236420092
No imaginary frequency
Zero-point correction= 0.028854
Sum of electronic and thermal Enthalpies= -959.203026
Sum of electronic and thermal Free Energies= -959.233755
Gsolv= -959.3079253

Hydrogen transfer TS from H_2O to ^3NR_A

Rh -0.802018 -0.062375 -0.157896
Rh -3.114036 0.256883 0.428029
O -0.462933 -0.479720 1.821537
O -2.667496 -0.185914 2.366948
O -1.310835 0.404474 -2.097226
O -3.507273 0.693340 -1.522898
O -0.503911 1.931200 0.208777
O -2.702283 2.223020 0.763794
O -1.267748 -2.029488 -0.515906
O -3.456534 -1.719132 0.074448
C -1.445951 -0.469514 2.649325
C -1.501545 2.647348 0.589341
C -2.535556 0.694174 -2.363150
C -2.468785 -2.440972 -0.319899
N 1.059780 -0.334762 -0.617183
C 1.916407 -1.378009 -0.347525
O 1.656566 -2.342106 0.379308
O 3.103502 -1.201293 -0.999739
C 4.134869 -2.173456 -0.714076
C 5.462643 -1.579769 -1.193083
H 3.896727 -3.111303 -1.249755
H 4.143663 -2.387934 0.368292
H 6.213875 -2.390483 -1.162811
H 5.347297 -1.292957 -2.254928
C 5.943776 -0.401558 -0.366926
C 5.343533 0.869914 -0.482833
C 6.992746 -0.571140 0.557290
C 5.787102 1.935660 0.313371
H 4.513585 1.041025 -1.180267
C 7.437589 0.496053 1.350602
H 7.470409 -1.554178 0.653235
C 6.833396 1.755576 1.230561
H 5.305902 2.913972 0.209334
H 8.258200 0.342342 2.059819
H 7.178069 2.592699 1.847172
H 2.699820 1.527458 -2.984957
O 2.564044 2.062217 -2.166926
H 1.775822 0.794984 -1.324906
C -2.840013 1.087705 -3.788128
H -2.435061 2.096802 -3.977309
H -2.342973 0.395691 -4.485590
H -3.925380 1.095051 -3.960463
C -2.726436 -3.905260 -0.579893
H -2.469992 -4.145922 -1.624707
H -2.071777 -4.512557 0.066580
H -3.779947 -4.150301 -0.386403
C -1.217577 4.100990 0.874578
H -0.766954 4.189091 1.878511
H -0.491359 4.492387 0.146198
H -2.148803 4.684768 0.851147
C -1.122265 -0.841810 4.075707
H -0.890237 -1.919454 4.123178
H -0.226001 -0.297215 4.411696
H -1.975301 -0.621839 4.732712

Egas = -1763.41544062
One imaginary frequency of -1982.6765

Zero-point correction= 0.384518
Sum of electronic and thermal Enthalpies= -1762.993288
Sum of electronic and thermal Free Energies= -1763.106248
Gsolv= -1763.583343

Hydrogen transfer TS from CH_2Cl_2 to ^3NR_A

Rh -0.572081 -0.769572 0.034209
Rh -2.300317 -2.441924 0.010562
O -1.748358 0.312387 1.324902
O -3.406375 -1.267983 1.258288
O 0.520651 -1.980507 -1.226235
O -1.130669 -3.567803 -1.236328
O -1.518469 0.096630 -1.579997
O -3.141502 -1.515914 -1.596373
O 0.260735 -1.766676 1.627811
O -1.408188 -3.330560 1.615501
C -2.899077 -0.154903 1.656034
C -2.581571 -0.453857 -2.051819
C 0.018386 -3.111346 -1.580523
C -0.320842 -2.823919 2.072992
N 0.970588 0.512052 0.034653
C 1.122238 1.866505 0.137784
O 2.224822 2.438298 0.206138
O -0.072617 2.522781 0.151846
O -0.006847 3.958948 0.309873
C -0.952640 4.636290 -0.689396
H 1.033286 4.283291 0.148670
H -0.299229 4.190326 1.349990
H -0.716008 5.716464 -0.673160
H -0.692974 4.270820 -1.700451
C -2.431836 4.442901 -0.418455
C -3.054731 3.197831 -0.641950
C -3.215961 5.505426 0.068542
C -4.425029 3.029407 -0.393064
H -2.450324 2.357760 -0.997633
C -4.585645 5.338714 0.322807
H -2.747555 6.481152 0.245259
C -5.196953 4.099075 0.089680
H -4.896497 2.057439 -0.582321
H -5.176063 6.181641 0.697471
H -6.267610 3.967042 0.277838
C -3.728587 0.700744 2.582624
H -3.119409 1.015847 3.444826
H -4.040237 1.613869 2.046247
H -4.615932 0.149285 2.923830
C -3.221590 0.221186 -3.241816
H -4.077748 -0.366668 -3.600825
H -3.557945 1.233071 -2.958112
H -2.478256 0.336658 -4.047499
C 0.879604 -3.982460 -2.465078
H 1.372227 -3.369893 -3.235884
H 1.671049 -4.449942 -1.853805
H 0.274172 -4.773439 -2.929702
C 0.355419 -3.534430 3.220906
H 1.230727 -4.088032 2.839111
H 0.720426 -2.801719 3.956970
H -0.338253 -4.243989 3.693603
H 1.886470 0.048033 -0.006176
C 5.566515 0.073776 -0.481527
H 3.383956 1.249095 -2.038728
Cl 6.113151 0.866846 -1.996763
Cl 4.299854 -1.160606 -0.840969
C 5.045270 1.098117 0.549452
H 4.127939 1.616554 0.219217
Cl 4.629170 0.229657 2.074371
Cl 6.308985 2.331942 0.878099
H 6.430918 -0.468233 -0.073380

Egas = -3605.57923665
One imaginary frequency of -10.9036
Zero-point correction= 0.421636
Sum of electronic and thermal Enthalpies= -3605.113176
Sum of electronic and thermal Free Energies= -3605.243773
Gsolv= -3605.831904

Hydrogen transfer TS from HOAc to ^3NR_A (HOAc coordinated to catalyst)

Rh -0.209752 -0.835700 -0.328830
Rh -2.045941 0.259962 0.812943
O -0.623897 -2.508816 0.787075
O -2.404492 -1.486741 1.796738
O 0.079768 0.844347 -1.460923
O -1.226264 2.006433 -0.015929
O 0.976983 -0.094773 1.184006
O -0.796213 0.791959 2.329555
O -1.428682 -1.491325 -1.818826
O -3.210111 -0.667671 -0.664199
C -1.629694 -2.493962 1.581381
C 0.457395 0.527776 2.174802
C -0.477211 1.928563 -1.042343
C -2.686491 -1.254645 -1.666590
N 1.358428 -1.507997 -1.331605
C 2.629042 -1.802166 -0.883474
O 2.852450 -2.349873 0.195584
O 3.588269 -1.449523 -1.806438
C 4.956295 -1.704352 -1.419078
C 5.766913 -0.403529 -1.516869
H 5.356307 -2.458684 -2.120164
H 4.961571 -2.116696 -0.396753
H 6.826483 -0.667230 -1.339828
H 5.700115 -0.025244 -2.553210
C 5.323847 0.665744 -0.539843
C 4.301465 1.572431 -0.881132
C 5.904670 0.761170 0.739366
C 3.873286 2.549411 0.028785
H 3.840189 1.510064 -1.872714
C 5.481915 1.737508 1.652073
H 6.703421 0.064403 1.020194
C 4.464714 2.637662 1.298445
H 3.081866 3.250216 -0.258945
H 5.955266 1.802236 2.637604
H 4.145843 3.413691 2.002806
C -1.950275 -3.770920 2.317884
H -1.040270 -4.375465 2.442289
H -2.405483 -3.543964 3.293219
H -2.678167 -4.352689 1.725638
C 1.396207 0.963885 3.271811
H 1.692965 0.080066 3.862462
H 2.310721 1.386973 2.826103
H 0.906845 1.694422 3.931287
C -0.246365 3.185246 -1.838352
H 0.621996 3.083373 -2.504762
H -1.157381 3.362033 -2.435993
H -0.116902 4.041963 -1.158826
C -3.609423 -1.683929 -2.778340
H -3.108872 -2.391862 -3.453875
H -4.523644 -2.130529 -2.357499
H -3.903636 -0.780413 -3.339960
O -3.728987 1.569834 0.597180
C -3.999673 2.065083 -0.602587
O -3.342574 1.870227 -1.637700
C -5.248947 2.945979 -0.578965
H -5.450614 3.323416 -1.592354
H -6.115793 2.369387 -0.215045
H -5.104269 3.793179 0.112359
H 1.362602 -0.702805 -2.508711

Egas = -1915.9338090
One imaginary frequency of -659.0725
Zero-point correction= 0.423228
Sum of electronic and thermal Enthalpies= -1915.470393
Sum of electronic and thermal Free Energies= -1915.588193
Gsolv= -1916.088031

Hydrogen transfer TS from HOAc to ^3NR_A (HOAc not coordinated to catalyst)

Rh 0.475656 0.114106 -0.129036
Rh 0.889458 -2.237080 0.219295
O -1.029756 -0.022390 1.258858
O -0.638215 -2.257504 1.562721
O 2.021688 0.104606 -1.464840
O 2.434091 -2.116119 -1.098054
O -0.821329 -0.425015 -1.635407
O -0.411332 -2.650096 -1.289996

O 1.806461 0.466771 1.380903	H -6.381833 -1.916701 0.760529
O 2.211728 -1.763098 1.684399	C -2.423307 -1.218142 2.773364
C -1.276830 -1.164007 1.795313	H -2.337741 -0.391592 3.496508
C 2.694938 -0.975596 -1.637281	H -3.370546 -1.081364 2.222778
C 2.413660 -0.517265 1.939796	H -2.435461 -2.184416 3.296803
N 0.317308 2.067281 -0.358235	C -1.925693 -2.009473 -3.030240
C -0.683364 2.985417 -0.108149	H -1.978813 -3.097375 -3.174925
O -0.491175 4.147589 0.255179	H -2.930755 -1.610539 -2.813130
O -1.916170 2.439604 -0.334767	H -1.574708 -1.525007 -3.956459
C -3.029791 3.317396 -0.051192	C 3.915467 -0.866724 -2.511758
C -4.250253 2.831107 -0.837796	H 3.669759 -0.327618 -3.440242
H -2.755391 4.345717 -0.340513	H 4.658962 -0.264895 -1.960313
H -3.220760 3.305119 1.037720	H 4.324111 -1.861391 -2.737981
H -5.021164 3.619009 -0.752062	C 3.472424 -0.162700 2.948734
H -3.966071 2.781363 -1.905481	H 4.311208 0.295345 2.396310
C -4.833382 1.506041 -0.385440	H 3.085716 0.588603 3.655215
C -4.176522 0.290006 -0.663544	H 3.813288 -1.059159 3.485209
C -6.050461 1.462755 0.320627	H 1.561156 2.713876 -0.241567
C -4.730153 -0.931483 -0.252509	O 3.663295 3.294531 -0.169523
H -3.218917 0.310808 -1.192709	C 4.690443 2.560648 -0.019408
C -6.604660 0.242911 0.736765	O 4.570742 1.295288 0.087207
H -6.576408 2.399401 0.541279	C 6.066576 3.196689 0.000627
C -5.947431 -0.961226 0.448118	H 6.006757 4.222610 0.397769
H -4.211714 -1.869723 -0.484084	H 6.762918 2.594781 0.606584
H -7.555753 0.234115 1.279648	H 6.462664 3.249378 -1.028607

Egas = -1915.93453507
One imaginary frequency of -897.1778
Zero-point correction= 0.422481
Sum of electronic and thermal Enthalpies= -1915.471380
Sum of electronic and thermal Free Energies= -1915.590335
Gsolv= -1916.097972

Hydrogen transfer TS from Salt-K$_A$ to ^{3}NR$_A$

Rh	1.793642	-0.548862	-0.059188	C	3.023776	0.393575	-2.579901
Rh	3.930915	0.552240	0.159546	C	1.863448	2.641510	0.169276
O	1.928234	-0.108852	-2.140702	C	2.785155	-0.307881	2.691218
O	4.034063	0.757671	-1.875539	C	4.377194	-2.300785	-0.126806
O	1.790010	-0.792851	2.039913	N	0.111788	-1.643436	-0.158037
O	3.795634	0.303531	2.190435	C	-0.464639	-2.307245	-1.198375
O	0.966089	1.755863	0.004532	O	-0.539713	-1.839267	-2.351840
O	3.124211	2.412183	0.303509	O	-1.024170	-3.523585	-0.856573
O	3.126339	-2.457743	-0.218442	C	-1.777315	-4.170123	-1.902879
O	4.987482	-1.167186	0.030563	C	-3.281695	-3.853647	-1.808605

H	-1.610740	-5.249661	-1.751913
H	-1.369178	-3.863650	-2.880299
H	-3.795871	-4.526327	-2.522155
H	-3.634587	-4.124304	-0.797479
C	-3.640336	-2.412243	-2.109115
C	-3.739611	-1.957419	-3.438817
C	-3.859581	-1.490812	-1.066399
C	-4.040583	-0.618899	-3.723473
H	-3.575264	-2.663258	-4.261688
C	-4.164659	-0.147892	-1.345045
H	-3.799961	-1.828522	-0.026490
C	-4.254800	0.294599	-2.676293
H	-4.122741	-0.289032	-4.765117
H	-4.349735	0.550914	-0.521833
H	-4.514082	1.337255	-2.885549
C	3.158343	0.540808	-4.082641
H	3.441392	-0.435806	-4.513006
H	2.195105	0.831322	-4.530827
H	3.936767	1.275609	-4.334171
C	1.464871	4.106659	0.246363
H	0.476799	4.261847	-0.211756
H	1.413502	4.407795	1.307187
H	2.222860	4.736821	-0.244101
C	5.274753	-3.523578	-0.206294
H	4.994599	-4.236280	0.587069
H	5.116995	-4.031088	-1.172582
H	6.333971	-3.248214	-0.101364
C	2.751558	-0.434540	4.201646
H	2.148602	0.389822	4.622984
H	2.285854	-1.387142	4.498007
H	3.767966	-0.362953	4.615253
H	0.014695	-2.575582	1.029032
C	-1.381183	0.093132	2.546811
H	-0.326692	0.420269	2.690036
C	-1.684775	-1.276703	3.093388
H	-1.487295	-1.173059	4.185244
H	-0.864112	-1.931388	2.739371
C	-3.041608	-1.887351	2.838849
C	-3.129781	-3.202408	2.343790
C	-4.232008	-1.189897	3.125745
C	-4.377583	-3.812334	2.146309
H	-2.211835	-3.749746	2.101081
C	-5.479015	-1.795150	2.917508
H	-4.183164	-0.165749	3.507584
C	-5.556759	-3.108759	2.430706
H	-4.425658	-4.839429	1.769135
H	-6.394617	-1.237604	3.140356
H	-6.532018	-3.580937	2.274340
O	-2.198270	0.859090	2.029346
C	-1.241386	2.938486	2.140660
O	-0.305764	2.719290	2.840356
N	-2.033859	3.736495	1.548971
O	-3.188295	3.209392	0.898280
S	-3.229892	3.755080	-0.712005
O	-4.555333	3.312189	-1.165894
O	-1.994443	3.335445	-1.413490
C	-3.188049	5.543136	-0.500776
H	-4.123645	5.848817	-0.011964
H	-2.315030	5.793510	0.118455
H	-3.102724	5.983231	-1.505244
K	-0.875443	0.739529	-1.699204

Egas = -3502.98333236
One imaginary frequency of -1047.5441
Zero-point correction= 0.570960
Sum of electronic and thermal Enthalpies= -3502.354271
Sum of electronic and thermal Free Energies= -3502.515210
Gsolv= -3503.277079

S5-11. Hydrogen transfer to **^{3}NR$_{E}$** (Table S2-4)

^{3}NR$_{E}$

Rh 1.050880 0.266706 -0.470123
Rh 2.893594 -0.305361 0.985866
O -0.128569 0.230991 1.217956
O 1.631110 -0.295761 2.585119

O 2.392204 0.276616 -2.031480
O 4.122645 -0.287390 -0.643703
O 0.779621 -1.759777 -0.781625
O 2.470521 -2.284199 0.670243
O 1.521954 2.234405 -0.105676
O 3.263141 1.679606 1.279903
C 0.399745 -0.007374 2.364510
C 1.510802 -2.590744 -0.119313
C 3.625937 0.003715 -1.790316
C 2.508129 2.518506 0.667880
N -0.445810 0.815590 -1.538519
C -1.496901 1.654282 -1.279918
O -1.628404 2.377001 -0.287298
O -2.408807 1.570020 -2.308869
C -3.734004 2.064703 -2.030427
C -4.691499 0.934283 -1.595693
H -4.084182 2.488334 -2.985866
H -3.683278 2.863292 -1.271606
H -5.711761 1.317000 -1.786516
H -4.558711 0.085233 -2.290241
C -4.636266 0.496855 -0.142088
C -3.760344 -0.495111 0.371188
C -5.545288 1.103276 0.749680
C -5.610634 0.757764 2.102533
H -6.225583 1.867289 0.356072
C -4.755051 -0.235849 2.595374
H -3.177590 -1.624762 2.136180
H -6.330350 1.252562 2.762172
H -4.798502 -0.537579 3.646849
C -0.511678 0.100022 3.563535
H -0.378463 1.096940 4.019218
H -1.563249 -0.003248 3.260329
H -0.239496 -0.655980 4.315840
C 1.167512 -4.053543 -0.280731
H 0.311610 -4.295767 0.375321
H 0.863296 -4.262346 -1.318132
H 2.020566 -4.684713 0.006356
C 2.793904 3.989783 0.860589
H 3.096633 4.434425 -0.102284
H 1.876036 4.504994 1.186656
H 3.593722 4.131225 1.600805
C 4.560987 0.033749 -2.976486
H 4.191771 -0.650387 -3.757917
H 4.574311 1.048313 -3.408357
H 5.576727 -0.254679 -2.672230
I -2.335274 -1.406854 -0.793257
Egas = -1697.83525485

No imaginary frequency
Zero-point correction= 0.358052
Sum of electronic and thermal Enthalpies= -1697.441212
Sum of electronic and thermal Free Energies= -1697.548740
Gsolv= -1698.062574

TS Hydrogen transfer from H_2O to ^3NR_E

Rh -1.000393 -0.126831 0.409026
Rh -3.046768 0.489732 -0.719296
O -0.226536 1.692082 -0.143050
O -2.182895 2.287521 -1.184825
O -1.884967 -1.931150 0.871334
O -3.825814 -1.331474 -0.194998
O -0.383046 -0.934808 -1.380919
O -2.320852 -0.339379 -2.436312
O -1.810816 0.717398 2.097837
O -3.740091 1.297699 1.012343
C -0.979232 2.506552 -0.792039
C -1.161144 -0.881917 -2.404803
C -3.091028 -2.141387 0.478417
C -2.978341 1.246336 2.046170
N 0.557337 -0.716250 1.477839
C 1.513350 0.054842 2.062577
O 1.443930 1.235555 2.395825
O 2.700876 -0.711352 2.232774
C 3.877768 0.027616 2.595242
C 4.863233 0.131123 1.409853
H 4.354957 -0.521829 3.425491
H 3.573288 1.029121 2.942484
H 5.777568 0.611565 1.804433
H 5.163907 -0.887846 1.101472

C 4.347348 0.937495 0.238880
C 3.410943 0.425481 -0.697336
C 4.784651 2.266844 0.081178
C 2.895719 1.265219 -1.707746
C 4.314395 3.079573 -0.956064
H 5.512838 2.667526 0.795351
C 3.362758 2.575762 -1.854304
H 2.119953 0.897904 -2.387399
H 4.680665 4.106366 -1.054797
H 2.978336 3.202298 -2.665938
H 2.528214 -3.406659 1.452844
O 1.914887 -3.300524 0.689682
H 0.880026 -2.152029 1.429142
C -0.631893 -1.512027 -3.673791
H 0.235847 -0.935694 -4.039772
H -0.278128 -2.534324 -3.461109
H -1.409176 -1.532198 -4.450223
C -3.706677 -3.462733 0.880173
H -2.935163 -4.246065 0.918665
H -4.143402 -3.364272 1.889462
H -4.507989 -3.740101 0.179972
C -3.487926 1.876049 3.321421
H -3.370613 1.171564 4.160162
H -2.881664 2.767942 3.553970
H -4.542103 2.166631 3.211126
C -0.389479 3.866076 -1.090574
H 0.705604 3.795272 -1.168337
H -0.825783 4.279967 -2.011869
H -0.628712 4.549122 -0.256707
I 2.756384 -1.552056 -0.623626

Egas = -1774.12399933
One imaginary frequency -1414.7122
Zero-point correction= 0.376308
Sum of electronic and thermal Enthalpies= -1773.709743
Sum of electronic and thermal Free Energies= -1773.820175
Gsolv= -1774.367793

TS Hydrogen transfer from CH_2Cl_2 to ^3NR_E

Rh -1.085784 -0.814457 0.248493
Rh -3.290097 -1.569202 -0.370576
O -1.902122 1.062618 0.484066
O -3.975947 0.338149 -0.172124
O -0.437959 -2.757725 0.027343
O -2.535735 -3.465477 -0.559693
O -0.708238 -0.577916 -1.760174
O -2.839550 -1.179464 -2.318320
O -1.610561 -1.188350 2.198059
O -3.688381 -1.928046 1.589731
C -3.146069 1.239164 0.219208
C -1.660756 -0.766712 -2.607292
C -1.291089 -3.657537 -0.317415
C -2.773502 -1.670087 2.454412
N 0.799322 -0.451562 0.831375
C 1.359737 0.438496 1.699837
O 2.544379 0.400852 2.079765
O 0.478298 1.412010 2.070307
C 1.080000 2.548630 2.722885
C 1.689074 3.545621 1.717707
H 1.862353 2.197298 3.415559
H 0.258782 3.013650 3.290690
H 2.289925 4.254064 2.318563
H 2.414861 3.002574 1.087451
C 0.695617 4.348205 0.900358
C 0.173696 3.945295 -0.356040
C 0.276356 5.587350 1.428739
C -0.725693 4.797769 -1.035444
C -0.624523 6.421323 0.759958
H 0.685821 5.901614 2.396208
C -1.122643 6.022235 -0.486794
H -1.127861 4.508663 -2.012125
H -0.923280 7.377037 1.202008
H -1.816430 6.663226 -1.040271
C -3.679578 2.638235 0.414683
H -3.922817 2.785307 1.481648
H -2.911385 3.377167 0.138388
H -4.595275 2.784298 -0.176515
C -1.357247 -0.431548 -4.046648
H -2.016027 -0.994815 -4.723138

H -1.531176 0.648702 -4.205040
H -0.299521 -0.637283 -4.271534
C -0.768930 -5.071789 -0.418860
H 0.231608 -5.075088 -0.877968
H -0.672505 -5.494194 0.596507
H -1.462930 -5.696170 -0.999183
C -3.073675 -1.979861 3.901371
H -2.591794 -2.934690 4.174653
H -2.655889 -1.195719 4.550913
H -4.158231 -2.072800 4.055063
H 1.460383 -1.192433 0.569093
C 4.730901 -1.491124 -0.955257
H 3.580625 1.033368 -0.020580
Cl 5.752700 -0.078544 -1.377968
Cl 3.113918 -1.347228 -1.745816
C 4.572785 -1.643325 0.574466
H 4.028172 -0.798920 1.033441
Cl 3.610013 -3.131640 0.920330
Cl 6.185722 -1.769735 1.349884
H 5.215659 -2.382473 -1.377345
I 0.641435 2.133197 -1.225521

Egas = -3616.27168893
One imaginary frequency -10.0466
Zero-point correction= 0.411560
Sum of electronic and thermal Enthalpies=-3615.814477
Sum of electronic and thermal Free Energies= -3615.945875
Gsolv= -3616.616419

TS Hydrogen transfer from HOAc to $^{3}NR_{E}$ (HOAc coordinated to catalyst)

Rh	-0.717863	-0.966702	-0.247962
Rh	-2.781635	0.093480	0.469453
O	-1.889756	-2.154090	-1.447703
O	-3.835091	-1.255225	-0.641317
O	0.324353	0.295686	0.995303
O	-1.617653	1.204627	1.763847
O	-0.985987	-2.303032	1.291169
O	-2.856646	-1.209885	2.040935
O	-0.472064	0.397915	-1.763401
O	-2.633239	1.015394	-1.437937
C	-3.169436	-2.063432	-1.386883
C	-1.954687	-2.128316	2.110911
C	-0.349948	1.104232	1.736710
C	-1.521206	1.095971	-2.044976
N	1.052978	-1.645912	-0.789076
C	2.064088	-2.351370	-0.188027
O	2.070509	-2.953886	0.883028
O	3.183631	-2.234665	-1.019951
C	4.477243	-2.436814	-0.443784
C	5.301895	-1.140055	-0.594625
H	4.971871	-3.249497	-1.005172
H	4.371986	-2.745650	0.610805
H	6.360840	-1.416941	-0.438404
H	5.231710	-0.808782	-1.646313
C	4.965560	-0.031635	0.384369
C	3.983097	0.972995	0.197175
C	5.700249	-0.006178	1.590297
C	3.795084	1.966426	1.180841
C	5.504976	0.969496	2.570053
H	6.457824	-0.782147	1.747134
C	4.549969	1.971013	2.357556
H	3.045848	2.750313	1.036978
H	6.101712	0.953044	3.487278
H	4.388061	2.756395	3.102300
C	-3.962707	-2.973106	-2.293682
H	-3.437938	-3.930406	-2.428883
H	-4.970224	-3.134901	-1.883884
H	-4.062610	-2.492046	-3.282469
C	-2.035867	-3.075943	3.284053
H	-1.635647	-4.060874	3.002138
H	-1.415334	-2.678638	4.106212
H	-3.073253	-3.160478	3.638980
C	0.427697	2.034751	2.635410
H	1.446392	1.658083	2.804692
H	0.475546	3.023579	2.145415
H	-0.104674	2.161443	3.590383
C	-1.404065	2.108380	-3.156666
H	-0.546655	1.892727	-3.811396

H -2.334976 2.134466 -3.743621
H -1.277711 3.098087 -2.683431
O -3.928122 1.904010 0.651395
C -3.327280 3.055665 0.403236
O -2.150828 3.211823 0.035719
C -4.267614 4.241502 0.637818
H -3.768666 5.173933 0.334461
H -5.202522 4.111425 0.067872
H -4.539102 4.299571 1.705687
H 1.686800 -0.433163 -1.190870
I 2.784946 1.103131 -1.479186

Egas = -1926.62367368
One imaginary frequency -809.3086
Zero-point correction= 0.413593
Sum of electronic and thermal Enthalpies= -1926.168691
Sum of electronic and thermal Free Energies= -1926.288560
Gsolv= -1926.879088

TS Hydrogen transfer from HOAc to $^{3}NR_{E}$ (HOAc not coordinated to catalyst)

Rh -1.181037 -0.048530 0.192370
Rh -2.284835 -2.002533 -0.674348
O 0.340844 -0.439557 -1.139100
O -0.748492 -2.265432 -1.981569
O -2.749786 0.183660 1.476313
O -3.803705 -1.652565 0.630327
O -0.278026 -1.280395 1.582768
O -1.299188 -3.138555 0.711972
O -2.150330 1.022996 -1.260929
O -3.269631 -0.823925 -2.015351
C 0.233800 -1.438806 -1.939940
C -0.522575 -2.541804 1.543214
C -3.751939 -0.615066 1.388790
C -3.020229 0.437871 -2.002826
N -0.303140 1.546235 0.857138
C 0.903978 2.080233 0.472379
O 1.163908 2.432273 -0.683148
O 1.766211 2.180105 1.528536
C 3.099842 2.615466 1.180081
C 4.132190 1.778191 1.937365
H 3.201526 3.689511 1.416277
H 3.256869 2.454192 0.108481
H 5.126865 2.205203 1.692115
H 3.999486 1.921435 3.023466
C 4.115514 0.288079 1.635502
C 3.899131 -0.597323 2.709205
C 4.360077 -0.261272 0.350880
C 3.928140 -1.986610 2.547382
H 3.709966 -0.171240 3.700364
C 4.376781 -1.662113 0.190869
C 4.173524 -2.521121 1.276009
H 3.774173 -2.642991 3.410188
H 4.579746 -2.097799 -0.793575
H 4.212825 -3.604502 1.123505
C 1.377435 -1.666743 -2.899464
H 1.641499 -0.721766 -3.401048
H 2.261988 -2.008528 -2.334150
H 1.105502 -2.429964 -3.641961
C 0.169862 -3.386894 2.585789
H 0.007741 -4.455023 2.384369
H 1.246636 -3.152273 2.591725
H -0.230801 -3.135733 3.582621
C -4.952962 -0.276040 2.229486
H -4.646517 -0.102730 3.273501
H -5.372895 0.669206 1.843671
H -5.703957 -1.076527 2.177803
C -3.822100 1.333069 -2.910776
H -4.443354 1.984683 -2.272200
H -3.146380 1.980332 -3.492522
H -4.456767 0.738698 -3.582722
H -1.888667 2.777068 0.912499
O -2.483557 3.877497 1.018542
C -3.757120 3.701801 0.647093
O -4.274740 2.638884 0.301868
C -4.509183 5.026542 0.680680
H -4.202502 5.642882 -0.182095
H -5.590174 4.838794 0.621255
H -4.268011 5.595156 1.592512

I 4.596195 0.921726 -1.304853

Egas = -1926.61993531
One imaginary frequency -550.3882
Zero-point correction= 0.414389
Sum of electronic and thermal Enthalpies= -1926.163963
Sum of electronic and thermal Free Energies= -1926.284915
Gsolv= -1926.883193

S5-12. By-products formation for Substrate **A** (Scheme S2-1, S2-2 & Figure S2-1).

α-C-H amination TS for substrate A

Rh -0.792032 -0.166889 -0.582436
Rh -2.378694 0.439181 1.137468
O 0.567796 -0.599731 0.905718
O -0.955347 -0.059402 2.533914
O -2.228326 0.316201 -1.980625
O -3.721673 0.901170 -0.335730
O -0.179650 1.801623 -0.553578
O -1.680038 2.350812 1.080495
O -1.546969 -2.063763 -0.435098
O -3.062713 -1.473377 1.171533
C 0.189370 -0.469426 2.132359
C -0.747426 2.633826 0.249131
C -3.369742 0.734320 -1.559917
C -2.504836 -2.319199 0.382378
N 0.476010 -0.633431 -2.001731
C 1.499110 -1.530755 -1.822086
O 1.280140 -2.724586 -1.639708
O 2.847148 -1.111432 -2.032815
O 3.069373 0.251113 -1.985617
O 3.317695 0.878514 -0.632710
H 1.453596 0.720307 -2.364792
H 3.676077 0.591172 -2.836391
H 3.241282 1.974411 -0.736692
H 2.531645 0.545399 0.065235
C 4.701629 0.490988 -0.127063
C 4.927374 -0.790203 0.416107
C 5.779449 1.392674 -0.214012
C 6.198781 -1.150399 0.880787
H 4.100520 -1.506656 0.468045
C 7.051919 1.032608 0.251236
H 5.615385 2.390512 -0.637812
C 7.264352 -0.240241 0.800562
H 6.358692 -2.146916 1.305507
H 7.877668 1.748909 0.186614
H 8.257707 -0.523291 1.163648
C 1.195954 -0.871006 3.187022
H 1.149507 -1.965150 3.327864
H 2.217251 -0.617850 2.863255
H 0.961898 -0.384096 4.144567
C -0.269969 4.065722 0.184698
H 0.828971 4.101625 0.257689
H -0.551348 4.502199 -0.788702
H -0.721960 4.655744 0.994105
C -3.021795 -3.737491 0.386846
H -3.655307 -3.894722 -0.503235
H -2.180783 -4.444988 0.324873
H -3.620385 -3.923029 1.289978
C -4.406516 1.029554 -2.620686
H -3.922841 1.406774 -3.533887
H -4.933961 0.094228 -2.877618
H -5.144310 1.752578 -2.243632

Egas = -1687.08488998
One imaginary frequency of -1371.0050
Zero-point correction= 0.359746
Sum of electronic and thermal Enthalpies= -1686.690436
Sum of electronic and thermal Free Energies= -1686.795062
Gsolv= -1687.226221

2-phenylacetaldehyde

C -2.386792 -0.228226 -0.474260
O -3.285059 0.595761 -0.464274
H -2.127828 -0.799700 -1.407202
C -1.499771 -0.557497 0.723491
H -1.859055 0.028028 1.585948
H -1.619298 -1.633812 0.952628
C -0.048878 -0.257774 0.393610
C 0.403190 1.076628 0.368136
C 0.851792 -1.283842 0.053812
C 1.728243 1.374984 0.026941
H -0.293362 1.884343 0.620322
C 2.178158 -0.986716 -0.290105
H 0.513163 -2.326348 0.069731
C 2.620185 0.343534 -0.303662
H 2.065420 2.416732 0.018304
H 2.868177 -1.798156 -0.544152
H 3.656497 0.576285 -0.569165

E(RPBE-PBE) = -384.392833616
No imaginary frequency
Zero-point correction=0.134233
Sum of electronic and thermal Enthalpies= -384.249530
Sum of electronic and thermal Free Energies= -384.293251
Gsolv= -384.4033884

Hydride transfer TS from Salt-K_A to ^1NR_A

Rh -0.963898 -0.703066 0.269455
Rh -0.573664 -3.093623 0.486024
O -2.042271 -0.784208 2.022513
O -1.713049 -3.051433 2.190868
O 0.049371 -0.786775 -1.525654
O 0.527745 -3.013707 -1.260560
O -2.675818 -1.172536 -0.796427
O -2.259642 -3.424377 -0.622277
O 0.706374 -0.415258 1.448669
O 1.087172 -2.666470 1.636060
C -2.186018 -1.928670 2.592670
C -2.941481 -2.411501 -1.015915
C 0.541627 -1.902841 -1.913962
C 1.322822 -1.438017 1.922302
N -1.058693 1.403334 0.233807
C -1.888872 2.243515 -0.475404
O -2.310304 3.339977 -0.088770
O -2.106936 1.751764 -1.746670
C -2.973674 2.533691 -2.595897
C -4.359694 1.879929 -2.737221
H -2.474373 2.558049 -3.580234
H -3.051178 3.554812 -2.187408
H -4.851431 2.354891 -3.607650
H -4.204116 0.816280 -2.994780
C -5.251261 2.006803 -1.518371
C -5.097134 1.141051 -0.416647
C -6.238996 3.007735 -1.454718
C -5.906889 1.282970 0.719155
H -4.331000 0.358616 -0.450075
C -7.052163 3.147733 -0.321180
H -6.373543 3.685587 -2.306740
C -6.887223 2.284841 0.771975
H -5.766160 0.605399 1.568825
H -7.817204 3.931370 -0.293222
H -7.520598 2.392463 1.659062
C -2.972444 -1.939962 3.887433
H -2.297810 -1.691879 4.725816
H -3.768669 -1.180817 3.858793
H -3.396859 -2.938379 4.067993
C -4.176639 -2.693381 -1.847299
H -4.994149 -2.014061 -1.558269
H -3.949445 -2.508883 -2.912060
H -4.489072 -3.740875 -1.729405
C 2.398198 -1.158737 2.952430
H 2.919653 -0.217281 2.725220
H 1.921659 -1.064258 3.944859
H 3.116426 -1.990807 2.998213
C 1.229933 -1.901682 -3.264361
H 0.647714 -1.297094 -3.977517
H 2.230109 -1.442203 -3.172184

H 1.338733 -2.928807 -3.642643
H -1.050231 1.743225 1.203791
C 0.984047 2.163553 -1.085460
H 0.216195 1.837883 -1.791982
O 1.985968 1.312323 -1.022927
C 1.014924 3.560453 -0.566534
H -0.029307 3.927825 -0.576916
H 1.568521 4.159745 -1.322042
C 3.340439 1.732265 -0.659896
O 3.765703 2.828785 -1.062285
N 4.085667 0.862945 0.022039
O 3.506692 -0.493139 0.068196
S 4.580639 -1.388223 -0.845790
O 5.944662 -1.317386 -0.259337
O 4.478667 -0.962256 -2.270322
C 3.858282 -3.004486 -0.568259
H 3.911772 -3.217955 0.508651
H 2.808833 -3.015080 -0.903876
H 4.469458 -3.718848 -1.140118
K 6.223176 1.439605 -1.414883
C 1.638901 3.819486 0.799205
C 2.163215 5.098470 1.066622
C 1.649124 2.854318 1.823217
C 2.674047 5.416062 2.331016
H 2.163934 5.856104 0.274138
C 2.169128 3.172312 3.088004
H 1.264781 1.845665 1.639843
C 2.678874 4.450700 3.349942
H 3.070072 6.419518 2.520626
H 2.170080 2.408284 3.873215
H 3.077002 4.695548 4.340292

Egas = -3503.03069268
One imaginary frequency -73.6880
Zero-point correction= 0.582767
Sum of electronic and thermal Enthalpies= -3502.393630
Sum of electronic and thermal Free Energies= -3502.541394
Gsolv= -3503.321519

INT'$_A$

Rh -1.959874 -0.518025 -0.029596
Rh -4.185446 0.378734 -0.424896
O -2.246158 -0.110670 1.982819
O -4.390657 0.620171 1.602120
O -1.783718 -0.803693 -2.063614
O -3.866808 0.089590 -2.431054
O -1.233521 1.440491 -0.193972
O -3.349083 2.242635 -0.578079
O -2.819855 -2.369072 0.143066
O -4.919590 -1.524530 -0.253923
C -3.391964 0.350783 2.355554
C -2.087404 2.381796 -0.437961
C -2.758701 -0.421888 -2.817505
C -4.094906 -2.473295 0.001680
N -0.036090 -1.476828 0.099770
C 0.443419 -2.146589 1.171917
O 0.357902 -1.793517 2.363192
O 1.164741 -3.298293 0.809250
C 1.813984 -3.978616 1.898638
C 3.286629 -3.556228 2.069139
H 1.765150 -5.052058 1.647577
H 1.246315 -3.788655 2.824240
H 3.722893 -4.223134 2.838203
H 3.827456 -3.760941 1.127558
C 3.489793 -2.108149 2.466901
C 3.283639 -1.685852 3.795442
C 3.868996 -1.145736 1.511540
C 3.442544 -0.341955 4.159051
H 2.986697 -2.420594 4.552774
C 4.030864 0.203635 1.867714
H 4.058060 -1.455563 0.478001
C 3.815508 0.612004 3.194805
H 3.286995 -0.038278 5.200329
H 4.341273 0.934303 1.113933
H 3.962961 1.661331 3.471232
C -3.553773 0.623094 3.838067
H -2.982235 -0.110589 4.426695
H -3.165390 1.630718 4.072370
H -4.617430 0.595529 4.116377
C -1.534295 3.781968 -0.604657

H -0.643350 3.928450 0.024510
H -1.233917 3.921505 -1.658026
H -2.308872 4.525263 -0.365090
C -4.676987 -3.858738 0.190036
H -3.982938 -4.621851 -0.193734
H -4.822832 -4.046521 1.268365
H -5.652032 -3.935688 -0.312638
C -2.549121 -0.580375 -4.309148
H -1.773124 0.129358 -4.645072
H -2.191816 -1.597909 -4.536470
H -3.483003 -0.379136 -4.852474
H 0.027820 -2.061038 -0.741850
C 1.881322 -0.326759 -2.068709
H 0.790159 -0.214887 -2.254227
C 2.453386 -1.700698 -2.317774
H 1.957737 -2.059892 -3.245613
H 2.043965 -2.353770 -1.519749
C 3.956410 -1.859948 -2.404087
C 4.558052 -3.004408 -1.845544
C 4.771291 -0.935349 -3.085852
C 5.938781 -3.221215 -1.957527
H 3.931099 -3.737060 -1.323819
C 6.152033 -1.148646 -3.195123
H 4.327459 -0.038007 -3.526622
C 6.741057 -2.290530 -2.631852
H 6.386018 -4.117334 -1.515038
H 6.771001 -0.416810 -3.724467
H 7.820218 -2.453135 -2.718559
O 2.551872 0.658433 -1.733309
C 1.380498 2.460336 -2.156826
O 0.545649 2.068069 -2.909728
N 1.982609 3.433195 -1.592047
O 3.106425 3.138470 -0.758610
S 2.875009 3.850687 0.763262
O 4.156344 3.608721 1.440096
O 1.596284 3.377113 1.346134
C 2.694669 5.590384 0.334172
H 3.656055 5.941329 -0.066259
H 1.896668 5.674167 -0.417310
H 2.430469 6.126290 1.257701
K 0.602163 0.774297 1.770284

Egas = -3503.08098837
No imaginary frequency
Zero-point correction= 0.580586
Sum of electronic and thermal Enthalpies= -3502.443579
Sum of electronic and thermal Free Energies= -3502.598817
Gsolv= -3503.360598

TS Regeneration from INT'$_A$

Rh -1.707104 -0.250562 -0.196534
Rh -3.983241 -0.366474 0.590623
O -1.150807 -1.309179 1.478984
O -3.314812 -1.382570 2.238679
O -2.400236 0.795962 -1.828828
O -4.567955 0.653119 -1.087883
O -1.514362 1.545620 0.823695
O -3.683011 1.409319 1.566734
O -2.009343 -2.027095 -1.175789
O -4.173457 -2.140008 -0.422743
C -2.058382 -1.630278 2.330546
C -2.539284 1.978178 1.475059
C -3.665247 1.005239 -1.930365
C -3.165693 -2.588134 -1.075352
N 0.397253 -0.341819 -0.948714
C 1.468676 0.096118 -0.017013
O 1.753534 -0.192477 1.128531
O 3.139412 -0.768937 -1.282551
C 3.036755 -2.134488 -1.161841
C 4.370884 -2.868483 -1.525294
H 2.247117 -2.590945 -1.824520
H 2.775837 -2.465505 -0.116171
H 4.211625 -3.959383 -1.429169
H 4.612627 -2.650875 -2.580231
C 5.494060 -2.412736 -0.629718
C 5.566854 -2.848197 0.712971
C 6.426149 -1.443048 -1.059778
C 6.523442 -2.325095 1.598333
H 4.856206 -3.603913 1.067743
C 7.389565 -0.918273 -0.180808

H 6.384982 -1.092911 -2.096796
C 7.437396 -1.350607 1.156469
H 6.564148 -2.686586 2.631760
H 8.107875 -0.173956 -0.542138
H 8.193198 -0.950997 1.840836
C -1.588203 -2.345813 3.578931
H -0.751426 -3.019461 3.339932
H -1.223195 -1.600146 4.306888
H -2.417138 -2.905028 4.036566
C -2.372611 3.302605 2.193028
H -1.447091 3.295054 2.791754
H -2.290400 4.117846 1.452964
H -3.236954 3.497956 2.843052
C -3.331681 -3.912246 -1.788849
H -2.929780 -3.842986 -2.812228
H -2.756178 -4.690123 -1.257855
H -4.391018 -4.203912 -1.815217
C -4.135040 1.711483 -3.183990
H -5.014072 2.335972 -2.964281
H -3.323506 2.318372 -3.611269
H -4.432814 0.956836 -3.933075
H 0.514408 -1.292003 -1.329857
C 0.824479 0.741363 -1.847015
O 0.623533 1.014153 -2.997015
N 1.515335 1.363633 -0.761692
O 2.658774 2.128201 -0.993153
S 2.763546 3.465762 0.118788
O 3.661883 4.403887 -0.565427
O 3.135486 2.872205 1.423282
C 1.070854 4.078297 0.179250
H 0.822938 4.477094 -0.815100
H 0.391649 3.257553 0.460328
H 1.071647 4.878191 0.935393
K 4.428056 0.259088 0.755756

Egas = -3118.63860751
One imaginary frequency -85.5991
Zero-point correction= 0.443080
Sum of electronic and thermal Enthalpies= -3118.149333
Sum of electronic and thermal Free Energies= -3118.280307
Gsolv= -3118.934131

Products from INT'$_A$ via Regeneration Pathway

Rh -2.154652 0.559898 -0.038831
Rh -3.422655 -1.465326 0.273412
O -0.942882 -0.491153 -1.358423
O -2.228836 -2.374565 -1.123641
O -3.407787 1.470559 1.308423
O -4.586020 -0.470878 1.631173
O -0.902022 -0.080026 1.503728
O -2.136696 -1.992319 1.781442
O -3.454429 1.055890 -1.541530
O -4.670957 -0.863439 -1.227734
C -1.258973 -1.716030 -1.637090
C -1.168518 -1.212181 2.073198
C -4.347347 0.772316 1.843760
C -4.420754 0.251278 -1.814036
N -1.059907 2.512770 -0.392578
C -1.707573 3.537104 -0.149450
O -2.372226 4.480548 0.118282
O 5.339888 0.656129 -1.044898
C 6.342038 0.469942 -0.009832
C 6.309731 1.709079 0.891782
H 7.320057 0.356161 -0.508201
H 6.118181 -0.447397 0.555839
H 7.144943 1.614783 1.610320
H 6.512791 2.601824 0.273808
C 4.992395 1.862609 1.626153
C 4.797588 1.279194 2.893983
C 3.917819 2.554643 1.031859
C 3.564931 1.383779 3.555171
H 5.625662 0.742877 3.373256
C 2.684041 2.668565 1.693826
H 4.044874 2.997186 0.038098
C 2.502090 2.080231 2.956875
H 3.438384 0.931663 4.544705
H 1.862921 3.209195 1.211416
H 1.541150 2.175835 3.473709
C -0.385521 -2.438197 -2.636662
H 0.180924 -1.721391 -3.249451
H 0.326970 -3.093290 -2.103147

H -1.007985 -3.081289 -3.277959
C -0.213471 -1.667549 3.155792
H 0.169861 -0.803585 3.720688
H -0.713885 -2.376516 3.831203
H 0.645805 -2.181440 2.686930
C -5.329321 0.653229 -2.954370
H -5.513051 1.738366 -2.929605
H -4.834693 0.418367 -3.913089
H -6.278155 0.100388 -2.902246
C -5.273542 1.501890 2.790872
H -5.752529 0.792639 3.481371
H -4.721786 2.273786 3.348257
H -6.062166 2.006762 2.205682
H -0.092229 2.526961 -0.918244
C 4.520094 -0.366187 -1.356990
O 3.482396 -0.033513 -2.094155
N 4.812036 -1.576133 -0.927186
O 3.793143 -2.497598 -1.437097
S 3.059877 -3.298179 -0.175292
O 2.133318 -4.233514 -0.831269
O 2.550529 -2.311015 0.814756
C 4.435485 -4.186535 0.570730
H 4.763488 -4.956884 -0.141238
H 5.235948 -3.457199 0.760207
H 4.073180 -4.639051 1.505253
K 1.444955 0.089264 0.042435
O 3.127873 2.439442 -2.718823
C 2.010917 2.863538 -2.294020
O 1.308825 2.247930 -1.406106
C 1.466577 4.171979 -2.853121
H 0.508772 3.982581 -3.368704
H 1.259686 4.880794 -2.032562
H 2.175733 4.624120 -3.560622
H 3.423829 0.990369 -2.298649

Egas = -3347.55626819
No imaginary frequency
Zero-point correction= 0.504146
Sum of electronic and thermal Enthalpies= -3346.998965
Sum of electronic and thermal Free Energies= -3347.147591
Gsolv= -3347.896051

TS Protonation from INT'$_A$

Rh 2.441497 -0.454535 -0.594993
Rh 4.611226 0.215858 0.185946
O 1.741235 -0.133801 1.337105
O 3.834274 0.451331 2.059993
O 3.265721 -0.698266 -2.457232
O 5.337215 -0.065993 -1.703186
O 2.945362 -2.384215 -0.125926
O 5.012493 -1.736629 0.628801
O 2.021153 1.534716 -0.986504
O 4.125948 2.150923 -0.315093
C 2.588602 0.222157 2.248762
C 4.101496 -2.611236 0.389311
C 4.517329 -0.453332 -2.613811
C 2.965040 2.396406 -0.794495
N -3.453092 0.434007 -0.393514
C -3.239888 1.419667 -1.310720
O -2.158555 1.632974 -1.875811
O -4.352462 2.213860 -1.436253
C -4.155388 3.476932 -2.107747
C -4.127957 4.630770 -1.090973
H -5.015674 3.590356 -2.788205
H -3.222231 3.427818 -2.691926
H -4.131558 5.575121 -1.666915
H -5.068294 4.604635 -0.510809
C -2.937835 4.599500 -0.153850
C -1.766298 5.323893 -0.449380
C -2.968114 3.830520 1.027641
C -0.656468 5.284848 0.407066
H -1.728041 5.935385 -1.358654
C -1.859879 3.783947 1.889598
H -3.871005 3.262418 1.277039
C -0.697455 4.511032 1.579310
H 0.235308 5.874898 0.169569
H -1.904148 3.174899 2.798556
H 0.161132 4.493589 2.259945
C 2.059218 0.374254 3.653662
H 2.869853 0.666228 4.335621
H 1.253046 1.126449 3.680464

H 1.599970 -0.572786 3.982103
C 4.401546 -4.042389 0.773763
H 3.908409 -4.269784 1.735045
H 3.995725 -4.732398 0.018152
H 5.484929 -4.190182 0.888464
C 2.673251 3.820864 -1.211680
H 2.829559 3.913917 -2.300949
H 1.622782 4.078264 -1.003884
H 3.351828 4.518401 -0.699617
C 5.067852 -0.607183 -4.014509
H 4.575846 -1.447189 -4.527674
H 4.856734 0.311106 -4.590272
H 6.156701 -0.757473 -3.983829
H -4.400172 0.162328 -0.104348
C -5.265142 -2.146374 1.508748
H -5.146052 -2.673980 2.487615
C -5.426868 -3.095588 0.336884
H -6.322603 -3.708513 0.579221
H -4.575295 -3.803413 0.383362
C -5.541942 -2.469700 -1.032576
C -4.683360 -2.884587 -2.068707
C -6.506542 -1.479587 -1.308545
C -4.782102 -2.321892 -3.350258
H -3.933152 -3.654159 -1.856095
C -6.598128 -0.909080 -2.585955
H -7.184577 -1.148183 -0.515811
C -5.736458 -1.327667 -3.611185
H -4.114881 -2.664695 -4.148905
H -7.348982 -0.136420 -2.781856
H -5.812895 -0.885443 -4.609897
O -5.312342 -0.919725 1.471381
C -1.910700 -2.867467 0.547532
O -2.104813 -3.966996 0.037893
N -2.365649 -2.403108 1.700768
O -1.963329 -1.000335 1.898940
S -1.690400 -0.662312 3.500782
O -1.392542 0.790735 3.422800
O -0.735090 -1.591621 4.126279
C -3.317707 -0.920531 4.227337
H -4.060536 -0.375051 3.626397
H -3.513831 -2.001677 4.198955
H -3.275104 -0.556494 5.264496
K -0.554308 1.069786 0.375721
H -2.670496 -0.646790 -0.585924
O -1.069870 -1.790876 -0.226963
C -0.376189 -2.024048 -1.368191
O 0.602739 -1.301951 -1.613702
C -0.840998 -3.056495 -2.354500
H -0.951467 -4.029887 -1.852349
H -1.840926 -2.781449 -2.732648
H -0.123632 -3.103470 -3.184673

Egas = -3731.90658997
One imaginary frequency -286.3714
Zero-point correction= 0.637541
Sum of electronic and thermal Enthalpies= -3731.207219
Sum of electronic and thermal Free Energies= -3731.373347
Gsolv= -3732.210147

Products from INT'$_A$ via Protonation Pathway

Rh 2.632436 -0.476429 -0.597596
Rh 4.823662 -0.147349 0.336718
O 1.874744 -0.281255 1.326079
O 3.980284 -0.035637 2.194791
O 3.514534 -0.620212 -2.443396
O 5.603203 -0.295931 -1.550471
O 2.900378 -2.487697 -0.338659
O 4.987224 -2.170053 0.560662
O 2.463503 1.589840 -0.790458
O 4.577779 1.871702 0.052987
C 2.707996 -0.141707 2.304978
C 3.997643 -2.901234 0.189691
C 4.793083 -0.512454 -2.523876
C 3.489965 2.303248 -0.459694
N -4.360748 0.392779 -0.142318
C -3.654496 1.192285 -0.973877
O -2.431421 1.121546 -1.180656
O -4.468494 2.140640 -1.546490

C -3.830334 3.101433 -2.410328
C -3.681216 4.465736 -1.713610
H -4.496811 3.200711 -3.283290
H -2.853077 2.705863 -2.731518
H -3.405045 5.200603 -2.492550
H -4.673924 4.764530 -1.329904
C -2.658849 4.495599 -0.596483
C -1.358681 4.988658 -0.822562
C -2.977852 4.017958 0.691782
C -0.404384 5.010908 0.207255
H -1.095605 5.376218 -1.813933
C -2.024882 4.024556 1.721585
H -3.984907 3.631625 0.884413
C -0.732725 4.524366 1.483817
H 0.589654 5.430693 0.018875
H -2.282224 3.634661 2.710914
H 0.003763 4.550781 2.294269
C 2.120439 -0.121807 3.695447
H 2.895033 0.120158 4.436513
H 1.287065 0.597087 3.751723
H 1.686501 -1.111545 3.920047
C 4.115907 -4.390737 0.421872
H 3.638367 -4.644860 1.384524
H 3.591515 -4.944224 -0.371490
H 5.173509 -4.688609 0.468288
C 3.412123 3.788423 -0.742804
H 3.888439 3.990644 -1.718441
H 2.366140 4.124730 -0.797066
H 3.962245 4.353709 0.024533
C 5.402421 -0.688099 -3.897719
H 5.599546 -1.760825 -4.070292
H 4.704525 -0.341313 -4.674333
H 6.357843 -0.147250 -3.964869
H -5.376193 0.357683 -0.219114
C -5.433001 -2.717622 1.734928
H -4.952766 -3.326635 2.543689
C -5.517323 -3.445160 0.407591
H -6.155178 -4.337466 0.588742
H -4.499536 -3.842193 0.218709
C -6.005070 -2.632928 -0.767991
C -5.220609 -2.551196 -1.934576
C -7.229313 -1.934870 -0.726463
C -5.647225 -1.794170 -3.035826
H -4.262153 -3.080739 -1.964569
C -7.652152 -1.169520 -1.823513
H -7.849521 -1.983701 0.173774
C -6.862744 -1.096645 -2.982167
H -5.027566 -1.750096 -3.938298
H -8.605671 -0.632558 -1.775022
H -7.197459 -0.503096 -3.839442
O -5.839411 -1.585037 1.966765
C -1.903730 -2.311565 0.224941
O -2.221719 -3.375294 -0.308574
N -2.597682 -1.656833 1.161544
O -1.903455 -0.400776 1.531698
S -1.866276 -0.146258 3.176667
O -1.090411 1.122587 3.231671
O -1.427762 -1.336061 3.920627
C -3.589921 0.210281 3.554348
H -3.874943 1.117497 3.002804
H -4.222466 -0.633174 3.235903
H -3.655994 0.369687 4.641173
K -0.188512 1.347705 0.301911
H -3.890325 -0.442875 0.239701
O -0.624492 -1.647964 -0.174480
C -0.317366 -1.453785 -1.462015
O 0.741136 -0.848980 -1.731814
C -1.240686 -1.894012 -2.564630
H -1.428535 -2.975340 -2.487750
H -2.211893 -1.380865 -2.459337
H -0.782678 -1.634349 -3.528279

Egas = -3731.95967322
No imaginary frequency
Zero-point correction= 0.644096
Sum of electronic and thermal Enthalpies= -3731.253487
Sum of electronic and thermal Free Energies= -3731.421178
Gsolv= -3732.290091

Phenethyl carbamate

N 3.164651 1.646157 -0.130434
C 2.834366 0.319835 -0.296442
O 3.453947 -0.486340 -0.978981
O 1.676002 0.045650 0.387787
C 1.190488 -1.302549 0.202380
C -0.109428 -1.445419 0.997872
H 1.955000 -2.016213 0.557469
H 1.028816 -1.487702 -0.873889
H -0.390986 -2.514107 0.967839
H 0.098839 -1.200128 2.055326
C -1.249586 -0.596817 0.471968
C -1.402163 0.742504 0.877967
C -2.165389 -1.122833 -0.458593
C -2.440564 1.533668 0.369090
H -0.694508 1.165099 1.599276
C -3.205313 -0.335006 -0.971169
H -2.064106 -2.166006 -0.781867
C -3.346177 0.997253 -0.557942
H -2.544774 2.572752 0.699427
H -3.909810 -0.765018 -1.691088
H -4.160082 1.613649 -0.953664
H 2.734628 2.162127 0.633247
H4.117436 1.894142 -0.380267

Egas = -554.129157890
No imaginary frequency
Zero-point correction= 0.184896
Sum of electronic and thermal Enthalpies= -553.931783
Sum of electronic and thermal Free Energies= -553.984465
Gsolv= -554.155287
